# Identifying the fundamental structures and processes of care contributing to emergency general surgery quality using a mixed-methods Donabedian approach

**DOI:** 10.1186/s12874-020-01096-7

**Published:** 2020-10-02

**Authors:** Heena P. Santry, Scott A. Strassels, Angela M. Ingraham, Wendelyn M. Oslock, Kevin B. Ricci, Anghela Z. Paredes, Victor K. Heh, Holly E. Baselice, Amy P. Rushing, Adrian Diaz, Vijaya T. Daniel, M. Didem Ayturk, Catarina I. Kiefe

**Affiliations:** 1 Department of Surgery, Ohio State University Wexner Medical Center, 410 W 10th Avenue, Columbus, OH USA; 2 Center for Surgical Health Assessment, Research, and Policy, The Ohio State University, 395 W 12th Avenue, Columbus, OH USA; 3 Department of Surgery, The Ohio State University Wexner Medical Center, 395 W 12th Ave, Suite 614, Columbus, OH 43210 USA; 4 Department of Surgery, University of Wisconsin, 600 Highland Avenue, Madison, WI USA; 5 Department of Surgery, University of Massachusetts Medical School, 55 N Lake Avenue, Worcester, MA USA; 6 Department of Quantitative Health Sciences, University of Massachusetts Medical School, 55 N Lake Avenue, Worcester, MA USA

**Keywords:** Emergency general surgery, Quality of care/patient safety, Patient Outcomes, Health care organizations and systems, Resource use / survey research and questionnaire design / administrative data uses

## Abstract

**Background:**

Acute Care Surgery (ACS) was developed as a structured, team-based approach to providing round-the-clock emergency general surgery (EGS) care for adult patients needing treatment for diseases such as cholecystitis, gastrointestinal perforation, and necrotizing fasciitis. Lacking any prior evidence on optimizing outcomes for EGS patients, current implementation of ACS models has been idiosyncratic. We sought to use a Donabedian approach to elucidate potential EGS structures and processes that might be associated with improved outcomes as an initial step in designing the optimal model of ACS care for EGS patients.

**Methods:**

We developed and implemented a national survey of hospital-level EGS structures and processes by surveying surgeons or chief medical officers regarding hospital-level structures and processes that directly or indirectly impacted EGS care delivery in 2015. These responses were then anonymously linked to 2015 data from the American Hospital Association (AHA) annual survey, Medicare Provider Analysis and Review claims (MedPAR), 17 State Inpatient Databases (SIDs) using AHA unique identifiers (AHAID). This allowed us to combine hospital-level data, as reported in our survey or to the AHA, to patient-level data in an effort to further examine the role of EGS structures and processes on EGS outcomes. We describe the multi-step, iterative process utilizing the Donabedian framework for quality measurement that serves as a foundation for later work in this project.

**Results:**

Hospitals that responded to the survey were primarily non-governmental and located in urban settings. A plurality of respondent hospitals had fewer than 100 inpatient beds. A minority of the hospitals had medical school affiliations.

**Discussion:**

Our results will enable us to develop a measure of preparedness for delivering EGS care in the US, provide guidance for regionalized care models for EGS care, tiering of ACS programs based on the robustness of their EGS structures and processes and the quality of their outcomes, and formulate triage guidelines based on patient risk factors and severity of EGS disease.

**Conclusions:**

Our work provides a template for team science applicable to research efforts combining primary data collection (i.e., that derived from our survey) with existing national data sources (i.e., SIDs and MedPAR).

## Background

In the 1960s, physician-researcher Avedis Donabedian proposed a framework for assessing the quality of health care by evaluating three elements—structure, process, and outcomes [1]. “Structure” refers to the setting in which care occurs; “process” refers to how care is delivered; and “outcomes” refers to the effects of care on the health of the patient and the population. Since its introduction, the Donabedian Model has dominated the national discourse on health care quality. Its flexibility has allowed the model to be useful in quality improvement initiatives across clinical settings. The model has been used to improve surgical quality overall [2–4] and for specific diseases such as lung cancer [5], prostate cancer [6], congenital heart defects [7], and morbid obesity [8]. Within trauma care, the model has influenced the development of structure and process measures required for trauma center verification [9] and generating clinical protocols such as for cervical spine clearance [10].

In 2006, the Institute of Medicine (IOM), now the National Academy of Medicine, described the state of hospital-based emergency care in the US as being at its “breaking point”, [11, 12] burdened by overcrowded emergency rooms, uncompensated care for common, non-emergent conditions, and lack of appropriate specialty providers. Inadequate access to specialty care for non-trauma general surgery emergencies (i.e., common diseases, such as appendicitis, cholecystitis, and abscesses; and complex diseases, such as perforated viscus, ischemic enteritis, and necrotizing soft tissue infections) was deemed a major stressor. While all such patients require urgent surgical evaluation and only about 30% require emergency operation, their conditions are collectively referred to as emergency general surgery (EGS) diseases. The more than 3 million Americans are hospitalized in the US annually for EGS disease account for 7.1% of all hospitalizations (exceeding traumatic injury, cerebrovascular accident, and acute myocardial infarction) [13, 14]. Annual incidence of EGS disease (1290/100,000) surpasses new diagnoses of diabetes (900/100,000) and cancer (650/100,000) [13], while costs of EGS care in the US each year range exceed $28 billion, outpacing the cost of traumatic injury by $9 billion and acute myocardial infarction by $17.3 billion [15]. Among those patients whose need for urgent or emergency operation is recognized in a timely fashion, complication rates exceed 33%, 30-day readmission rates exceed 15%, and mortality exceeds 9% [14, 16–25]. Despite the clear individual and public health burden, 37% of emergency room directors surveyed in 2010 reported inadequate general surgery coverage for patients presenting acute-onset abdominal or skin/soft-tissue conditions [26].

Fortunately, 5 years previously a new specialty called Acute Care Surgery (ACS) had been proposed by leading trauma surgeons in the US as a solution to the crisis in access to emergency general surgery (EGS) care [27–29]. The lessons learned from trauma care were highly influential during the initial proposals for ACS. In 1966 the IOM declared unintentional injury the, “neglected disease of modern society” [30]. In response, surgeons and policymakers put forward substantial effort to address the consequences of acute traumatic injury. As a result, tiered systems of trauma care became standard in the US. Within these systems regulated by state and national policies, the *structures* and *processes* of care for injured patients are protocol-driven and regionalized while systematic *outcomes* measurement and continuous quality improvement are mandated [31–42]. The decreases in injury-related mortality attributable to defining structures and processes in trauma care have been lauded as a significant achievement of twentieth century US health policy [11, 31, 38, 39, 43, 44]. Presently, the Trauma Verification, Review, and Consultation Program of the American College of Surgeons is used in most US states to ensure adequate structures and processes for the delivery of trauma care [45]. Simultaneously, outcomes are monitored and benchmarked through programs such as the Trauma Quality Improvement Program (TQIP) [46]. While no such structures and processes previously existed for emergency general surgery (EGS) patients previously, the creation of ACS models of care was theorized to provide some of these same quality benefits to EGS patients through a structured, team-based approach to round-the-clock EGS care [27–29].

The ACS model has been spreading in the decade since it was first described, largely consistent with the Rogers’ Diffusion of Innovation Theory [47–49], and has been associated with improved outcomes such as lower emergency room wait times, faster time to the operating room, better operating room efficiency, shorter length of stay (LOS), fewer postoperative complications, and lower mortality at centers that were “early adopters” [50–59]. However, the Donabedian Model has not been applied to EGS care as a means to sustain these outcome benefits. Importantly, lacking any prior evidence on optimizing outcomes for EGS patients, current implementation of ACS models has been idiosyncratic. Therefore, we sought to elucidate which structures and processes in the care of EGS patients are associated with improved outcomes as an initial step in designing the optimal model of ACS care for EGS patients. This manuscript reviews our rationale and methods in measuring EGS quality using the Donabedian Model as a framework in a multimodal health services research approach combining survey research and large database epidemiology. The findings of this overall body of work will have implications for establishing the requisite structures and processes necessary to optimize EGS outcomes at the institutional level and implementing ACS models of care regionally much like those that already exist for other emergency conditions such as traumatic injury, acute myocardial infarction, and cerebrovascular accident.

## Methods

Data for this study were derived by combining validated administrative data with responses to an original survey regarding hospital-level structures and processes that may impact EGS care delivery. Our goal was to measure the relationship between EGS structures and processes and patient-level outcomes in individuals aged 18 years and older and Medicare beneficiaries aged 65+. This study was approved by the University of Massachusetts Medical School and Ohio State College of Medicine Institutional Review Boards.

### Survey development

The questionnaire was developed iteratively, starting with semi-structured interviews that were then used to create a pilot questionnaire before the study survey was finalized. Prior to designing the pilot survey, the lead investigator interviewed (template shown in Additional file 1) a convenience sample of senior surgeons who were responsible for implementing ACS models of care at their hospitals. Interviewees represented three different practice settings (university-based, public safety-net, private community based) in each of 6 different geographic areas (New England, Northeast, Mid-Atlantic, Midwest, South, West) [60–62]. Results from qualitative analyses and published reports from early adopters of ACS were then used to inform the pilot questionnaire development. This pilot questionnaire (Additional file 2) was sent to surgeons responsible for EGS care at all University Health Systems Consortium (UHC, now Vizient) hospitals in 2012 (*n* = 319) [63]. The final response rate was 81%. The questionnaire responses yielded important information on variations in the implementation, if any, of ACS care across university-affiliated hospitals and key differences between ACS hospitals compared to those using a traditional general surgeon on call (GSOC) model [49, 64].

This formative work provided estimates of the construct validity and reliability of the questionnaire items and possible scalability of the questionnaire to a nationally representative survey. Importantly, the pilot survey had used self-reported adoption of ACS to initiate a skip sequence after which additional questions on EGS structures and processes were asked depending on the initial response to whether or not ACS was in place at the responding hospital. Given that this might elicit social desirability bias in responses by unmasking our goal to compare EGS delivered through ACS models of care vs GSOC model, we opted to remove the skip sequence in creating the national survey. Rather, we elicited multiple responses on the exact structures and processes that might affect EGS care, whether or not an ACS model had been implemented, and asked respondents to self-identify as ACS, GSOC, or other only at the close of the survey. This new questionnaire draft was pilot-tested in an iterative fashion with 26 surgeons who participate in the care of EGS patients at their institutions but who would not be asked to respond to the final survey. These surgeons were located at hospitals around the country ranging in size and scope of practice from regional referral centers to 25-bed critical-access hospitals to ensure feedback on the questionnaire from a variety of perspectives. Based on cognitive debriefing of pilot testers with input from a nationally recognized expert in psychometrics and health outcomes measurement, the final survey document was created (Additional file 3).

### Survey sample

**Fig. 1:**
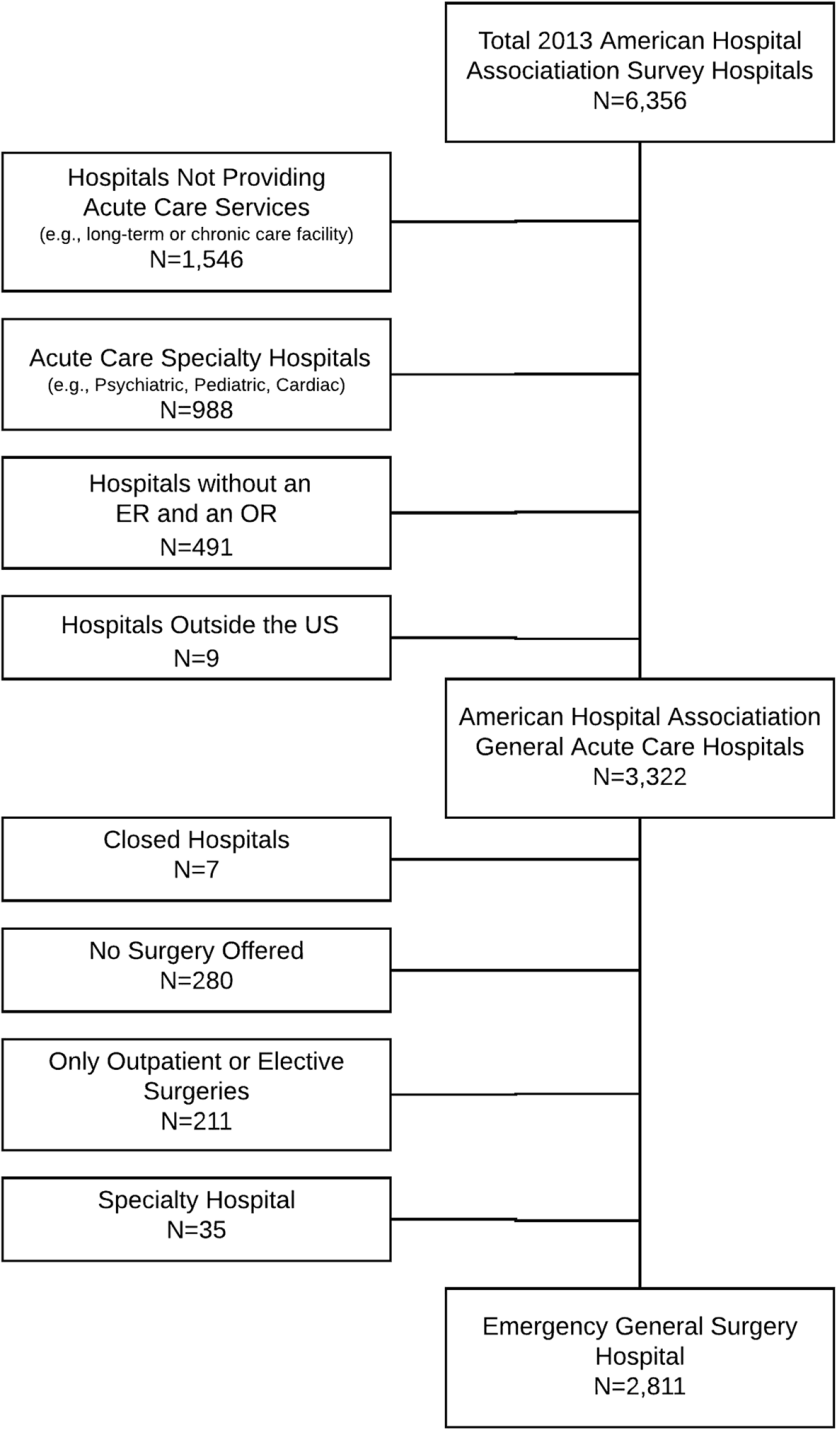
Flow Diagram for the Derivation of the Cohort of 2811 Acute Care General Hospitals in the US where an Adult with a General Surgery Emergency Might Receive Emergency General Surgery Care

For this nationally representative survey we sought to identify all hospitals in the US where an adult experiencing a general surgery emergency might receive care. We used the 2013 American Hospital Association (AHA) Annual Survey of Hospitals (*n* = 6356) to identify acute care general hospitals (non-federal, short-term general, and other special hospitals including academic medical centers or other teaching hospitals) accessible to the general public (i.e., not a Veterans Affairs Hospital, prison hospital, or college infirmary), known to treat patients age 18 and older that had an emergency department (ED) and at least one operating room (OR) [65]. From data reported to the AHA, we excluded (1) long-term or chronic care hospitals where emergency care is not provided (*n* = 1546); (2) acute care specialty hospitals (e.g., psychiatric, pediatric, cardiac) whose scope would not encompass general surgery or care of adults (*n* = 988); (3) hospitals lacking an ED and/or lacking at least one OR (*n* = 491); and (4) hospitals outside in US territories where health systems may not be similar to the 50 states (*n* = 9). We then confirmed the capacity to provide basic EGS care at the remaining 3322 hospitals using a grassroots approach of internet searches and direct phone calls to offices of chief medical officers (CMOs) and EDs. At the conclusion of this process, we excluded 511 hospitals (14%) that did not provide EGS care due to closure (*n* = 7), no surgical care offered (*n* = 280), only outpatient/elective surgery offered (*n* = 211), or misclassification in AHA data (*n* = 35). This left 2811 acute care general hospitals treating adults with the capacity to provide 24/7 access to EGS care for our survey sample. The hospital inclusion and exclusion criteria are shown in Fig. 1.

### Identifying survey respondents

**Fig. 2:**
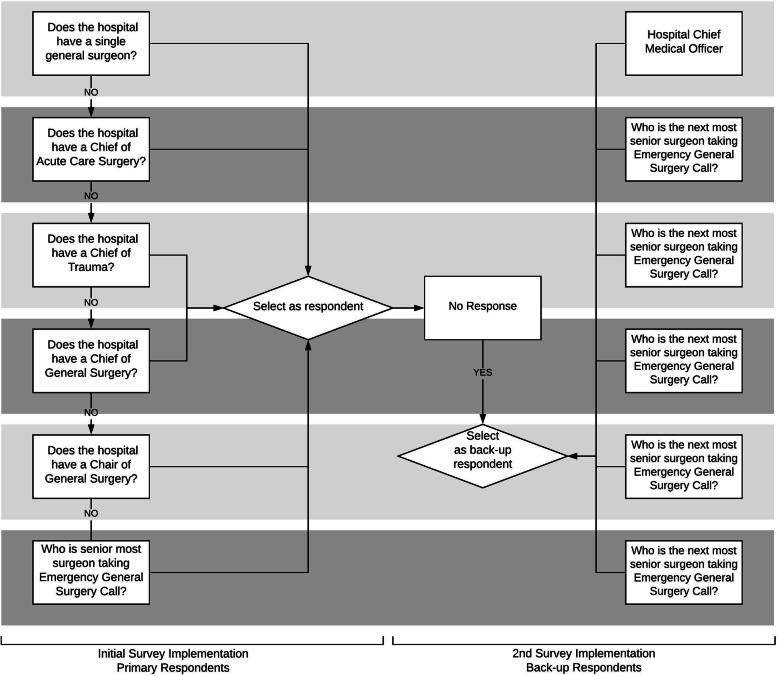
Algorithm for Selecting Survey Respondents to Measure Structures and Processes in Place for the Care of Emergency General Surgery Patients at US Hospitals

Our goal was to achieve a minimum 50% response rate. Due to the historically low response rate in physician survey research, [66, 67] we anticipated two rounds of survey implementation. We used the same grassroots approach as above to identify the surgeon at each hospital who would presumably be most knowledgeable regarding EGS structures and processes at the hospital, as well as backup respondents should the initial response rate not reach our target, using the algorithm shown in Fig. 2. All hospitals had at least one surgeon who met the respondent criteria for the first round of the survey implementation. At hospitals where we could only identify a single surgeon, we listed the hospital CMO as the backup respondent. Overall, 97.8% of potential respondents had valid postal mailing addresses, and 60.5% of potential respondents had valid email addresses.

### Survey implementation

We used the total design approach (TDA) [68, 69] to maximize the success of our survey implementation and address the inherent limitations of survey research [70–72]. TDA has yielded response rates as high as 68% and involves developing trust between surveyor and respondents, simplifying the burden of responding, and rewarding participation [68, 73]. We implemented TDA through a personalized letter (Additional file 4) from a colleagues with a shared concern regarding quality of EGS care (trust), both an online and paper-based response option (simplify response burden), and upfront inclusion of an incentive, a combination laser pointer pen and stylus (reward). We utilized an upfront incentive based on evidence that it is superior to post-payment or random lottery awards [74–76].

The first survey implementation from August 13 to October 26, 2015 resulted in a 41.8% response rate. A second round, from November 2 to December 22, 2015, using the same approach targeted secondary surgeons and CMOs at all hospitals without a response by November 1. The overall response rate was 60.1%. More than 95% of the hospitals (*n* = 1610) in our sample had data from surgeons while 4.7% (*n* = 80) had data from CMOs.

The characteristics of responding hospitals versus non-responding hospitals are shown in Table 1 using AHA variables. Respondent hospitals were primarily non-governmental (70.9% vs. 57.0%, *p* < 0.01), located in urban settings (62.3% vs. 59.9%, *p* = 0.19). A plurality of respondent hospitals also had fewer than 100 inpatient beds (37.8% vs. 47.0%, p < 0.01) while a minority were affiliated with medical schools (33.3% vs. 26.3%, p < 0.01).

**Table 1:** Characteristics of Non-Respondent versus Respondent Hospitals using 2015 American Hospital Association (AHA) Survey Results^a^

	Non-Respondent Hospitals (*N* = 1121)*	Respondent Hospitals (*N* = 1690)*	*p*-value
**Ownership Type N (%)**			**< 0.01**
Non-governmental	740 (66.0)	1181 (69.9)	
Governmental	186 (16.6)	307 (18.2)	
Investor-owned	182 (16.2)	189 (11.2)	
**Setting N (%)**			**0.40**
Urban	923 (82.3)	1417 (83.9)	
Rural	185 (16.6)	260 (15.4)	
**Teaching Status N (%)**			**< 0.01**
Major	63 (5.6)	161 (9.5)	
Minor	418 (37.3)	628 (37.2)	
Non-teaching	627 (55.9)	888 (52.5)	
**Inpatient Bed Capacity N (%)**			**0.15**
500+ beds	84 (7.5)	177 (10.5)	
400–499 beds	60 (5.4)	84 (5.0)	
300–399 beds	102 (9.1)	150 (8.9)	
200–299 beds	156 (13.9)	234 (13.9)	
< 200 beds	706 (63.0)	1032 (61.1)	
**US Census Regions and Divisions N (%)**			**< 0.01**
Midwest region			
*East North Central*	221 (19.7)	303 (17.9)	
*West North Central*	135 (12.0)	220 (13.0)	
Northeast region			
*Middle Atlantic*	98 (8.7)	192 (11.4)	
*New England*	44 (3.9)	90 (5.3)	
South region			
*South Atlantic*	172 (15.3)	273 (16.2)	
*East South Central*	91 (8.1)	123 (7.3)	
*West South Central*	184 (16.4)	196 (11.6)	
West region			
*Mountain*	74 (6.6)	131 (7.8)	
*Pacific*	89 (7.9)	149 (8.8)	
**Medical School Affiliation N (%)**			**0.03**
Yes	341 (30.4)	591 (35.0)	
No	767 (68.4)	1086 (64.3)	

^a^AHA characteristics are missing for 26 hospitals who answered the 2013 but not 2015 AHA survey

### Shaping survey domains into a Donabedian framework

The 16 page, 68 item questionnaire followed a logical, respondent-friendly flow of queries related to EGS care rather than queries grouped by similar structure and process domains. Therefore, to apply a Donabedian framework to our subsequent linked analyses, the survey items needed to be reorganized from a structure and process perspective. Therefore, a group of 10 individuals including the lead investigator, six additional surgeons across three hospitals, a biostatistician, an epidemiologist, and an implementation scientist reviewed the survey items and grouped them into domains conceptualized as structure, process, or a combination as detailed below. (Table 2) Ultimately, some survey items provided insight to more than one domain. The matrix in Additional file 5 shows how individual questions fit into various domain(s) for subsequent analyses.

**Table 2:** Components of Structure and Process Domains

Domain	Component
**Structure**	
EGS workforce	# of staff (nurse-practitioners, physician assistants, residents) caring for EGS patients
	# of EGS surgeons
	Demographic characteristics of EGS surgeons including sex and career stage (nearing retirement versus newly trained)
	Professional characteristics of EGS surgeons including subspecialty training, board certification, and other advanced degrees
	Frequency and timing of advanced practice practitioner, surgical resident, or medical student clinical assistance to EGS surgeons
	EGS surgeon clinical responsibilities in addition to EGS (e.g., trauma, critical care, burns, elective surgery, other)
	EGS surgeon employment models (e.g., academic, private practice)
	Financial incentives for EGS coverage including additional pay for overnight EGS coverage and compensation for encounters with uninsured patients while covering EGS
	EGS surgeon nonclinical responsibilities (e.g., surgical education, research, community outreach, administration)
Hospital staff	Round-the-clock availability of imaging technologists such as x-ray, ultrasound, and computed tomography technicians
	Round-the-clock availability of clinical laboratory and blood bank technicians
	Round-the-clock availability of respiratory therapists
	Overnight availability of perioperative staff including scrub technicians, OR nursing staff, recovery room nursing staff, and CRNAs
Subspecialty services	Anesthesiologists overnight availability
	Surgical pathologists overnight availability
	Round-the-clock availability of intensivists
	Method of ensuring round-the-clock intensivist coverage (e.g., tele-ICU, on-call in-house)
	Advanced endoscopists availability
	Interventional radiologists availability within one hour
**Process**	
Surgeon-patient contact	Processes to alert surgeons of an unstable EGS patient in the ER or after surgery
	How EGS patients are cohorted within patient censuses (e.g., only among other EGS patient, combined with trauma)
	Where EGS patients received care including on regular floors (e.g., dedicated EGS floor, med-surg) and the ICU (e.g., SICU, MICU, med-surg ICU)
	Where EGS patients typically receive care including inpatient (e.g., specifically assigned ward, floor with medical patients, surgical or medical ICU) and outpatient (e.g., dedicated EGS follow-up clinic)
	Overnight EGS surgeon in-house
	Daytime EGS surgeon coverage model (e.g., “on-service” or shift of defined length) including length of continuous EGS coverage
	Daytime surgeon or post-call surgeon freed from other responsibilities
	EGS surgeon salary incentives such as surgeon compensation for encounters with uninsured patients or for taking call
	Transfer agreements to send and/or receive EGS patients including approximate volume of transferred patients
Communication	Face-to-face hand-offs (e.g., timing, attendees, patients discussed)
	Alternatives to face-to-face hand-offs (e.g., telephone call, sending an email)
	Communication of critical results to surgeon by radiologist
Continuity of care	Likelihood of overnight surgeon providing EGS operation rounding on patient until discharge
	Likelihood of overnight surgeon providing EGS operation seeing that patient in follow-up clinic
	Likelihood of overnight surgeon providing EGS operation providing care if patient is readmitted
	Whether operating surgeon or surgical colleagues provide surgical critical care to EGS patients
	Presence of outpatient clinic specifically for EGS patients
	Frequency of transfer of EGS care (non-operative, post-operative, or post-discharge) to other clinicians including hospitalists, primary care physicians, and subspecialists
EGS team implementation	Overall EGS coverage model: dedicated team, tradition general surgeon on-call approach, other
	Daytime EGS surgeon coverage model (e.g., “on-service” or shift of defined length) including length of continuous EGS coverage
	Dedicated EGS team oversight (e.g., Division, Section), age (ie. date first implemented), and name/title of team
	EGS team composition by profession (e.g., surgeons, advanced practice practitioners) and stage of training (e.g., trainees, students, faculty)
	EGS coverage responsibilities (e.g., also covering trauma) or lack thereof (e.g., free of office responsibilities)
	Frequency and timing of advanced practice practitioner (NP, PA), surgical resident, medical student clinical assistance to EGS surgeons
Operating room access	# of operating rooms per the American Hospital Association survey
	Daytime “block” time (number of days) for EGS cases
	Tiered process for booking urgent or emergent cases
	Guidelines to defer elective operations for emergent cases
	EGS surgeon’s work schedule constraints including other clinical responsibilities, length of shifts, and post-call coverage
	Surgeons’ overnight coverage patterns including being in house, covering trauma, covering ICU, and covering at more than one hospital
	Overnight operating room availability
	Type and availability of overnight perioperative staff (e.g., scrub technicians, OR nursing staff, recovery room nursing staff)
Patient safety protocols	Activation system for unstable EGS patients in ER and guided response strategies to identify at risk EGS patients generally
	Guidelines to escalate care when patients deteriorate
	Protocols to address emergent patient care including anticoagulation reversal, massive transfusion, airway access, and emergent OR access
	Round-the-clock availability of physicians and specialized, rapid response teams to evaluate and manage deteriorating patients
	Processes for communicating critical patient information including radiographic findings and face-to-face signoffs
	Transfer agreements to send EGS patients to higher resourced hospitals
	Transfer agreements to facilitate round-the-clock critical care coverage
Performance improvement measures	Audits for return to OR during index hospitalization or within 30 days of discharge
	Audits for operation within 30 days of non-operative management of an EGS condition
	Audits for re-admission within 30 days after discharge
	Audits for return to ICU within 48 h of transfer to floor/ward
	Process to monitor time to initial evaluation after EGS consultation, time to OR after booking emergent case, and time to source control after determining EGS diagnosis
	Program managers for EGS patients with or without other responsibilities
	Prospective EGS registry
	Implementation of morbidity and mortality conference for EGS patients as dedicated M&M or integrated into existing M&M, including frequency and who attends
**Combined Elements: Structure and Process**	
Diagnostic radiology	Imaging technology available per the American Hospital Association survey
	Round-the-clock availability of imaging technologists such as x-ray, ultrasound, and computed tomography technicians
	Timeliness of study completion and read, including xray, ultrasound, and computed tomography
	Communication of critical results to surgeon by radiologist
	Use a tele-radiologist to read imaging studies overnight
	Interventional radiologists availability within one hour
Critical care resources	# of medical-surgical ICU beds per the American Hospital Association survey
	Availability critical care physicians and surgeons practicing critical care
	Who provides critical care for EGS patients (e.g., surgeon or pulmonary intensivist)
	Location where EGS patients receive care (e.g., SICU vs MICU)
	Round-the-clock availability of respiratory therapists
	Protocols in place to ensure urgent availability of blood products
	Protocols in place to identify post-op EGS patients requiring ICU admission
	Protocols to ensure rapid-response teams to provide airway access
	Protocols to ensure adherence to the Surviving Sepsis Campaign® guidelines
	Availability of round-the-clock physicians and specialized, rapid response teams to monitor unstable patients and establish airway access
	Round-the-clock availability of intensivists
	Method of ensuring round-the-clock intensivist coverage (e.g., tele-ICU, on-call in-house)
	EGS surgeons’ critical care credentials including board certification and additional fellowship training

### Data linkage

There are no existing mandates to prospectively collect data on EGS patients at the state or national level and it would have been cost prohibitive prospectively collect such data across 1690 hospitals. Therefore, despite the known limitations of administrative data, the only feasible way to measure the role of hospital-level EGS structures and processes on patient-level outcomes was to use existing data sources where episodes of EGS disease might be captured from compiled discharge billing and coding data. In order to ensure the anonymity of hospitals represented in our survey, a separate data sheet of survey identifiers and American Hospital Association Unique Identifiers (AHAID) was accessible only to the data analyst. AHAID was then used to link survey responses to existing administrative data collected in the same year as the survey data was collected.

### Choice of data sources

We considered several potential data sources (Table 3) before selecting the Agency for Healthcare Research and Quality (AHRQ) Healthcare Cost and Utilization Project (HCUP) State Inpatient Datasets (SIDs) and the Medicare Provider Analysis and Review (MedPAR) inpatient hospital claims data. Our main considerations were ability to anonymously link survey responses to the patient level data, generalizability, assuring that we were capturing data on EGS diseases which for the purposes of administrative data we assumed would be a diagnosis of interest and an associated hospitalization (i.e., we did not want to capture benign presentations of abdominal pain and soft tissue symptoms that resulting in a person being treated and released from the ED), and types of outcome data available. The ideal data source would provide ability to examine all-payer data on patients age 18 and older hospitalized for EGS diagnoses across all 50 states in 2015 with at least 90 days of post-discharge follow-up data on post-EGS outcomes.

**Table 3:** Comparison of Data Sources^a^Considered for this Project

	NIS	SID	MEDPAR	NEDS	APCD	NSQIP	UHC/Vizient
Chart Review (vs. administrative data)	No	No	No	No	No	Yes	No
Patient Tracking (vs. admission-level data)	No	No	Yes	No	Yes	No	No
100% Capture (vs. representative sampling)	No	Yes	Yes	No	Yes	No	Yes
Late Outcomes (vs. index admission only)	No	No	Yes^b^	No	Yes^b^	Yes^c^	No
Late Mortality (vs. early or index mortality)	No	No	Yes	No	Yes	No	No
Specific Risk Stratification (vs. generic)	No	No	No	No	No	Yes	Yes
Nationally Representative Sample (vs. biased)	Yes	No	Yes	Yes	No	No	No
Allows Study of Transferred Patients	No	No	No	No	No	Yes	Yes

^a^ NIS: Nationwide Inpatient Sample; MEDPAR: Medicare Provider Analysis and Review; *NEDS* Nationwide Emergency Room Sample, *APCD* All-Payer Claims Data, *SID* State Inpatient Databases, *NSQIP* National Surgical Quality Improvement Project, *UHC* University HealthSystems Consortium

^b^ longitudinal outcomes beyond 30 days

^c^ 30-day outcomes

SIDs contains data including all patients and all payers from approximately 97% of community hospital discharges submitted to HCUP from 49 participating states. HCUP releases the individual SID data sets in a format that allows comparisons between states [77]. In 2015, 17 states released data with AHAIDs available for data linkage. We considered HCUP’s National Inpatient Sample and the National Emergency Department Sample as alternatives to SIDs to obtain national data [78, 79]. Like SIDs, both also include demographics, diagnosis and procedure codes, comorbidities, AHRQ validated risk adjustment variables, complications, LOS, and charges [80]. However, NEDS lacked post-emergency room data and both NEDS and NIS lacked hospital identifiers precluding data linkage. In addition to possible lack of generalizability across all 50 states, a key limitation of SIDs was that outcomes data would be limited to index hospitalization only. Therefore, SIDs was the best available data for populations 18 and older regardless of insurance type who were treated across more than 500 various types of community hospitals in distributed across the US even if not all 50 states. To capture post-discharge outcomes in this 18 and older age group, we also considered the Vizient (formerly UHC) database used in our pilot work and The National Surgical Quality Improvement Program (NSQIP) as alternatives to SIDs because they include 30-day follow-up [63, 81]. Both also provide more robust risk stratification data. However, data from an alliance of academic medical centers and their affiliates or a voluntary, prospective, peer-controlled national quality collaborative that only captures data on patients undergoing operation, respectively, neither would be representative of general acute care hospitals in the US and the latter would not be applicable to the estimated 60–70% of EGS patients that do not require surgery.

MedPAR contains 100% capture of discharge data for all Medicare beneficiaries with the ability to track patients over time for post-discharge episodes of care [82]. MedPAR files are linked to chronic comorbidity files and other resource use files (i.e., durable medical equipment, skilled nursing facility) and therefore provide a longitudinal data source primarily for those 65 and older. Thus, including CMS data enriches the spectrum of analyses that are possible under our framework. Given eligibility requirements (chronic disability or end-stage renal disease) for Medicare coverage for the minority of beneficiaries younger than age 65, we opted to exclude this population from the present research in order to assure that our findings were generalizable to the typical older US population. We did consider state-level all-payer claims databases (APCDs) [83] as a resource for longitudinal data that would be representative of the entire adult (i.e., 18+) general hospital population as an alternative to MedPAR. APCDs would mirror MedPAR’s strengths with 100% percent capture across representative hospitals with longitudinal data across all age groups. For 2015, data are available for 19 states, however cost and substantial differences between states in terms of database structure precluded their use at the time of planning the present study.

Given that both MedPAR and SIDs had limitations with respect to age group, nationally representative data, and longitudinal outcomes, we believed that choosing only one would not allow us to fully examine the impact of the structures and processes measured in our survey. Due to the significant differences between data sets, however, we planned to separately link survey data to each data source. Disparate patient-level databases were not combined for outcomes analyses.

### Diseases of interest

In 2013 the American Association for the Surgery of Trauma expert panel recommended nomenclature for the scope of EGS diseases [22]. For the present research, we built upon this initial consensus statement using our pilot qualitative data and survey development research in combination with a review of all known single center reports of ACS implementation (at the time of designing this research) to develop a list of diseases most commonly urgently evaluated and treated by general surgeons on call and acute care surgeons. (Additional file 6) Many, but not all EGS diagnoses will require operation. Depending on our disease of interest and whether or not acuity of illness was relevant to our study questions, patient inclusion criteria might be based on ICD-9/10 diagnosis code alone, ICD-9/10 diagnosis code and operation at any point during hospitalization, or ICD-9/10 diagnosis code and operation during a specified time point as a proxy to acuity (i.e., urgency to intervention). For example, in already published or presented work from this research we have examined small bowel obstruction, gastrointestinal bleeding, and the entire list of EGS diseases in Additional file 6 simply based on diagnosis codes to study the efficacy of some EGS structures and processes [84–86]. Meanwhile we have examined presumed life-threatening EGS diseases based on both diagnosis codes *and* an operation on the date of admission to measure the role of OR access and Critical Care structures and processes on diseases whose standard of care is typically emergency operation [87, 88]. A list of operations consistent with treatments for our list of EGS diseases was created in a similar fashion. (Additional file 7).

### Outcomes of interest

Index hospitalization (SIDs, MedPAR) outcomes of interest included in-hospital mortality, LOS, systemic complications, surgical complications, and discharge disposition. In addition, MedPAR allows measurement of longitudinal outcomes including post-discharge mortality, re-admission, and late complications. In order to develop a comprehensive list of both systemic (could be experienced by all EGS patients) and surgical (experienced only by those undergoing operation) complications, we reviewed the existing reports on EGS diseases and reached consensus within our research team on the complications we would include as patient-level outcome measures. (Additional file 8).

### Statistical methods

Multivariable linear and logistic regression models constructed using a complete-case approach to explain and predict the relationship between clinical, demographic, and organizational factors and outcomes of interest for specific diseases or groups of diseases connected, for example, by organ system (e.g., biliary disease, appendicitis) or acuity (e.g., life-threatening EGS diseases) were chosen for analysis. Covariates include patient demographic characteristics and comorbidity index to adjust for potential confounding. Independent variables vary based on published reports on EGS disease, clinical judgment, and the results of univariate analyses. Multilevel models with dependent variables at patient- or hospital-level and predictor variables at both patient- and hospital-levels applying Generalized Linear Mixed Effect Models and Generalized Estimating Equations were chosen to allow for robust models and appropriate model testing LOS and other outcomes likely to be significantly skewed must be modeled using an appropriate distribution (i.e., gamma) based on published literature.

## Results

Below we will be presenting the results of our effort to organize survey questions into a set a domains consistent with the Donabedian Framework.

### Structural elements

Structure refers to the infrastructure and human capital elements of healthcare delivery, along with the organizational structure of staff and reimbursement models [89]. We therefore conceptualized structure as the resources that could influence EGS outcomes, considering three perspectives: EGS workforce, hospital staff, and access to subspecialty services. The ‘EGS workforce’ domain included human capital investments specific to the care of EGS patients, such as the number of EGS surgeons and advanced practice providers or trainees assisting in the care of EGS patients and demographic and professional characteristics of EGS surgeons. In addition, since salary incentives and professional opportunities also play an important role in retaining the emergency care workforce across specialties [90], employment models, salary incentives, and non-clinical responsibilities were included in the ‘EGS workforce’ domain. The ‘hospital staff’ domain encompassed hospital-level human capital whose round-the-clock (RTC) availability may impact EGS care given that EGS patients present at all times of the day. These staff positions include various technical-level personnel in imaging, laboratory testing, respiratory therapy, and OR and post-anesthesia nurses and staff. Finally, access to ‘subspecialty services’ that are routinely called upon for the care of EGS patients including anesthesiologists, intensivists, advanced endoscopists, and interventional radiologists was measured. Additional structural elements such as types of radiology equipment and number of ORs were derived from the AHA Annual Survey as we had intentionally sought in our survey to not duplicate queries already included in that data.

### Process elements

Processes refer to how healthcare services are provided and are comprised of events that occur during diagnosis and treatment [89]. Thus, we conceptualized processes as EGS-specific and other hospital-level processes that were important for care of EGS patients. To this end, we measured specific process domains within EGS care delivery including ‘surgeon-patient contact,’ ‘communication,’ ‘continuity of care,’ and ‘EGS team implementation,’ as well as broader hospital-level processes including ‘operating room access,’ ‘patient safety protocols,’ and ‘performance improvement measures’ that might also potentially improve outcomes even if not implemented specifically for EGS.

Within the ‘surgeon-patient contact’ domain, we ascertained ways in which direct access of EGS patients to surgeons is facilitated including processes to alert surgeons to the presence of an unstable EGS patient in the ED or post-operatively, cohorting of EGS patients on clinical censuses, location of EGS care within the hospital, and post-discharge EGS follow-up processes. This domain also included how EGS surgeon coverage is provided during day and night and whether the daytime EGS surgeon had other office or clinical responsibilities when attending on service or on their post-call day (i.e., after being on call overnight, for 24 h, or longer). Lastly, given that compensation might incentivize seeing patients [90], receiving compensation care of uninsured EGS patients or for providing EGS call coverage were included in the ‘surgeon-patient contact’ domain.

Face-to-face handoffs facilitate communication about key patient concerns, have been shown to improve quality of inpatient care, and have long been used by multidisciplinary trauma teams to facilitate daily handoffs in a 24/7 service line [61, 91–93]. Therefore, for the ‘communication’ domain, we included when and by whom face-to-face handoffs were used and their content. For those lacking a face-to-face handoff, the domain included how the information was shared (e.g., telephone, printed list, email). For the ‘continuity of care’ domain, we included survey items pertaining ongoing care of EGS patients after admission or operation such as who conducted daily rounds (i.e., admitting surgeon vs another assigned rounding colleague), provided outpatient follow-up after index hospitalization, or re-admitted patients for possible complications. Presence of a dedicated EGS follow-up clinic was also included in the ‘continuity of care’ domain.

For the ‘EGS team implementation’ domain we included whether EGS care was delivered by a dedicated clinical team that cares for EGS patients with or without other patients within the scope of trauma surgery or the newly proposed scope of acute care surgery (e.g., trauma, burns, and/or elective general surgery), a GSOC model, or another approach. For those with dedicated teams, the domain included how the team was housed (i.e., its own division, a division of general surgery or trauma/critical care) and how long the team had been in place. This domain also included team composition by profession and training stage, and whether surgeons covering EGS overnight provided in-house coverage, covered more than one hospital, or concurrently covered trauma or critical care. Finally, the ‘EGS team implementation’ domain also encompassed how often advance practice providers or trainees helped surgeons provide care EGS care during the day or night.

Timely ‘operating room access’ is essential to successful emergency surgery outcomes. Thus, this domain includes overall number of operating rooms, scheduling of emergency operations, availability of surgeons (including competing responsibilities), and the type and availability of overnight perioperative staff (e.g., anesthesiologists, certified nurse-anesthetists, nurses and technicians). ‘Patient safety protocols’ are hospital- or service line-level processes implemented to optimize outcomes. Given the acute nature of EGS disease and associated hemodynamic lability [94, 95], measures in this domain generally were considered as those intended to expedite appropriate care for patients in crisis including assorted activation systems (e.g., an emergency response team, code airway), protocols assuring rapid therapeutic interventions (e.g., urgently-needed anticoagulant reversal agents, massive transfusion protocol), availability of physicians and specialized teams, processes to defer elective cases or tier emergency cases, processes for communicating critical patient information (e.g., radiographic findings, patients deemed at risk for deterioration). In the ‘performance improvement’ domain, we included hospital-level or service line-level processes to monitor and address complications that disproportionately impact EGS patients, [24, 96] including audits of the time to initial surgical evaluation after consultation, source control (i.e., surgical removal of bowel perforation) after diagnosis, and start of an emergency operation, as well as audits for unplanned return to the OR, transfer to the ICU, and hospital readmission. Hospital- and service-line investments included the program managers to oversee quality and delivery of EGS care, prospective EGS patient registries, and morbidity and mortality conferences dedicated to EGS patients, all processes have been proven to be key to favorable outcomes among injured patients [97–106], were also include in the ‘performance improvement’ domain.

### Combined elements

In addition to their contribution individually, some structural and process elements are inextricably linked and can be viewed both as what tools or personnel are available (structure) and how they are used (process). Modern surgical decision-making has been heavily influenced by advances in diagnostic imaging techniques. Therefore, we constructed a hospital-wide ‘diagnostic radiology’ resources domain. Within this domain were the structural elements of the various kinds of radiographic technology available (e.g., computed tomography machine, ultrasound machines) and the presence of radiology workforce (e.g., technicians, radiologists). Processes included in this domain were the capability and time needed to obtain and read results for stat imaging requests, whether critical results were personally communicated by radiologists to surgeons, and use of a tele-radiologist to read imaging studies overnight. Further, as many as half of all EGS patients will require critical care [13, 18, 19, 23, 24]. Thus, ‘critical care resources’ must be evaluated in studying EGS outcomes. Structural components in this domain included the number and kinds of ICU beds and the critical care workforce (e.g., intensivists, respiratory therapists). Processes included in this domain included whether there were protocols to identify EGS patients requiring ICU admission, ensure 24/7 access to intensivist care, deploy rapid-response teams, and ensure adherence evidence based critical care guidelines [107, 108]. Finally, the ‘critical care resources’ domain also included who provided intensive care to critically-ill EGS patients (i.e., operating surgeon, a surgical colleague, or another intensivist). From the survey results alone, we have been able to provide evidence to describe the emerging paradigm of Acute Care Surgery and how such models of care differ from typical general surgeon on call models without a structured approach to EGS care [109–113].

## Discussion

This study was first proposed to the funding agency in 2013. After securing funding, we implemented a large national survey to examine hospital-level structures and processes that may impact EGS quality, designed the analytic approach using a Donabedian framework, and linked survey data to administrative data to measure patient-level outcomes. While our outcomes analyses are ongoing (and some have even been published) [84–88] the present manuscript is intended to provide the reader with great depth into our methodology because, like us, prior groups using similar methods have been limited in ability to share methodological insight presumably due to word count limitations in typical scientific manuscripts.

Using data on structures and processes gleaned from trauma center verification reviews, Moore and colleagues applied a Donabedian approach to trauma patients (~ 64,000 patients treated at 57 trauma centers) [114]. They identified correlation between a number of quality improvement measures on complications and length of stay [114]. Notably, no such verification process exists (yet) for EGS and we had to rely on a novel survey to gather rich data on EGS structures and processes, reaching nearly 1700 hospitals that have no state or national mandates to participate in quality improvement. Like us, Main and colleagues conducted a novel survey. They targeted 123 Veteran’s Administration Hospitals, of which 90 responded, with measures on the delivery of pre-operative, intra-operative, and post-operative care using 35 specific structure and process variables linked to aggregate morbidity and mortality data from a general surgery (elective and emergency) quality database requiring voluntarily provision of resources for quality improvement [115]. They found that 14 variables were associated with complications while only 4 were associated with 30-day mortality [115]. Their survey measures excluded a number of hospital level issues that might impact care unlike ours that took a broad view of structures and processes that might impact EGS patients either by design for an EGS service line or by happenstance due to structures and processes designed for the general patient population (i.e. all inpatients) or patients with other diagnoses (i.e. trauma patients). Ozdemir and colleagues published a study hospital-level structures and processes and the association with failure to rescue after EGS patients experienced complications in 156 British National Health System (NHS) Trust hospitals. Using administrative data routinely collected by the NHS and hospital-level data derived from a general NHS survey (similar to the AHA Annual Survey) rather than an EGS-focused one like ours, they found that lower surgeon staffing and lower nurse to patient ratios contributed to failure to rescue [116]. Our sample size, focus specifically on an EGS population, and specific survey data represent an opportunity to meaningful impact on the future structuring of EGS care delivery.

As with all scientific effort, the research methods described here are subject to potentially important limitations. First are limitations to the survey itself and hospital-level data which might be impacted by construct validity, social desirability bias and recall bias. We believe that we have limited these potential limitations through our iterative process of survey development and extensive pilot testing. That 40% of acute care general hospitals in the US with and ED, at least one OR, and some form of general surgery coverage were not represented in our sample creates possibility of lack of generalizability. This is further impacted in manuscripts using SIDs data limited to the responding hospitals in the 17 states that released AHAIDs to allow for data linkage. With that said, 60.1% represents an extraordinarily high response rate overall; furthermore, the 18.1% response rate when limited to just 17 states is on par with a number of published surgeon surveys including those by Johnson, Mehrzad, Carr, and Brahmandam who published results in 2018 and 2019 from surgeons surveys with response rates ranging from 15 to 18% [117–120]. In addition, as previously noted, at the patient-level SIDs lack of generalizability to EGS patients in all states while MedPAR lacks generalizability to the 50% of EGS patients who are not Medicare beneficiaries.

## Conclusion

Despite these limitations, the present manuscript provides a template for team science that is applicable to research efforts combining primary data collection (i.e., that derived from our survey) with existing national data sources (i.e., SIDs and MedPAR). The health services research methods described herein provide a foundation to further examine relationships between hospital-level structures and processes on EGS outcomes – a classic application of Donabedian framework. Results from studies previously published or to be published in the future based on our multimodal approach will enable us to develop a measure of preparedness for delivering EGS care in the US and provide key insights which hospital-level structures and processes affect EGS outcomes while accounting for unique patient risk factors and presenting EGS disease. Robust regionalized systems of care—with tiered hub-and-spoke models where “Centers of Excellence” at the hub require expert verification based on clear standards for structures and processes for care delivery including mandates for continuous quality improvement—that provide resources and guidelines for rapid evaluation and triage of patients with acute chest pain, neurologic deficits, and injury have been shown to reduce socioeconomic and geographic disparities in outcomes for myocardial infarction, cerebrovascular accident, and traumatic injury [31, 38, 39, 43, 121–127]. Currently, patients who experience acute-onset abdominal or skin/soft-tissue symptoms are at similar risk for morbidity and mortality but have access to no such resources. The body of work we intend to develop through the methodology described here will provide surgeons, health system leaders, and public health practitioners guidance on how to make such advances in EGS care delivery possible in the US.

## Supplementary information

**Additional file 1.** Interview Template for 18 semi-structured interviews conducted to inform survey development.

**Additional file 2.** Questionnaire from Pilot Survey of University Health Systems Consortium (now Vizient) Hospitals.

**Additional file 3.** Questionnaire from National Survey of 2811 Acute Care General Hospitals in the US where an Adult with a General Surgery Emergency Might Receive Emergency General Surgery Care.

**Additional file 4.** Cover Letter Included with National Survey.

**Additional file 5.** Matrixed Table of Survey Question Alignment with Structure and Process Domains.

**Additional file 6.** ICD-9 and ICD-10 Diagnosis Codes Used to Identify Emergency General Surgery Cases.

**Additional file 7.** ICD-9 and ICD-10 Diagnoses and Procedure Codes Used to Identify Complications.

**Additional file 8.** ICD-9 and ICD-10 Procedure Codes for Emergency General Surgery Cases.

## Data Availability

The datasets used and/or analysed during the current study available from the corresponding author on reasonable request.

## Abbreviations

ACS: Acute Care Surgery
EGS: Emergency General Surgery
AHA: American Hospital Association
MedPAR: Medicare Provider Analysis and Review
SIDs: State inpatient databases
IOM: Institute of Medicine
US: United States
TQIP: Trauma quality improvement program
LOS: Length of stay
UHC: University Health Systems Consortium
GSOC: General Surgeon On Call
ED: Emergency Department
OR: Operating room
CMO: Chief Medical Officers
TDA: Total design approach
RTC: Round-the-clock
ICU: Intensive Care Unit
AHAID: American Hospital Association Unique Identifiers
AHRQ: Agency for Healthcare Research and Quality
HCUP: Healthcare Cost and Utilization Project
NEDS: Nationwide Emergency Department Sample
NIS: National (Nationwide) Inpatient Sample
NSQIP: National Surgical Quality Improvement Program
CMS: Centers for Medicare & Medicaid Services
APCDs: All-payer claims databases
SAS: Statistical analysis system
