# Supplementary material for: Identifying the fundamental structures and processes of care contributing to emergency general surgery quality using a mixed-methods Donabedian approach
Source: BMC Med Res Methodol. 2020 Oct 2;20:247. doi: 10.1186/s12874-020-01096-7 (PMC7532630; doi:10.1186/s12874-020-01096-7)
Supplement: Supplementary file 1 — Additional file 1. Interview Template for 18 semi-structured interviews conducted to inform survey development. [file 12874_2020_1096_MOESM1_ESM.pdf]

Respondent : **Date:**

Acute Care Surgery Specialty

1. Do you consider Acute Care Surgery a specialty within surgery?

If not, ask the following:

1a. How do you consider it?

2. How do you define Acute Care Surgery as a surgical specialty [or whatever used above]?

3. Describe the evolution of Acute Care Surgery as a specialty [or whatever used above].

Acute Care Surgery Team

4. How long has your institution had an Acute Care Surgery team?

5. What clinical problems does your Acute Care Surgery team provide care for?

If trauma and non-trauma surgical emergencies are grouped into a single team ask the following:

5a-combined. What is the rationale for a combined trauma and emergency surgery team?

If trauma is a separate team from the team for non-trauma surgical emergencies ask:

5a-separate. What is the rationale for separate teams of trauma and non-trauma surgical emergencies?

Also ask the following:

5b. What is your institution's approximate volume of trauma cases and non-trauma surgical emergencies annually using 2010 as a reference point?

6. Describe how your institution's Acute Care Surgery Team is structured?

If not answered above ask the following:

6a. Who makes up the team?

6b. What are their qualifications/credentials?

6c. How many such individuals are there on the team?

6d. What other responsibilities do they have?

6e. Describe how residents function on the team.

7. How is call structured? (ask for copy of last 3 month call schedule as an example)

Acute Care Surgery Infrastructure

8. What are your institutional resources for caring for Acute Care Surgery patients?

If not answered above ask the following:

8a. What is your operating room availability for non-traumatic surgical emergencies?

8b. What is your surgical ICU capacity?

8c. Describe your ancillary and subspecialty support?

9. Is your institution a designated level 1 trauma center?

If yes, ask the following:

9a-yes. How, if at all, do you leverage resources from the trauma center infrastructure for Acute Care Surgery?

If no, ask the following:

9a-no. If you had a Level I trauma center, how would you imagine leveraging resources from the trauma center infrastructure for Acute Care Surgery?

10. Do you collect data for your Acute Care Surgery patients? If so, how and why?

Acute Care Surgery Model

Okay, now that you've described your Acute Care Surgery model, consisting of the team and the institutional resources…

11. How do you facilitate communication in this model, both within the team and between the team and its partners across the institution?

12. What benefits does your Acute Care Surgery model provide at the departmental level, at the institutional level and to the broader community that you serve?

If not answered above ask the following:

12a. Approximately what proportion of your Acute Care Surgery patients are referred from outlying hospitals?

13. What do you think are the strengths and weaknesses of your Acute Care Surgery model?

14. Do you think that the Acute Care Surgery model is financially viable? How so?

Acute Care Surgery Generalizations

15. Why do you practice Acute Care Surgery?

16. What kind of training should residents who also hope to practice Acute Care Surgery have?

If not answered above ask the following:

16a. Do you believe that Acute Care surgeons need specialized fellowship training?

17. If you could have unlimited resources for an ideal Acute Care Surgery model, how would you design it?

18. What do you think the future holds for Acute Care Surgery as a specialty [or whatever used above]?
