# Supplementary material for: Identifying the fundamental structures and processes of care contributing to emergency general surgery quality using a mixed-methods Donabedian approach
Source: BMC Med Res Methodol. 2020 Oct 2;20:247. doi: 10.1186/s12874-020-01096-7 (PMC7532630; doi:10.1186/s12874-020-01096-7)
Supplement: Supplementary file 2 — Additional file 2. Questionnaire from Pilot Survey of University Health Systems Consortium (now Vizient) Hospitals. [file 12874_2020_1096_MOESM2_ESM.pdf]

Emergency General Surgery Survey

Thank you for agreeing to participate in this survey.
The questions that follow will solicit your general opinions or ask you to reflect on the current structures and processes in place at your hospital. If you provide care at more than one clinical site, your responses should reflect the hospital where you provide the most coverage for emergency general surgery. The survey should take less than ten minutes to complete.

Emergency General Surgery Survey

Please indicate the degree to which you agree or disagree with the following statements about emergency general surgery.

There is a national crisis in availability of surgeons for general surgery emergencies.

- ☐ strongly disagree
- ☐ disagree
- ☐ neutral
- ☐ agree
- ☐ strongly agree

At my hospital it is difficult to find surgeons to take call for general surgery emergencies.

- ☐ strongly disagree
- ☐ disagree
- ☐ neutral
- ☐ agree
- ☐ strongly agree

Care of general surgery emergencies should be regionalized.

- ☐ strongly disagree
- ☐ disagree
- ☐ neutral
- ☐ agree
- ☐ strongly agree

Care of general surgery emergencies should be combined with trauma and critical care.

- ☐ strongly disagree
- ☐ disagree
- ☐ neutral
- ☐ agree
- ☐ strongly agree

Current graduating general surgery residents who will be caring for general surgery emergencies should pursue fellowship training that includes emergency general surgery.

- ☐ strongly disagree
- ☐ disagree
- ☐ neutral
- ☐ agree
- ☐ strongly agree

Please proceed to page 2

For each of the following clinical scenarios of patients who present to your hospital's emergency department, please indicate who would typically be called first by the ED physician to evaluate the patient.

51 year old morbidly obese diabetic male with Fournier's gangrene

- ☐ general surgeon on call
- ☐ urologic surgeon on call
- ☐ surgeon staffing designated emergency general surgery service
- ☐ other (specify) __________

37 year old female three years s/p Roux-en-Y gastric bypass for morbid obesity with internal hernia

- ☐ general surgeon on call
- ☐ bariatric surgeon on call
- ☐ surgeon staffing designated emergency general surgery service
- ☐ other (specify) __________

71 year old male with esophageal perforation due to Boerhaave's Syndrome

- ☐ general surgeon on call
- ☐ thoracic surgeon on call
- ☐ surgeon staffing designated emergency general surgery service
- ☐ other (specify) __________

51 year old male with peritonitis after index screening colonoscopy in your hospital's endoscopy suite earlier that day

- ☐ general surgeon on call
- ☐ colorectal surgeon on call
- ☐ surgeon staffing designated emergency general surgery service
- ☐ other (specify) __________

23 year old IV drug user with necrotizing soft tissue infection of her right arm

- ☐ general surgeon on call
- ☐ plastic surgeon on call
- ☐ surgeon staffing designated emergency general surgery service
- ☐ other (specify) __________

53 year old female 2 weeks s/p abdominal hysterectomy with small bowel obstruction

- ☐ general surgeon on call
- ☐ ob/gyn surgeon on call
- ☐ surgeon staffing designated emergency general surgery service
- ☐ other (specify) __________

Please proceed to page 3

For the questions that follow, please check the response that best describes your hospital's characteristics.

Location

- ☐ University Hospital
- ☐ Community Hospital
- ☐ State/County/City Public Hospital
- ☐ Veterans' Affairs Hospital
- ☐ Other (describe: ______________________________)

Setting

- ☐ Urban
- ☐ Suburban
- ☐ Rural

Teaching Status

- ☐ Teaching
- ☐ Non-teaching

Trauma Center Verification

- ☐ Level 1
- ☐ Level 2
- ☐ Level 3
- ☐ Not a designated trauma center

Inpatient Bed Capacity

- ☐ ≤100
- ☐ 101-200
- ☐ 201-300
- ☐ 301-400
- ☐ 401-500
- ☐ ≥501

Please proceed to page 4

For the questions that follow, please check the response that best describes your hospital's infrastructure for general surgery emergencies.

What is your hospital's designated operating room block time for general surgery emergencies?

- ☐ None
- ☐ One day per week
- ☐ Two days per week
- ☐ Three days per week
- ☐ Four days per week
- ☐ Five days per week
- ☐ More than 5 days per week

In general, where do your hospital's critically ill patients with general surgery emergencies receive intensive care?

- ☐ Surgical ICU
- ☐ MedSurg ICU
- ☐ Trauma ICU
- ☐ Other (specify) ____________________

What proportion of your hospital's general surgery emergency patients are transferred from another hospital?

- ☐ <1%
- ☐ 1-20%
- ☐ 21-40%
- ☐ 41-60%
- ☐ 61-80%
- ☐ >80%

Does your hospital maintain a prospective registry of general surgery emergency patients?

- ☐ Yes
- ☐ No

Please proceed to page 5

*Please check the response below that **BEST** describes your hospital's overall strategy for emergency general surgery coverage.*

☐ Surgeons assigned to the daily '**general surgeon on call**' schedule to provide coverage for general surgery emergencies ➡ *If you checked '**general surgeon on call**,' please proceed to page 6*

☐ There is a '**designated clinical service**' that provides 24/7 coverage for general surgery emergencies (+/- trauma) ➡ *If you checked '**designated clinical service**,' please skip to page 7*

☐ '**Hybrid**' coverage shared between a 'designated clinical service' and assigned 'general surgeon on call' responsibilities ⬇ *If you checked '**hybrid**,' please answer the question below*

What is the approximate division of emergency general surgery coverage between the 'designated clinical service' and surgeons assigned to 'general surgeon on call' responsibilities?

	'Designated Clinical Service'	'General Surgeon on Call'
☐	<25%	>75%
☐	~25%	~75%
☐	~50%	~50%
☐	~75%	~25%
☐	>75%	<25%

➡ *Please proceed to pages 6-8 to describe your '**hybrid**' model*

☐ **Other** (please describe below)

➡ *Thank you for participating in the survey. Proceed to the back cover.*

General Surgeon on Call Description

*(only complete this page if you checked '**general surgeon on call**' or '**hybrid**' on page 5)*

How many surgeons participate in this 'general surgeon on call' pool? ☐

In general, how many nights per month would one of these surgeons take overnight call for emergency general surgery? ☐

Do these surgeons take in-house call for emergency general surgery?
☐ Yes
☐ No

Do these surgeons earn any additional compensation or a stipend for taking emergency general surgery call?

- ☐ Yes, for each assigned call → Specify amount of compensation per call: $ ______
- ☐ Yes, for any call beyond a certain threshold → Specify number of calls before extra compensation: ______ → Specify amount of compensation per extra call: $ ______
- ☐ No, there is no additional call stipend

In general, are these surgeons freed of patient care responsibilities on the day following call?
☐ Yes
☐ No

Do any of the surgeons in this call pool have subspecialty training beyond general surgery residency?
☐ Yes
☐ No

If yes, please indicate which subspecialty training is represented in this call pool. (Check all that apply and provide the number of surgeons with each kind of training.)

- ☐ Minimally invasive surgery (N = ______)
- ☐ Surgical oncology (N = ______)
- ☐ Colorectal surgery (N = ______)
- ☐ Breast surgery (N = ______)
- ☐ Endocrine surgery (N = ______)
- ☐ Surgical critical care (N = ______)
- ☐ Other (specify ______________) (N = ______)
- ☐ Other (specify ______________) (N = ______)

*If you checked '**general surgeon on call**' on the previous page, you are now done with the survey. Please proceed to the back cover.*

*If you checked '**hybrid**', please proceed to page 7.*

Designated Clinical Service Description: Part 1

*(only complete this page if you checked '**designated clinical service**' or '**hybrid**' on page 5)*

How many surgeons staff your designated service for emergency general surgery? ☐

Do any of the surgeons on this service have subspecialty training beyond general surgery residency?
☐ Yes
☐ No

If yes, please indicate which subspecialty training is represented in this service. (Check all that apply and provide the number of surgeons with each kind of training.)

- ☐ Minimally invasive surgery (N = ______)
- ☐ Surgical oncology (N = ______)
- ☐ Colorectal surgery (N = ______)
- ☐ Breast surgery (N = ______)
- ☐ Endocrine surgery (N = ______)
- ☐ Trauma surgery (N = ______)
- ☐ Acute care surgery (N = ______)
- ☐ Surgical critical care (N = ______)
- ☐ Other (specify ______________) (N = ______)

What other clinical responsibilities do full-time surgeons on this service have? (check all that apply)

- ☐ None (only covers emergency general surgery)
- ☐ Trauma
- ☐ Elective general surgery
- ☐ Surgical critical care
- ☐ Other (specify) ______________

What non-clinical responsibilities do full-time surgeons on this service have? (check all that apply)

- ☐ None (performs only clinical duties)
- ☐ Research
- ☐ Education
- ☐ Administration
- ☐ Other (specify) ______________

In general, how many nights per month would a full-time surgeon on this service take overnight call? ☐

Do these surgeons take in-house call?
☐ Yes
☐ No

Do these surgeons earn any additional compensation or a stipend for taking call?

- ☐ Yes, for each assigned call → Specify amount of compensation per call: $ ______
- ☐ Yes, for any call beyond a certain threshold → Specify number of calls before extra compensation: ______ → Specify amount of compensation per extra call: $ ______
- ☐ No, there is no additional call stipend

In general, are these surgeons freed of patient care responsibilities on the day following call?
☐ Yes
☐ No

Please proceed to page 8

Designated Clinical Service Description: Part 2

*(only complete this page if you checked '**designated clinical service**' or '**hybrid**' on page 5)*

Which of the following BEST describes how the service organizes patient care?

- ☐ Emergency general surgery patients are on their own census.
- ☐ Emergency general surgery patients are on a combined census with trauma patients.
- ☐ Emergency general surgery patients are on a combined census with elective general surgery patients.
- ☐ Emergency general surgery patients, elective general surgery patients, and trauma patients are on a combined census.
- ☐ Other (specify) ______________________________

In general, does your service transfer care of patients to subspecialty services when their emergency has been addressed?

- ☐ Yes
- ☐ No

In general, who makes up the team in addition to the surgeons described above? (check all that apply and provide the number of team members for each category)

- ☐ Physician Extenders (eg. NP, PA) (N = ______)
- ☐ Surgical Chief Resident (Clinical PGY-5) (N = ______)
- ☐ Surgical Senior Resident (Clinical PGY-4) (N = ______)
- ☐ Surgical Midlevel Resident (Clinical PGY-3) (N = ______)
- ☐ Surgical Junior Resident (Clinical PGY-2) (N = ______)
- ☐ Surgical Intern (Clinical PGY-1) (N = ______)
- ☐ Other (specify ____________________) (N = ______)
- ☐ Other (specify ____________________) (N = ______)

Does the service conduct face-to-face patient hand-offs (eg. morning report, sign out rounds)?

- ☐ Yes
- ☐ No

If yes, who is typically required to attend the hand-off meeting? (check all that apply)

- ☐ Overnight residents
- ☐ Overnight attending
- ☐ Off-service attendings
- ☐ Other (specify ____________________)
- ☐ Daytime residents
- ☐ Daytime attending
- ☐ Physician Extenders (eg. NP, PA)
- ☐ Other (specify ____________________)

You are now done with the survey.
Please proceed to the back cover.

Thank you for completing the survey.

☐ Please check this box if you **do not** wish to receive summarized survey results at the conclusion of this study.

If you would like a summary of our survey results sent to you at the completion of this study, please enter your email below. **Your email will not be used or distributed for any other purpose.**

email ______________________________

If you have any comments or would like to share other thoughts on emergency general surgery with us, please use the space below:

We can be contacted at **egs-survey@umassmed.edu** if you have any additional questions or concerns.
