# Supplementary material for: Identifying the fundamental structures and processes of care contributing to emergency general surgery quality using a mixed-methods Donabedian approach
Source: BMC Med Res Methodol. 2020 Oct 2;20:247. doi: 10.1186/s12874-020-01096-7 (PMC7532630; doi:10.1186/s12874-020-01096-7)
Supplement: Supplementary file 3 — Additional file 3. Questionnaire from National Survey of 2811 Acute Care General Hospitals in the US where an Adult with a General Surgery Emergency Might Receive Emergency General Surgery Care. [file 12874_2020_1096_MOESM3_ESM.pdf]

Emergency General Surgery Survey

*Thank you for agreeing to participate in this survey on emergency general surgery structures and processes. Your responses should represent what is **currently implemented** at the **hospital indicated below**. Survey data will be analyzed in aggregate form without identifying you or your hospital.*

«Hospital_Name»

«Hospital_City», «Hospital_State»

Supported by a grant from the Agency for Healthcare Research Quality
Health Systems Research Section
(Grant number R01HS22694)

Hospital-wide Structures and Processes

*Questions 1-13 pertain to **overall structures and processes** of care at your hospital.*

1. Does your hospital provide **round-the-clock** (24/7/365) availability of **critical care specialists** (i.e., physicians with fellowship training and board certification in pulmonary critical care, anesthesia critical care, or surgical critical care)?

☐1 Yes
☐2 No. . . . . **If *No*, go to #3**

2. How does your hospital assure round-the-clock access to **critical care specialists**? *(Select one response only)*

☐1 In-house critical care specialist at all times (24/7/365)
☐2 Critical care specialist on-call when a critical care specialist is NOT available in-house
☐3 Tele-ICU service when a critical care specialist is NOT available in-house
☐4 Transfer agreement(s) to facilitate access to critical care when not available at my hospital
☐5 Other (please specify) ____

3. What is your hospital's availability of **ERCP** for biliary emergencies? *(Select one response only)*

☐1 An endoscopist who can perform ERCP available/on-call at all times (24/7/365)
☐2 An endoscopist who can perform ERCP available during weekdays only
☐3 No availability of ERCP at my hospital
☐4 Other (please specify) ____

4. Does your hospital have **overnight operating room availability**?

☐1 Yes
☐2 No. . . . . **If *No*, go to #6 on Page 2**

5. Please describe your hospital's **overnight operating room staff** presence. *(Select a response for each row)*

	In-house	Can be called in	Not Available	Unsure
a. Scrub technician(s)	☐1	☐2	☐3	☐4
b. OR nursing staff	☐1	☐2	☐3	☐4
c. PACU/recovery room nursing staff	☐1	☐2	☐3	☐4
d. Anesthesiologists (MD, DO, MBBS)	☐1	☐2	☐3	☐4
e. CRNA(s)	☐1	☐2	☐3	☐4
f. Surgical pathologist(s) for frozen section	☐1	☐2	☐3	☐4

6. Does your hospital provide **round-the-clock** (24/7/365) **in-house** presence of the following **ancillary staff**? *(Select a response for each row)*

	Yes	No	Unsure
a. X-ray technician(s)	☐1	☐2	☐3
b. Ultrasound technician(s)	☐1	☐2	☐3
c. CT technician(s)	☐1	☐2	☐3
d. Respiratory therapist(s)	☐1	☐2	☐3
e. Clinical laboratory technician(s)	☐1	☐2	☐3
f. Blood bank technician(s)	☐1	☐2	☐3

7. How frequently would the following occur for **radiographic studies ordered "STAT"** from your hospital's radiology department? *(Select a response for each row)*

	Always	Often	Sometimes	Rarely	Never
a. A CT scan is completed within 4 hours of placing the order	☐1	☐2	☐3	☐4	☐5
b. An ultrasound is completed within 4 hours of placing the order	☐1	☐2	☐3	☐4	☐5
c. A board certified/board eligible radiologist reads the study within two hours of completion	☐1	☐2	☐3	☐4	☐5
d. A study completed overnight is read by a tele-radiologist	☐1	☐2	☐3	☐4	☐5
e. Critical study findings are personally communicated (e.g., by telephone, face-to-face) by the radiologist to the ordering physician	☐1	☐2	☐3	☐4	☐5
f. The interventional radiology team is available in-house within 1 hour of requesting the intervention (e.g., angioembolization)	☐1	☐2	☐3	☐4	☐5

8. Does your hospital have the following established for patients who may clinically deteriorate? *(Select a response for each row)*

	Yes	No	Unsure
a. A graded response strategy (e.g., a modified early warning score, rapid response team) to identify patients at risk of clinical deterioration	☐1	☐2	☐3
b. Guidelines directing escalation of care when patients clinically deteriorate	☐1	☐2	☐3
c. Round-the-clock, in-house availability of a board certified/board eligible physician to evaluate deteriorating patients	☐1	☐2	☐3

9. Does your hospital have protocols in place to ensure the following? *(Select a response for each row)*

	Yes	No	Unsure
a. Urgent availability of blood products (e.g., massive transfusion protocol)	☐1	☐2	☐3
b. Response of qualified individuals (i.e., anesthesia, emergency medicine, surgery, and/or critical care) to establish airway access for a patient in respiratory distress (e.g., code airway team)	☐1	☐2	☐3
c. Risk factor based DVT/PE prevention (e.g., thrombosis risk assessment tool)	☐1	☐2	☐3

10. Does your hospital have the following established for urgent or emergent operations? *(Select a response for each row)*

	Yes	No	Unsure
a. A tiered system to ensure that urgent/emergent operations are started in a timely manner	☐1	☐2	☐3
b. Guidelines for deferral of elective operations to give adequate priority to urgent/emergent operations	☐1	☐2	☐3
c. A protocol for urgent/emergent reversal of common anticoagulant drugs	☐1	☐2	☐3
d. A process to ensure that patients found to have cancer receive oncologic follow-up according to National Comprehensive Cancer Network guidelines	☐1	☐2	☐3
e. A process to ensure that patients in septic shock receive care according to the Surviving Sepsis Campaign guidelines	☐1	☐2	☐3

11. Does your hospital ever lack round-the-clock (24/7/365) emergency general surgery coverage?

☐1 Yes

☐2 No. . . . . **If *No*, go to #14 on Page 4**

12. Approximately how often does your hospital lack emergency general surgery coverage? (*Please provide your best estimate in the space below)*

_________%

13. How frequent are the following reasons for lacking coverage? *(Select a response for each row)*

	Always	Often	Sometimes	Rarely	Never
a. Lack of general surgery coverage	☐1	☐2	☐3	☐4	☐5
b. Lack of anesthesia coverage	☐1	☐2	☐3	☐4	☐5
c. Lack of OR staff	☐1	☐2	☐3	☐4	☐5
d. Emergency room is on diversion	☐1	☐2	☐3	☐4	☐5
e. Other (please specify) ______________________	☐1	☐2	☐3	☐4	☐5

Emergency General Surgery Workforce

*The following questions pertain to the surgeons who cover **new** emergency department or inpatient consults for **patients** with suspected general surgery emergencies (e.g., appendicitis, perforated viscus, necrotizing fasciitis), sometimes referred to as "unassigned patients." Do not include surgeons who provide emergency coverage for their own, or their partners', existing patients in your responses.*

14. How many surgeons participate in emergency general surgery coverage at your hospital?

________ surgeons

15. Of the surgeons who participate in emergency general surgery coverage at your hospital, how many fall into the following categories?

a. Female surgeons ________

b. Surgeons over 65 years of age ________

c. Surgeons who finished training within the last 3 years ________

16. What is the **primary employment model** of the surgeons who cover emergency general surgery at your hospital? *(Select all that apply and indicate the number of surgeons for each model)*

☐1 Hospital Employed (N = ________)
☐2 Academic/University Practice (N = ________)
☐3 Private Practice (N = ________)
☐4 City/County/Federal Government Employed (N = ________)
☐5 Locum Tenens (N = ________)
☐6 Other (please specify) __________________________________ (N = ________)

17. Does the clinical practice of these surgeons encompass **other types of surgical care** (e.g., trauma, elective general surgery) in addition to emergency general surgery (not necessarily simultaneously)?

☐1 Yes
☐2 No. . . . . **If *No*, go to #19 on Page 5**

18. What **other types of surgical care** do they provide? *(Select all that apply and indicate the number of surgeons for each type of surgical care)*

☐1 Trauma (N = ________)
☐2 Burns (N = ________)
☐3 Surgical Critical Care (N = ________)
☐4 Elective General Surgery (N = ________)
☐5 Other (please specify) __________________________________ (N = ________)

19. Do these surgeons have **non-clinical roles** (e.g., research, administration) in addition to their clinical roles (not necessarily simultaneously)?

☐1 Yes
☐2 No. . . . . **If *No*, go to #22**

20. What non-clinical roles do they have? *(Select all that apply and indicate the number of surgeons for each non-clinical role)*

☐1 Surgical Education (e.g., program director; curriculum development) (N = ________)
☐2 Research (e.g., basic science research; clinical trials; outcomes research) (N = ________)
☐3 Community Outreach/Public Health (e.g., EMS lectures; international work) (N = ________)
☐4 Administration (e.g., chief medical officer; head of practice plan; chairperson) (N = ________)
☐5 Other (please specify) ____ (N = ________)

21. Do any of these surgeons specifically conduct **emergency general surgery research**?

☐1 Yes
☐2 No

22. Do any of these surgeons have **additional subspecialty training** beyond general surgery?

☐1 Yes
☐2 No. . . . . **If *No*, go to #24 on Page 6**

23. What additional subspecialty training do they have? *(Select all that apply and indicate the number of surgeons with each subspecialty training)*

☐1 Acute Care Surgery (N = ________)
☐2 Burn Surgery (N = ________)
☐3 Surgical Critical Care (N = ________)
☐4 Trauma Surgery (N = ________)
☐5 Breast Surgery (N = ________)
☐6 Colorectal Surgery (N = ________)
☐7 Endocrine Surgery (N = ________)
☐8 Hepatobiliary Surgery (N = ________)
☐9 Minimally Invasive Surgery (N = ________)
☐10 Thoracic Surgery (N = ________)
☐11 Surgical Oncology (N = ________)
☐12 Vascular Surgery (N = ________)
☐13 Other (please specify) ____________________________________ (N = ________)

24. What is the **board certification** status of the surgeons who cover emergency general surgery at your hospital? *(Select all that apply and indicate the number of surgeons with each certificate)*

- ☐1 American Board of Surgery Certified/Eligible in Surgery (N = ________)
- ☐2 American Osteopathic Board of Surgery Certified/Eligible in Surgery (N = ________)
- ☐3 American Board of Surgery Certified/Eligible in Surgical Critical Care (N = ________)
- ☐4 Other (please specify) ____ (N = ________)

25. Do any of the surgeons who cover emergency general surgery have **additional degrees** beyond their medical degree (e.g., MD, DO, MBBS)?

- ☐1 Yes
- ☐2 No. . . . . **If *No*, go to #27**

26. What additional degrees do they have? *(Select all that apply and indicate the number of surgeons with each degree)*

- ☐1 Masters of Public Health, MPH (N = ________)
- ☐2 Masters of Business Administration, MBA (N = ________)
- ☐3 Masters of Healthcare Administration, MHA (N = ________)
- ☐4 Masters of Education, MSEd (N = ________)
- ☐5 Masters of Science, MS (N = ________)
- ☐6 Doctorate, PhD (N = ________)
- ☐7 Other (please specify) ____________________________________ (N = ________)

Emergency General Surgery Coverage

The following questions pertain to how surgeons cover emergency general surgery at your hospital.

27. Does the surgeon covering emergency general surgery receive compensation for **uninsured patient encounters** resulting from emergency general surgery coverage?

- ☐1 Yes
- ☐2 No

28. Which of the following statements *BEST* describes how your hospital designates surgeons for **daytime** emergency general surgery coverage? *(Select one response only)*

The surgeon covering emergency general surgery during the daytime is…

- ☐1 "On-service" (i.e., covering emergency general surgery for 2 or more consecutive days)
- ☐2 Assigned to a daytime shift (e.g., 8, 12, 14 hrs long). . . . . **Go to #30 on Page 7**
- ☐3 Assigned to 24 hours of coverage. . . . . **Go to #30 on Page 7**
- ☐4 Other (please specify) __________________________________. . . . . **Go to #30 on Page 7**

29. What is the **duration** of the assigned daytime "**on-service**" period? *(Select one response only)*

- ☐1 5 day week (e.g., Mon-Fri)
- ☐2 7 day week (e.g., Mon-Sun; Sun-Sat)
- ☐3 1 month (e.g., 28 days, calendar month)
- ☐4 Other (please specify) ____

30. Is the surgeon assigned to **daytime** emergency general surgery coverage **freed of office or other clinical responsibilities** not related to emergency general surgery during the day?

- ☐1 Yes
- ☐2 No

31. How frequently would the surgeon assigned to **daytime** emergency general surgery coverage receive **clinical assistance** from the following? *(Select a response for each row)*

	Always	Often	Sometimes	Rarely	Never
a. Mid-level practitioner(s) (e.g., NP, PA)	☐1	☐2	☐3	☐4	☐5
b. Surgical resident(s)	☐1	☐2	☐3	☐4	☐5
c. Medical student(s)	☐1	☐2	☐3	☐4	☐5

32. How frequently would the surgeon assigned to **overnight** emergency general surgery coverage do the following? *(Select a response for each row)*

	Always	Often	Sometimes	Rarely	Never
a. Earn a stipend (beyond billing for services rendered) for covering emergency general surgery	☐1	☐2	☐3	☐4	☐5
b. Cover emergency general surgery at 2 or more hospitals	☐1	☐2	☐3	☐4	☐5
c. Provide in-house emergency general surgery coverage	☐1	☐2	☐3	☐4	☐5
d. Cover trauma at the same time as emergency general surgery	☐1	☐2	☐3	☐4	☐5
e. Cover one or more intensive care units at the same time as emergency general surgery	☐1	☐2	☐3	☐4	☐5
f. Be freed of patient care responsibilities the following day	☐1	☐2	☐3	☐4	☐5
g. Transfer care of a patient who does NOT undergo operation overnight to a sub-specialty surgeon (e.g., bleeding gastric tumor to surgical oncologist)	☐1	☐2	☐3	☐4	☐5
h. Transfer care of a patient who does NOT undergo operation overnight to a medical hospitalist	☐1	☐2	☐3	☐4	☐5

33. How frequently would the surgeon who performed an emergency general surgery **operation** while covering **overnight** do the following? *(Select a response for each row)*

	Always	Often	Sometimes	Rarely	Never
a. Transfer day to day management to a medical hospitalist or PCP	☐1	☐2	☐3	☐4	☐5
b. Round on the patient until discharge	☐1	☐2	☐3	☐4	☐5
c. See the patient in follow-up clinic	☐1	☐2	☐3	☐4	☐5
d. Admit the patient to him/herself for a post-discharge complication requiring admission	☐1	☐2	☐3	☐4	☐5
e. Transfer post-discharge care of the patient to a sub-specialty surgeon (e.g., Hartmann's procedure to colorectal surgeon)	☐1	☐2	☐3	☐4	☐5

34. How frequently would the surgeon assigned to **overnight** emergency general surgery coverage receive **clinical assistance** from the following? *(Select a response for each row)*

	Always	Often	Sometimes	Rarely	Never
a. Mid-level practitioner(s) (e.g., NP, PA)	☐1	☐2	☐3	☐4	☐5
b. Surgical resident(s)	☐1	☐2	☐3	☐4	☐5
c. Medical student(s)	☐1	☐2	☐3	☐4	☐5

Emergency General Surgery Infrastructure

The following questions pertain to your hospital's infrastructure for emergency general surgery patients.

35. Does your hospital designate **daytime operating room time** (block time) for previously unscheduled emergency general surgery cases (add-ons)?

☐1 Yes

☐2 No. . . . . **If *No*, go to #37**

36. *Approximately* how many days a week does your hospital have **designated block time** for emergency general surgery cases (add-on cases)? *(Select one response only)*

<1 day	1 day	2 days	3 days	4 days	5 days	>5 days
☐1	☐2	☐3	☐4	☐5	☐6	☐7

37. Does your hospital employ a **Program Manager** to oversee quality and delivery of care to emergency general surgery patients?

☐1 Yes

☐2 No. . . . . **If *No*, go to #39 on Page 9**

38. Is the Program Manager simultaneously responsible for any of the following? *(Select all that apply)*

- ☐1 Quality and delivery of care to trauma patients
- ☐2 Quality and delivery of care to elective general surgery patients
- ☐3 Other (please specify) ____

39. Which of the following *BEST* describes how the **clinical service** or **census** of emergency general surgery patients is organized? *(Select one response only)*

Emergency general surgery patients are cared for on…

- ☐1 Their own service/census
- ☐2 A combined service/census with elective general surgery patients
- ☐3 A combined service/census with trauma patients
- ☐4 A combined service/census with elective general surgery and trauma patients
- ☐5 Other (please specify) ____

40. Where do emergency general surgery patients who are **not critically ill** *typically* receive care? *(Select one response only)*

- ☐1 An assigned ward/floor
- ☐2 A ward/floor with other surgical patients
- ☐3 A ward/floor with medical patients
- ☐4 Other (please specify) ____

41. Where do **critically ill** emergency general surgery patients *typically* receive care? *(Select one response only)*

- ☐1 A Surgical ICU
- ☐2 A Trauma ICU
- ☐3 A combined Trauma/Surgical ICU
- ☐4 A combined Medical/Surgical ICU
- ☐5 A Medical ICU
- ☐6 Other (please specify) ____

42. Who manages critical care issues (e.g., ventilator management, glycemic control, septic shock) for emergency general surgery patients in this ICU? *(Select all that apply)*

- ☐1 The operating surgeon or a surgical colleague (i.e., "open" ICU)
- ☐2 A surgical critical care intensivist
- ☐3 An anesthesia critical care intensivist
- ☐4 A pulmonary critical care intensivist
- ☐5 Other (please specify) ____

43. Does your hospital have a **formal transfer agreement(s) with another hospital(s)** to send or receive emergency general surgery patients in need of higher level of care? *(Select Yes or No for send and receive)*

	Yes	No
a. Formal agreement(s) to SEND patients	☐ 1	☐ 2
b. Formal agreement(s) to RECEIVE patients	☐ 1	☐ 2

44. Regardless of formal transfer agreements, *approximately* what percentage of your hospital's total emergency general surgery patient volume is **transferred** to/from another hospital **each month**? (*Please provide your best estimate in the spaces below for both transferred IN and transferred OUT)*

a. Transferred IN _________%

b. Transferred OUT _________%

Emergency General Surgery Processes

The following questions pertain to processes of care for emergency general surgery patients at your hospital.

45. Does your hospital have **face-to-face hand-offs** for emergency general surgery patients in the morning and/or evening (e.g., signout rounds, cardflip rounds, am/pm report)? *(Select Yes or No for Morning and Evening)*

	Yes	No
a. Morning	☐ 1	☐ 2
b. Evening	☐ 1	☐ 2

If you answered NO for both Morning and Evening, go to #49 on Page 11

46. What **patients** are typically **discussed** at these hand-off meetings? *(Select all that apply in each row)*

	Discussed at Morning Signout	Discussed at Evening Signout	Not Discussed at Morning or Evening Signout
a. All patients on the emergency general surgery service/census	☐ 1	☐ 2	☐ 3
b. New emergency general surgery patients only	☐ 1	☐ 2	☐ 3
c. Emergency general surgery patients in the ICU	☐ 1	☐ 2	☐ 3
d. Emergency general surgery patients at risk for clinical deterioration	☐ 1	☐ 2	☐ 3
e. Other patients (e.g., trauma, elective general surgery)	☐ 1	☐ 2	☐ 3

47. Which **physicians** *typically* attend these hand-off meetings? *(Select all that apply in each row)*

	Attends Morning Signout	Attends Evening Signout	Does NOT Attend Morning or Evening Signout
a. Incoming surgeon who will be covering emergency general surgery	☐1	☐2	☐3
b. Outgoing surgeon who was covering emergency general surgery	☐1	☐2	☐3
c. Other surgeons not covering emergency general surgery that day	☐1	☐2	☐3
d. Other physician(s) (e.g., physiatry, geriatrics, psychiatry)	☐1	☐2	☐3
e. Incoming residents	☐1	☐2	☐3
f. Outgoing residents	☐1	☐2	☐3

48. Which **other staff** *typically* attend these hand-off meetings? *(Select all that apply in each row)*

	Attends Morning Signout	Attends Evening Signout	Does NOT Attend Morning or Evening Signout
a. Mid-level practitioners (e.g., NP, PA)	☐1	☐2	☐3
b. ICU nursing staff	☐1	☐2	☐3
c. Ward/floor nursing staff	☐1	☐2	☐3
d. Social services staff (e.g., social worker, case-manager)	☐1	☐2	☐3
e. Therapy staff (e.g., physical or occupational therapists)	☐1	☐2	☐3
f. Program Manager	☐1	☐2	☐3
g. Medical students	☐1	☐2	☐3
h. Other (please specify) ___________________________	☐1	☐2	☐3

49. In situations where your hospital does not conduct face-to-face handoffs, which of the following *BEST* describes how handoffs typically occur for emergency general surgery patients? *(Select one response only)*

Surgeons hand-off care of emergency general surgery patients by…

☐1 Telephoning the covering surgeon

☐2 Leaving a printed patient list for the covering surgeon

☐3 Sending an email to the covering surgeon

☐4 Not applicable (i.e., all handoffs occur face to face)

☐5 Other (please specify) ____

50. Does your hospital conduct a *dedicated* emergency general surgery **morbidity & mortality** review?

- ☐1 Yes. . . . . **If *Yes*, go to #52**
- ☐2 No

51. If your hospital does not conduct a dedicated emergency general surgery M&M, which of the following *BEST* describes where emergency general surgery patient morbidity and mortality is discussed? *(Select one response only)*

The morbidity and mortality of emergency general surgery patients is discussed…

- ☐1 At the departmental morbidity and mortality conference
- ☐2 At the hospital-wide morbidity and mortality conference
- ☐3 As needed when issues arise (e.g., sentinel event review, root cause analysis)
- ☐4 Other (please specify) ____

52. How frequently is this morbidity & mortality meeting conducted? *(Select one response only)*

- ☐1 Weekly
- ☐2 Monthly
- ☐3 Quarterly
- ☐4 Other (please specify) ____

53. Which **physicians** *typically* attend this morbidity & mortality meeting? *(Select all that apply)*

- ☐1 Surgeons who participate in emergency general surgery coverage at your hospital
- ☐2 Surgeon(s) from other subspecialties (e.g., colorectal, vascular, transplant)
- ☐3 Anesthesiologist(s) (who typically staff the OR)
- ☐4 Radiologist(s)
- ☐5 Intensivist(s) (from surgery, anesthesia, or pulmonary critical care)
- ☐6 Other physician(s) (e.g., physiatry, geriatrics, psychiatry)

54. Which **other staff** *typically* attend this morbidity & mortality meeting? *(Select all that apply)*

- ☐1 Mid-level practitioners
- ☐2 Program Manager
- ☐3 ICU nursing staff
- ☐4 Ward nursing staff
- ☐5 Social services staff (e.g., social worker, case-manager)
- ☐6 Therapy staff (e.g., physical or occupational therapists)
- ☐7 Other (please specify) ____

55. Has your hospital established the following emergency general surgery processes? *(Select a response for each row)*

	Yes	No
a. A prospective registry of emergency general surgery patients	☐ 1	☐ 2
b. An activation system (similar to trauma activations, e.g., Trauma STAT page; Level 1 Trauma) for unstable emergency general surgery patients who present to the ER	☐ 1	☐ 2
c. A protocol for identifying patients requiring ICU admission (e.g., due to hemodynamic lability or co-morbidities) after emergency general surgery operations	☐ 1	☐ 2
d. An outpatient follow-up clinic specifically for emergency general surgery patients	☐ 1	☐ 2

56. Does your hospital **monitor** the following measures? *(Select a response for each row)*

	Yes	No
a. Time to initial evaluation by the surgeon covering emergency general surgery after ER consultation	☐ 1	☐ 2
b. Time to source control after diagnosis of an intra-abdominal or soft-tissue infection (e.g., resection of organ causing peritoneal contamination; fascial debridement)	☐ 1	☐ 2
c. Time to start of operation after booking an emergent general surgery case	☐ 1	☐ 2

57. Does your hospital audit the following **unplanned events**? *(Select a response for each row)*

	Yes	No
a. Return to the operating room during the index hospitalization after initial emergency general surgery operation	☐ 1	☐ 2
b. Transfer of emergency general surgery patients back to an intensive care unit within 48 hours of discharge to the ward	☐ 1	☐ 2
c. Hospital re-admission within 30 days of discharge after an emergency general surgery operation	☐ 1	☐ 2
d. Return to the operating room within 30 days after an emergency general surgery operation (even if previously discharged)	☐ 1	☐ 2
e. Hospital re-admission within 30 days of discharge after a general surgery emergency that was managed non-operatively	☐ 1	☐ 2
f. Need for operation within 30 days of discharge after a general surgery emergency that was managed non-operatively	☐ 1	☐ 2

58. For each clinical scenario below, who would *typically* manage the patient at your hospital? Assume the patient initially presented to the emergency room. (*Select a response for each row)*

	**Surgeon covering emergency general surgery**	**Surgeon who operated on the patient**	**Sub-specialty surgeon** colorectal, gyn, ortho, urology, PRS thoracic, onc, bariatric, etc.	**Non-surgeon** hospitalist internist, GI, ID, etc.	**Patient *typically* transferred from ER to higher level of care *after* stabilization**
a. 45yo morbidly obese diabetic male with Fournier's gangrene	☐1	☐2	☐3	☐4	☐5
b. 60yo female 1 week s/p open abdominal hysterectomy with fascial dehiscence	☐1	☐2	☐3	☐4	☐5
c. 50yo male s/p screening colonoscopy with peritonitis	☐1	☐2	☐3	☐4	☐5
d. 32yo male 1 week s/p routine appendectomy with RLQ abscess	☐1	☐2	☐3	☐4	☐5
e. 37yo female 2 years s/p Roux-en-Y gastric bypass with internal hernia	☐1	☐2	☐3	☐4	☐5
f. 90yo female nursing home resident with sigmoid volvulus	☐1	☐2	☐3	☐4	☐5
g. 23yo female IV drug user with necrotizing soft tissue infection of her right forearm proximal to the wrist	☐1	☐2	☐3	☐4	☐5
h. 71yo male with esophageal perforation due to Boerhaave's syndrome	☐1	☐2	☐3	☐4	☐5
i. 40yo female 2 weeks s/p routine cholecystectomy with bile leak	☐1	☐2	☐3	☐4	☐5

59. Which statement *BEST* describes your hospital? *(Select one response only)*

My hospital's overall approach to emergency general surgery is…

☐1 A dedicated clinical team whose scope encompasses emergency general surgery (+/-trauma, +/- elective general surgery, +/- burns)

☐2 A traditional approach with an ad hoc "general surgeon on call" schedule. . . . . ***Go to* Page 16**

☐3 Other (please specify) ____. . . . . ***Go to* Page 16**

60. What do you call this dedicated emergency general surgery team (e.g., acute care surgery, emergency general surgery, surgical hospitalist)?

Name of team____

61. How is oversight of this dedicated clinical team structured? *(Select one response only)*

- ☐1 Within a division of our department of surgery
- ☐2 Within a section of our division of general surgery
- ☐3 Within a section of our division of trauma and critical care
- ☐4 Other (please specify) ____

62. In what year was this dedicated emergency general surgery team *fully* implemented?

Year_________ . . . . **If *2015*, Please fill in the month it was started here:_____________**

63. Does this team employ **mid-level practitioners** (e.g., NP, PA)?

- ☐1 Yes
- ☐2 No. . . . . **If *No*, go to #65**

64. What are the degree pathways of these mid-level practitioners? *(Select all that apply and indicate the number of practitioners on the team with each degree)*

- ☐1 Nurse Practitioner, NP/APRN/DNP (N = ________)
- ☐2 Physician Assistant, PA (N = ________)
- ☐3 Other ____ (N = ________)

65. Do **surgical trainees** *typically* rotate on this team?

- ☐1 Yes
- ☐2 No. . . . . **If *No*, go to Page 16**

66. What are the **post-graduate training levels** of these surgical trainees? *(Select all that apply and indicate the number of trainees on the team for each post-graduate level)*

- ☐1 PGY-1, intern (N = ________)
- ☐2 PGY-2, junior resident (N = ________)
- ☐3 PGY-3, mid-level resident (N = ________)
- ☐4 PGY-4, senior resident (N = ________)
- ☐5 PGY-5, chief resident (N = ________)
- ☐6 PGY-6 or higher, fellow (N = ________)

Thank you for completing the survey. Please return it in the postage-paid envelope.

We are grateful for your time and thoughtful responses.

67. Do you wish to receive a summary of the survey results?

☐1 Yes .... **If *Yes*, Please fill in your email here:**______________________________________

☐2 No

68. If you have any other comments regarding our survey content or about emergency surgery coverage in general, please use the space below to share them with us.

Contact us at egs-survey@umassmed.edu
