# Supplementary material for: Identifying the fundamental structures and processes of care contributing to emergency general surgery quality using a mixed-methods Donabedian approach
Source: BMC Med Res Methodol. 2020 Oct 2;20:247. doi: 10.1186/s12874-020-01096-7 (PMC7532630; doi:10.1186/s12874-020-01096-7)
Supplement: Supplementary file 5 — Additional file 5. Matrixed Table of Survey Question Alignment with Structure and Process Domains. [file 12874_2020_1096_MOESM5_ESM.docx]

| *Appendix 5. Survey Question Alignment with Structure and Process Domains* | | | | | | | | | | | | |
| --- | --- | --- | --- | --- | --- | --- | --- | --- | --- | --- | --- | --- |
|  | **Structure** | | | **Process** | | | | | | | **Combined** | |
|  | EGS Workforce | Hospital staff | Subspecialty services | Surgeon-patient contact | Communication | Continuity of care | EGS team implementation | Operating room access | Patient safety protocols | Performance improvement measures | Diagnostic radiology | Critical care resources |
| ***Hospital-Wide Structures & Processes*** |  | | |  | | | | | | |  | |
| **1: Round-the-clock (RTC) intensivist*** |  |  | X |  |  |  |  |  |  |  |  | X |
| **2. Intensivist delivery approach^** |  |  | X |  |  |  |  |  |  |  |  | X |
| In-house 24/7/365 |  |  | X |  |  |  |  |  |  |  |  | X |
| On-call when in-house not available |  |  | X |  |  |  |  |  |  |  |  | X |
| Tele-ICU when in house not available |  |  | X |  |  |  |  |  |  |  |  | X |
| Transfer agreements when in house not available |  |  | X |  |  |  |  |  | X |  |  | X |
| Other |  |  | X |  |  |  |  |  |  |  |  | X |
| **3. ERCP availability^** |  |  | X |  |  |  |  |  |  |  |  |  |
| Available/on call 24/7/365 |  |  | X |  |  |  |  |  |  |  |  |  |
| Weekdays only |  |  | X |  |  |  |  |  |  |  |  |  |
| None |  |  | X |  |  |  |  |  |  |  |  |  |
| Other |  |  | X |  |  |  |  |  |  |  |  |  |
| **4. Overnight OR availability*** |  |  |  |  |  |  |  | X |  |  |  |  |
| **5. Overnight OR staff availability – in house, on call, unavailable or unsure** |  |  |  |  |  |  |  | X |  |  |  |  |
| Scrub technicians |  | X |  |  |  |  |  | X |  |  |  |  |
| OR nursing staff |  | X |  |  |  |  |  | X |  |  |  |  |
| PACU/recovery room nursing staff |  | X |  |  |  |  |  | X |  |  |  |  |
| Anesthesiologists |  |  | X |  |  |  |  | X |  |  |  |  |
| CRNAs |  |  | X |  |  |  |  | X |  |  |  |  |
| Surgical pathologists for frozen section |  |  | X |  |  |  |  | X |  |  |  |  |
| **6. RTC ancillary staff*** |  | X |  |  |  |  |  |  |  |  |  |  |
| X-ray technician |  | X |  |  |  |  |  |  |  |  | X |  |
| Ultrasound technician |  | X |  |  |  |  |  |  |  |  | X |  |
| CT technician |  | X |  |  |  |  |  |  |  |  | X |  |
| Respiratory therapist |  | X |  |  |  |  |  |  |  |  |  | X |
| Clinical laboratory technician |  | X |  |  |  |  |  |  |  |  |  |  |
| Blood bank technician |  | X |  |  |  |  |  |  |  |  |  |  |
| **7. Frequency of radiographic studies ordered stat”** |  |  |  |  |  |  |  |  |  |  | X |  |
| CT Scan within 4 hours of order |  |  |  |  |  |  |  |  |  |  | X |  |
| Ultrasound within 4 hours of order |  |  |  |  |  |  |  |  |  |  | X |  |
| Radiology read within 2 hours of study completion |  |  |  |  |  |  |  |  |  |  | X |  |
| Study read overnight by tele-radiologist |  |  |  |  |  |  |  |  |  |  | X |  |
| Critical study findings communicated to physician |  |  |  |  | X |  |  |  | X |  | X |  |
| Interventional radiology available within 1 hour |  |  | X |  |  |  |  |  |  |  | X |  |
| **8. Managing clinical deterioration*** |  |  |  |  |  |  |  |  | X |  |  |  |
| Graded response strategy to identify at risk patients |  |  |  |  |  |  |  |  | X |  |  |  |
| Guidelines to escalate care for clinical deterioration |  |  |  |  |  |  |  |  | X |  |  |  |
| RTC availability of physicians to evaluate patient |  |  |  |  |  |  |  |  | X |  |  |  |
| **9. Clinical practice protocols*** |  |  |  |  |  |  |  |  |  |  |  |  |
| Blood products available urgently for transfusion |  |  |  |  |  |  |  |  | X |  |  | X |
| Code airway teams |  |  |  |  |  |  |  |  | X |  |  | X |
| *Risk factor based DVT/PE prevention* |  |  |  |  |  |  |  |  | X |  |  |  |
| **10. Urgent / emergent operations*** |  |  |  |  |  |  |  |  |  |  |  |  |
| Tiered system for booking urgent or emergent cases |  |  |  |  |  |  |  | X | X |  |  |  |
| Guidelines to defer elective operations |  |  |  |  |  |  |  | X | X |  |  |  |
| Protocol to rapidly reverse anticoagulant drugs |  |  |  |  |  |  |  |  | X |  |  |  |
| *Process ensuring oncologic f/u for new cancer dx* |  |  |  |  |  |  |  |  | X |  |  |  |
| Protocol ensuring adherence to surviving sepsis |  |  |  |  |  |  |  |  |  |  |  | X |
| ***11. Ever lack RTC EGS coverage**** |  |  |  |  |  |  |  |  |  |  |  |  |
| ***12. How often lack EGS coverage (%)*** |  |  |  |  |  |  |  |  |  |  |  |  |
| **13. Reasons for lacking EGS coverage^** |  |  |  |  |  |  |  |  |  |  |  |  |
| Lack of general surgery coverage |  |  |  |  |  |  |  | X |  |  |  |  |
| Lack of anesthesia coverage |  |  |  |  |  |  |  | X |  |  |  |  |
| Lack of OR staff |  |  |  |  |  |  |  | X |  |  |  |  |
| *Emergency room is on diversion* |  |  |  |  |  |  |  |  |  |  |  |  |
| *Other* |  |  |  |  |  |  |  |  |  |  |  |  |
|  | **Structure** | | | **Process** | | | | | | | **Combined** | |
|  | EGS Workforce | Hospital staff | Subspecialty services | Surgeon-patient contact | Communication | Continuity of care | EGS team implementation | Operating room access | Patient safety protocols | Performance improvement measures | Diagnostic radiology | Critical care resources |
| ***Emergency General Surgery Workforce*** |  | | |  | | | | | | |  | |
| **14. Surgeons providing EGS care^#^** | X |  |  |  |  |  |  |  |  |  |  |  |
| **15. Surgeon demographics^#^** | X |  |  |  |  |  |  |  |  |  |  |  |
| Female | X |  |  |  |  |  |  |  |  |  |  |  |
| >65 years old | X |  |  |  |  |  |  |  |  |  |  |  |
| Finished training within last 3 years | X |  |  |  |  |  |  |  |  |  |  |  |
| **16. Employment model^#^** | X |  |  |  |  |  |  |  |  |  |  |  |
| Hospital | X |  |  |  |  |  |  |  |  |  |  |  |
| Academic | X |  |  |  |  |  |  |  |  |  |  |  |
| Private | X |  |  |  |  |  |  |  |  |  |  |  |
| City/County/Federal | X |  |  |  |  |  |  |  |  |  |  |  |
| Locum tenens | X |  |  |  |  |  |  |  |  |  |  |  |
| Other | X |  |  |  |  |  |  |  |  |  |  |  |
| **17. Surgeons provide other types of surgical care*** | X |  |  |  |  |  |  |  |  |  |  |  |
| **18. What other types of surgical care^#^** | X |  |  |  |  |  |  |  |  |  |  |  |
| Trauma | X |  |  |  |  |  |  |  |  |  |  |  |
| Burns | X |  |  |  |  |  |  |  |  |  |  |  |
| Surgical Critical Care | X |  |  |  |  |  |  |  |  |  |  | X |
| Elective General Surgery | X |  |  |  |  |  |  |  |  |  |  |  |
| Other | X |  |  |  |  |  |  |  |  |  |  |  |
| **19. Surgeons have non-clinical roles*** | X |  |  |  |  |  |  |  |  |  |  |  |
| **20. What non-clinical roles^** | X |  |  |  |  |  |  |  |  |  |  |  |
| Surgical education | X |  |  |  |  |  |  |  |  |  |  |  |
| Research | X |  |  |  |  |  |  |  |  |  |  |  |
| Community outreach/public health | X |  |  |  |  |  |  |  |  |  |  |  |
| Administration | X |  |  |  |  |  |  |  |  |  |  |  |
| Other | X |  |  |  |  |  |  |  |  |  |  |  |
| ***21. Surgeons conduct EGS research**** | X |  |  |  |  |  |  |  |  |  |  |  |
| **22. Additional subspecialty training*** | X |  |  |  |  |  |  |  |  |  |  |  |
| **23. Which subspecialty training^#^** | X |  |  |  |  |  |  |  |  |  |  |  |
| Acute care surgery | X |  |  |  |  |  |  |  |  |  |  |  |
| Burn surgery | X |  |  |  |  |  |  |  |  |  |  |  |
| Surgical critical care | X |  |  |  |  |  |  |  |  |  |  | X |
| Trauma surgery | X |  |  |  |  |  |  |  |  |  |  |  |
| Breast surgery | X |  |  |  |  |  |  |  |  |  |  |  |
| Colorectal surgery | X |  |  |  |  |  |  |  |  |  |  |  |
| Endocrine surgery | X |  |  |  |  |  |  |  |  |  |  |  |
| Hepatobiliary surgery | X |  |  |  |  |  |  |  |  |  |  |  |
| Minimally invasive surgery | X |  |  |  |  |  |  |  |  |  |  |  |
| Thoracic surgery | X |  |  |  |  |  |  |  |  |  |  |  |
| Surgical oncology | X |  |  |  |  |  |  |  |  |  |  |  |
| Vascular surgery | X |  |  |  |  |  |  |  |  |  |  |  |
| Other | X |  |  |  |  |  |  |  |  |  |  |  |
| **24. Board certification status^#^** | X |  |  |  |  |  |  |  |  |  |  |  |
| American board of surgery in surgery | X |  |  |  |  |  |  |  |  |  |  |  |
| American osteopathic board of surgery in surgery | X |  |  |  |  |  |  |  |  |  |  |  |
| American board of surgery in critical care | X |  |  |  |  |  |  |  |  |  |  | X |
| Other | X |  |  |  |  |  |  |  |  |  |  |  |
| **25. Additional degrees*** | X |  |  |  |  |  |  |  |  |  |  |  |
| **26. Which additional degrees^#^** | X |  |  |  |  |  |  |  |  |  |  |  |
| Masters of public health | X |  |  |  |  |  |  |  |  |  |  |  |
| Masters of business administration | X |  |  |  |  |  |  |  |  |  |  |  |
| Masters of healthcare administration | X |  |  |  |  |  |  |  |  |  |  |  |
| Masters of education | X |  |  |  |  |  |  |  |  |  |  |  |
| Masters of science | X |  |  |  |  |  |  |  |  |  |  |  |
| Doctorate | X |  |  |  |  |  |  |  |  |  |  |  |
| Other | X |  |  |  |  |  |  |  |  |  |  |  |
|  | **Structure** | | | **Process** | | | | | | | **Combined** | |
|  | EGS Workforce | Hospital staff | Subspecialty services | Surgeon-patient contact | Communication | Continuity of care | EGS team implementation | Operating room access | Patient safety protocols | Performance improvement measures | Diagnostic radiology | Critical care resources |
| ***Emergency General Surgery Coverage*** |  | | |  | | | | | | |  | |
| **27. Surgeon compensation for uninsured patients*** | X |  |  | X |  |  |  |  |  |  |  |  |
| **28. Daytime surgeon coverage model^** |  |  |  | X |  |  | X | X |  |  |  |  |
| “On service” for 2 or more consecutive days |  |  |  | X |  |  | X | X |  |  |  |  |
| Assigned shift (8, 12, 14 hours long) |  |  |  | X |  |  | X | X |  |  |  |  |
| Assigned to 24 hours of coverage |  |  |  | X |  |  | X | X |  |  |  |  |
| Other |  |  |  | X |  |  | X | X |  |  |  |  |
| **29. Duration of “on-service”** |  |  |  | X |  |  | X | X |  |  |  |  |
| 5-day week |  |  |  | X |  |  | X | X |  |  |  |  |
| 7-day week |  |  |  | X |  |  | X | X |  |  |  |  |
| 1 month (28 days or calendar month) |  |  |  | X |  |  | X | X |  |  |  |  |
| Other |  |  |  | X |  |  | X | X |  |  |  |  |
| **30. Daytime surgeon free of office & other clinical responsibilities*** |  |  |  | X |  |  | X | X |  |  |  |  |
| **31. Frequency of daytime clinical assistance”** | X |  |  |  |  |  | X |  |  |  |  |  |
| Mid-levels | X |  |  |  |  |  | X |  |  |  |  |  |
| Surgical residents | X |  |  |  |  |  | X |  |  |  |  |  |
| Medical students | X |  |  |  |  |  | X |  |  |  |  |  |
| **32. Components of overnight EGS coverage”** |  |  |  |  |  |  |  |  |  |  |  |  |
| Stipend beyond billed services for covering |  |  |  | X |  |  |  |  |  |  |  |  |
| Covering at 2 or more hospitals |  |  |  |  |  |  | X | X |  |  |  |  |
| In-house |  |  |  | X |  |  | X | X |  |  |  |  |
| Cover trauma at the same time |  |  |  |  |  |  | X | X |  |  |  |  |
| Covering ICU at the same time |  |  |  |  |  |  | X | X |  |  |  |  |
| Freed from patient care following day |  |  |  | X |  |  |  | X |  |  |  |  |
| Transfer non-operative patient to subspecialist |  |  |  |  |  | X |  |  |  |  |  |  |
| Transfer non-operative patient to hospitalist |  |  |  |  |  | X |  |  |  |  |  |  |
| **33. Continuity following overnight operation”** |  |  |  |  |  | X |  |  |  |  |  |  |
| Transfer day to day management to hospitalist / PCP |  |  |  |  |  | X |  |  |  |  |  |  |
| Round on patient until discharge |  |  |  |  |  | X |  |  |  |  |  |  |
| See in f/u clinic |  |  |  |  |  | X |  |  |  |  |  |  |
| Post-discharge complication admitted to them |  |  |  |  |  | X |  |  |  |  |  |  |
| Transfer post-discharge care to subspecialist |  |  |  |  |  | X |  |  |  |  |  |  |
| **34. Frequency of overnight clinical assistance”** | X |  |  |  |  |  | X |  |  |  |  |  |
| Mid-levels | X |  |  |  |  |  | X |  |  |  |  |  |
| Surgical residents | X |  |  |  |  |  | X |  |  |  |  |  |
| Medical students | X |  |  |  |  |  | X |  |  |  |  |  |
| ***Emergency General Surgery Infrastructure*** |  | | |  | | | | | | |  | |
| **35. Designate OR time (block time) for add ons*** |  |  |  |  |  |  |  | X |  |  |  |  |
| **36. Days of designated block time^** |  |  |  |  |  |  |  | X |  |  |  |  |
| <1 day |  |  |  |  |  |  |  | X |  |  |  |  |
| 1 day |  |  |  |  |  |  |  | X |  |  |  |  |
| 2 days |  |  |  |  |  |  |  | X |  |  |  |  |
| 3 days |  |  |  |  |  |  |  | X |  |  |  |  |
| 4 days |  |  |  |  |  |  |  | X |  |  |  |  |
| 5 days |  |  |  |  |  |  |  | X |  |  |  |  |
| >5 days |  |  |  |  |  |  |  | X |  |  |  |  |
| **37. Program manager to oversee EGS patient care*** |  |  |  |  |  |  |  |  |  | X |  |  |
| **38. Program manager’s other patients^** |  |  |  |  |  |  |  |  |  | X |  |  |
| Trauma patients |  |  |  |  |  |  |  |  |  | X |  |  |
| Elective general surgery patients |  |  |  |  |  |  |  |  |  | X |  |  |
| Other |  |  |  |  |  |  |  |  |  | X |  |  |
| **39. Organization of EGS patient census/service^** |  |  |  | X |  |  |  |  |  |  |  |  |
| Dedicated census/service |  |  |  | X |  |  |  |  |  |  |  |  |
| Combined with elective general surgery |  |  |  | X |  |  |  |  |  |  |  |  |
| Combined with trauma |  |  |  | X |  |  |  |  |  |  |  |  |
| Combined with elective general surgery and trauma |  |  |  | X |  |  |  |  |  |  |  |  |
| Other |  |  |  | X |  |  |  |  |  |  |  |  |
| **40. Location of care for non-critically ill EGS patients^** |  |  |  | X |  |  |  |  |  |  |  |  |
| Assigned ward/floor |  |  |  | X |  |  |  |  |  |  |  |  |
| Ward/floor with other surgical patients |  |  |  | X |  |  |  |  |  |  |  |  |
| Ward/floor with medical patients |  |  |  | X |  |  |  |  |  |  |  |  |
| Other |  |  |  | X |  |  |  |  |  |  |  |  |
|  | **Structure** | | | **Process** | | | | | | | **Combined** | |
|  | EGS Workforce | Hospital staff | Subspecialty services | Surgeon-patient contact | Communication | Continuity of care | EGS team implementation | Operating room access | Patient safety protocols | Performance improvement measures | Diagnostic radiology | Critical care resources |
| **41. Location of care for critically ill EGS patients^** |  |  |  | X |  |  |  |  |  |  |  | X |
| Surgical ICU |  |  |  | X |  |  |  |  |  |  |  | X |
| Trauma ICU |  |  |  | X |  |  |  |  |  |  |  | X |
| Combined trauma/surgical ICU |  |  |  | X |  |  |  |  |  |  |  | X |
| Combined medical/surgical ICU |  |  |  | X |  |  |  |  |  |  |  | X |
| Medical ICU |  |  |  | X |  |  |  |  |  |  |  | X |
| Other |  |  |  | X |  |  |  |  |  |  |  | X |
| **42. Clinician managing issues for EGS patients in ICU^** |  |  |  |  |  |  |  |  |  |  |  | X |
| Operating surgeon or surgical colleague |  |  |  |  |  | X |  |  |  |  |  | X |
| Surgical critical care intensivist |  |  |  |  |  |  |  |  |  |  |  | X |
| Anesthesia critical care intensivist |  |  |  |  |  |  |  |  |  |  |  | X |
| Pulmonary critical care intensivist |  |  |  |  |  |  |  |  |  |  |  | X |
| Other |  |  |  |  |  |  |  |  |  |  |  | X |
| **43. Formal transfer agreement with another hospital*** |  |  |  | X |  |  |  |  | X |  |  |  |
| To send patients |  |  |  | X |  |  |  |  | X |  |  |  |
| To receive patients |  |  |  | X |  |  |  |  | X |  |  |  |
| **44. % of total EGS patient volume transferred (%)** |  |  |  | X |  |  |  |  | X |  |  |  |
| Transferred in |  |  |  | X |  |  |  |  | X |  |  |  |
| Transferred out |  |  |  | X |  |  |  |  | X |  |  |  |
| ***Emergency General Surgery Processes*** |  | | |  | | | | | | |  | |
| **45. Face-to-face handoffs*** |  |  |  |  | X |  |  |  |  |  |  |  |
| Morning |  |  |  |  | X |  |  |  |  |  |  |  |
| Evening |  |  |  |  | X |  |  |  |  |  |  |  |
| **46. Patients discussed at handoff – morning, evening, neither** |  |  |  |  | X |  |  |  |  |  |  |  |
| All patients on EGS census |  |  |  |  | X |  |  |  |  |  |  |  |
| New EGS patients only |  |  |  |  | X |  |  |  |  |  |  |  |
| EGS patients in ICU |  |  |  |  | X |  |  |  |  |  |  |  |
| EGS patients at risk for clinical deterioration |  |  |  |  | X |  |  |  | X |  |  |  |
| Other patients (e.g. trauma, elective general surgery) |  |  |  |  | X |  |  |  |  |  |  |  |
| **47. Physicians at handoff** **– morning, evening, neither** |  |  |  |  | X |  |  |  |  |  |  |  |
| Incoming surgeon covering EGS |  |  |  |  | X |  |  |  |  |  |  |  |
| Outgoing surgeon who was covering EGS |  |  |  |  | X |  |  |  |  |  |  |  |
| Other surgeons not covering EGS that day |  |  |  |  | X |  |  |  |  |  |  |  |
| Other physicians (eg physiatry, geriatrics) |  |  |  |  | X |  |  |  |  |  |  |  |
| Incoming residents |  |  |  |  | X |  |  |  |  |  |  |  |
| Outgoing residents |  |  |  |  | X |  |  |  |  |  |  |  |
| **48. Other staff at handoff – morning, evening, neither** |  |  |  |  | X |  |  |  |  |  |  |  |
| Mid-level practioners |  |  |  |  | X |  |  |  |  |  |  |  |
| ICU nursing staff |  |  |  |  | X |  |  |  |  |  |  |  |
| Social services staff (eg social worker, case-manager) |  |  |  |  | X |  |  |  |  |  |  |  |
| Therapy staff (eg physical or occupational therapist) |  |  |  |  | X |  |  |  |  |  |  |  |
| Program manager |  |  |  |  | X |  |  |  |  |  |  |  |
| Medical students |  |  |  |  | X |  |  |  |  |  |  |  |
| Other |  |  |  |  | X |  |  |  |  |  |  |  |
| **49. Alternatives to face-to-face handoffs^** |  |  |  |  | X |  |  |  |  |  |  |  |
| Telephone |  |  |  |  | X |  |  |  |  |  |  |  |
| Leaving printed patient list |  |  |  |  | X |  |  |  |  |  |  |  |
| Sending an email |  |  |  |  | X |  |  |  |  |  |  |  |
| N/A (ie all handoffs occur face to face) |  |  |  |  | X |  |  |  |  |  |  |  |
| Other |  |  |  |  | X |  |  |  |  |  |  |  |
| **50. Dedicated EGS M&M*** |  |  |  |  |  |  |  |  |  | X |  |  |
| **51. Frequency of EGS M&M^** |  |  |  |  |  |  |  |  |  | X |  |  |
| Weekly |  |  |  |  |  |  |  |  |  | X |  |  |
| Monthly |  |  |  |  |  |  |  |  |  | X |  |  |
| Quarterly |  |  |  |  |  |  |  |  |  | X |  |  |
| Other |  |  |  |  |  |  |  |  |  | X |  |  |
| **52. Physicians attending EGS M&M^** |  |  |  |  |  |  |  |  |  | X |  |  |
| Surgeons providing EGS coverage |  |  |  |  |  |  |  |  |  | X |  |  |
| Surgeons from other specialties |  |  |  |  |  |  |  |  |  | X |  |  |
| Anesthesiologists |  |  |  |  |  |  |  |  |  | X |  |  |
| Radiologists |  |  |  |  |  |  |  |  |  | X |  |  |
| Intensivists |  |  |  |  |  |  |  |  |  | X |  |  |
| Other physicians |  |  |  |  |  |  |  |  |  | X |  |  |
|  | **Structure** | | | **Process** | | | | | | | **Combined** | |
|  | EGS Workforce | Hospital staff | Subspecialty services | Surgeon-patient contact | Communication | Continuity of care | EGS team implementation | Operating room access | Patient safety protocols | Performance improvement measures | Diagnostic radiology | Critical care resources |
| **53. Other staff attending EGS M&M^** |  |  |  |  |  |  |  |  |  | X |  |  |
| Mid-level practioners |  |  |  |  |  |  |  |  |  | X |  |  |
| Program manager |  |  |  |  |  |  |  |  |  | X |  |  |
| ICU nursing staff |  |  |  |  |  |  |  |  |  | X |  |  |
| Ward nursing staff |  |  |  |  |  |  |  |  |  | X |  |  |
| Social services staff (eg social worker, case-manager) |  |  |  |  |  |  |  |  |  | X |  |  |
| Therapy staff (eg physical or occupational therapist) |  |  |  |  |  |  |  |  |  | X |  |  |
| Other |  |  |  |  |  |  |  |  |  | X |  |  |
| **54. EGS patient discussed if not dedicated M&M^** |  |  |  |  |  |  |  |  |  | X |  |  |
| Department M&M |  |  |  |  |  |  |  |  |  | X |  |  |
| Hospital-wide M&M |  |  |  |  |  |  |  |  |  | X |  |  |
| As needed when issues arise |  |  |  |  |  |  |  |  |  | X |  |  |
| Other |  |  |  |  |  |  |  |  |  | X |  |  |
| **55. EGS specific processes** |  |  |  |  |  |  |  |  |  |  |  |  |
| Prospective registry EGS patients |  |  |  |  |  |  |  |  |  | X |  |  |
| Activation system for unstable EGS patients in ER |  |  |  | X |  |  |  |  | X |  |  |  |
| Protocol to identify post-op EGS patients needing ICU care |  |  |  | X |  |  |  |  |  |  |  | X |
| Dedicated EGS outpatient f/u clinic |  |  |  | X |  | X |  |  |  |  |  |  |
| **56. Monitor care promptness metrics*** |  |  |  |  |  |  |  |  |  | X |  |  |
| Time to initial evaluation after ER consultation |  |  |  |  |  |  |  |  |  | X |  |  |
| Time to source control after dx intra-abdominal or soft-tissue infection |  |  |  |  |  |  |  |  |  | X |  |  |
| Time to start of operation after booking EGS case |  |  |  |  |  |  |  |  |  | X |  |  |
| **57. Audit in place for unplanned events*** |  |  |  |  |  |  |  |  |  | X |  |  |
| Return to OR during index hospitalization |  |  |  |  |  |  |  |  |  | X |  |  |
| Return to ICU within 48hrs of discharge to ward |  |  |  |  |  |  |  |  |  | X |  |  |
| Return to OR within 30 days of discharge |  |  |  |  |  |  |  |  |  | X |  |  |
| Re-admission within 30 days |  |  |  |  |  |  |  |  |  | X |  |  |
| Need operation within 30 days of discharge following non-operative management of EGS dx |  |  |  |  |  |  |  |  |  | X |  |  |
| ***58. Clinician managing clinical scenario – EGS, surgeon who operated on patient, subspecialist, non-surgeon, transferred out after stabilization*** |  |  |  |  |  |  |  |  |  |  |  |  |
| *Morbidly obese diabetic with Fournier’s gangrene* |  |  |  |  |  |  |  |  |  |  |  |  |
| *60yo 1 week s/p open hysterectomy with fascial dehiscence* |  |  |  |  |  |  |  |  |  |  |  |  |
| *50yo male s/p screening colonoscopy with peritonitis* |  |  |  |  |  |  |  |  |  |  |  |  |
| *1 week s/p routine appendectomy with RLQ abscess* |  |  |  |  |  |  |  |  |  |  |  |  |
| *37yo female 2 years s/p Roux-en-Y gastric bypass with internal hernia* |  |  |  |  |  |  |  |  |  |  |  |  |
| *90yo nursing home resident with sigmoid volvulus* |  |  |  |  |  |  |  |  |  |  |  |  |
| *IVDU with necrotizing soft tissue infection of forearm proximal to wrist* |  |  |  |  |  |  |  |  |  |  |  |  |
| *71yo with esophageal perforation due to Boerhaave’s syndrome* |  |  |  |  |  |  |  |  |  |  |  |  |
| *2 weeks s/p routine cholecystectomy with bile leak* |  |  |  |  |  |  |  |  |  |  |  |  |
| **59. Hospital’s approach to EGS** |  |  |  |  |  |  | X |  |  |  |  |  |
| Dedicated clinical team encompassing EGS +/- burns +/-trauma +/- elective general surgery |  |  |  |  |  |  | X |  |  |  |  |  |
| Traditional general surgeon on call approach |  |  |  |  |  |  | X |  |  |  |  |  |
| Other |  |  |  |  |  |  | X |  |  |  |  |  |
| **60. Name of dedicated EGS team (short answer)** |  |  |  |  |  |  | X |  |  |  |  |  |
| **61. Oversight for dedicated team** |  |  |  |  |  |  | X |  |  |  |  |  |
| A division within the department of surgery |  |  |  |  |  |  | X |  |  |  |  |  |
| Within section of general surgery division |  |  |  |  |  |  | X |  |  |  |  |  |
| Within section of trauma and critical care division |  |  |  |  |  |  | X |  |  |  |  |  |
| Other |  |  |  |  |  |  | X |  |  |  |  |  |
| **62. Year dedicated EGS team implemented** |  |  |  |  |  |  | X |  |  |  |  |  |
| **63. Mid-level practioners on team*** | X |  |  |  |  |  | X |  |  |  |  |  |
| **64. Degree pathways of mid-level practioners^#^** | X |  |  |  |  |  | X |  |  |  |  |  |
| Nurse practioner | X |  |  |  |  |  | X |  |  |  |  |  |
| Physician assistant | X |  |  |  |  |  | X |  |  |  |  |  |
| Other | X |  |  |  |  |  | X |  |  |  |  |  |
|  | **Structure** | | | **Process** | | | | | | | **Combined** | |
|  | EGS Workforce | Hospital staff | Subspecialty services | Surgeon-patient contact | Communication | Continuity of care | EGS team implementation | Operating room access | Patient safety protocols | Performance improvement measures | Diagnostic radiology | Critical care resources |
| **65. Surgical trainees typically rotate*** | X |  |  |  |  |  | X |  |  |  |  |  |
| **66. Post-graduate training levels^#^** | X |  |  |  |  |  | X |  |  |  |  |  |
| PGY-1 | X |  |  |  |  |  | X |  |  |  |  |  |
| PGY-2 | X |  |  |  |  |  | X |  |  |  |  |  |
| PGY-3 | X |  |  |  |  |  | X |  |  |  |  |  |
| PGY-4 | X |  |  |  |  |  | X |  |  |  |  |  |
| PGY-5 | X |  |  |  |  |  | X |  |  |  |  |  |
| PGY-6 | X |  |  |  |  |  | X |  |  |  |  |  |
| Italicized queries were general survey questions that did not fall into the specific domains for this study.  *Yes/No  ^Multiple Choice  ^#^ Number of category (eg 4 surgeons provide EGS care)  “Frequency of item - always, often, sometimes, rarely or never | | | | | | | | | | | | |
