# Supplementary material for: Identifying the fundamental structures and processes of care contributing to emergency general surgery quality using a mixed-methods Donabedian approach
Source: BMC Med Res Methodol. 2020 Oct 2;20:247. doi: 10.1186/s12874-020-01096-7 (PMC7532630; doi:10.1186/s12874-020-01096-7)
Supplement: Supplementary file 6 — Additional file 6. ICD-9 and ICD-10 Diagnosis Codes Used to Identify Emergency General Surgery Cases. [file 12874_2020_1096_MOESM6_ESM.docx]

| **Appendix 6. ICD-9 and ICD-10 Diagnosis Codes Used to Identify Emergency General Surgery Cases** | | | | |
| --- | --- | --- | --- | --- |
| **Diagnosis** | **ICD-9 Code** | | **ICD-10 Code** | |
| **Necrotizing Soft Tissue Infections (NSTI)** | | | | |
| **NSTI** | 040.0 | Gas gangrene | A48.0 | Gas gangrene |
|  | 728.86 | Necrotizing fasciitis | M72.6 | Necrotizing fasciitis |
|  | 785.4 | Cutaneous gangrene | I96. | Gangrene, not elsewhere classified |
|  | 608.83 | Fournier’s gangrene | N49.3 | Fournier gangrene |
| **Small Bowel Obstruction** | | | | |
| **Internal Blockage** | 560.30 | Impaction of intestine, unspecified | K56.49 | Other impaction of intestine |
|  | 560.39 | Other impaction of intestine |  |  |
|  | 560.31 | Gallstone ileus | K56.3 | Gallstone ileus |
|  | 560.89 | Other specified intestinal obstruction | K56.69 | Other intestinal obstruction |
| **Blockage Due to Twists or Scarring** | 560.2 | Volvulus | K56.2 | Volvulus |
|  | 560.81 | Intestinal or peritoneal adhesions with obstruction | K56.5 | Intestinal adhesions [bands] with obstruction (postprocedural) (postinfection) |
|  | 552.00 | Unilateral or unspecified hernia with obstruction | K41.30 | Unilateral femoral hernia, with obstruction, without gangrene, not specified as recurrent |
|  | 552.01 | Unilateral or unspecified recurrent hernia with obstruction | K41.31 | Unilateral femoral hernia, with obstruction, without gangrene, recurrent |
|  | 552.02 | Bilateral hernia with obstruction | K41.00 | Bilateral femoral hernia, with obstruction, without gangrene, not specified as recurrent |
|  | 552.03 | Bilateral recurrent hernia with obstruction | K41.01 | Bilateral femoral hernia, with obstruction, without gangrene, recurrent |
|  | 552.20 | Ventral hernia, unspecified with obstruction | K43.6 | Other and unspecified ventral hernia with obstruction, without gangrene |
|  | 552.29 | Other ventral hernia with obstruction |  |  |
|  | 552.21 | Incisional hernia, with obstruction | K43.0 | Incisional hernia with obstruction, without gangrene |
|  | 552.29 | Other ventral hernia with obstruction | K43.3 | Parastomal hernia with obstruction, without gangrene |
| **Unspecified Cause of Blockage** | 560.9 | Unspecified intestinal obstruction | K56.60 | Unspecified intestinal obstruction |
| **Appendicitis** | | | | |
| **Appendicitis** | 540.0 | Appendicitis with generalized peritonitis | K35.2 | Acute appendicitis with generalized peritonitis |
|  | 540.1 | Appendicitis with peritoneal abscess | K35.3 | Acute appendicitis with localized peritonitis |
|  | 540.9 | Appendicitis without mention of peritonitis | K35.80 | Unspecified acute appendicitis |
|  |  |  | K35.89 | Other acute appendicitis |
|  | 541 | Appendicitis, unqualified | K37 | Unspecified appendicitis |
|  | 542 | Other appendicitis | K36 | Other appendicitis |
| **Biliary Disease** | | | | |
| **Simple or Isolated Cholecystitis** | 574.00 | Calculus of gallbladder with acute cholecystitis without obstruction | K80.00 | Calculus of gallbladder with acute cholecystitis without obstruction |
|  |  |  | K80.12 | Calculus of gallbladder with acute and chronic cholecystitis without obstruction |
|  | 574.01 | Calculus of gallbladder with acute cholecystitis with obstruction | K80.01 | Calculus of gallbladder with acute cholecystitis with obstruction |
|  |  |  | K80.13 | Calculus of gallbladder with acute and chronic cholecystitis with obstruction |
|  | 574.10 | Calculus of gallbladder with other cholecystitis without obstruction | K80.18 | Calculus of gallbladder with other cholecystitis without obstruction |
|  |  |  | K80.10 | Calculus of gallbladder with chronic cholecystitis without obstruction |
|  |  |  | K80.12 | Calculus of gallbladder with acute and chronic cholecystitis without obstruction |
|  | 574.11 | Calculus of gallbladder with other cholecystitis with obstruction | K80.11 | Calculus of gallbladder with chronic cholecystitis with obstruction |
|  |  |  | K80.13 | Calculus of gallbladder with acute and chronic cholecystitis with obstruction |
|  |  |  | K80.19 | Calculus of gallbladder with other cholecystitis with obstruction |
|  | 575.0 | Acute cholecystitis | K81.0 | Acute cholecystitis |
|  | 575.10 | Cholecystitis, unspecified | K81.9 | Cholecystitis, unspecified |
|  | 575.11 | Chronic cholecystitis | K81.1 | Chronic cholecystitis |
|  | 575.12 | Acute and chronic cholecystitis | K81.2 | Acute cholecystitis with chronic cholecystitis |
|  | 575.2 | Obstruction of gallbladder | K82.0 | Obstruction of gallbladder |
|  | 575.3 | Hydrops of gallbladder | K82.1 | Hydrops of gallbladder |
|  | 575.4 | Perforation of gallbladder | K82.2 | Perforation of gallbladder |
| **Cholecystitis and Choledocho-lithiasis** | 574.30 | Calculus of bile duct with acute cholecystitis without obstruction | K80.42 | Calculus of bile duct with acute cholecystitis without obstruction |
|  |  |  | K80.46 | Calculus of bile duct with acute and chronic cholecystitis without obstruction |
|  | 574.31 | Calculus of bile duct with acute cholecystitis with obstruction | K80.43 | Calculus of bile duct with acute cholecystitis with obstruction |
|  |  |  | K80.47 | Calculus of bile duct with acute and chronic cholecystitis with obstruction |
|  | 574.40 | Calculus of bile duct with other cholecystitis without obstruction | K80.44 | Calculus of bile duct with chronic cholecystitis without obstruction |
|  |  |  | K80.46 | Calculus of bile duct with acute and chronic cholecystitis without obstruction |
|  |  |  | K80.40 | Calculus of bile duct with cholecystitis, unspecified, without obstruction |
|  | 574.60 | Calculus of gallbladder and bile duct with acute cholecystitis without obstruction | K80.62 | Calculus of gallbladder and bile duct with acute cholecystitis without obstruction |
|  | 574.61 | Calculus of gallbladder and bile duct with acute cholecystitis with obstruction | K80.63 | Calculus of gallbladder and bile duct with acute cholecystitis with obstruction |
|  | 574.70 | Calculus of gallbladder and bile duct with other cholecystitis without obstruction | K80.64 | Calculus of gallbladder and bile duct with chronic cholecystitis without obstruction |
|  |  |  | K80.60 | Calculus of gallbladder and bile duct with cholecystitis, unspecified, without obstruction |
|  | 574.71 | Calculus of gallbladder and bile duct with other cholecystitis with obstruction | K80.61 | Calculus of gallbladder and bile duct with cholecystitis, unspecified, with obstruction |
|  |  |  | K80.65 | Calculus of gallbladder and bile duct with chronic cholecystitis with obstruction |
|  | 574.80 | Calculus of gallbladder and bile duct with acute and chronic cholecystitis without obstruction | K80.66 | Calculus of gallbladder and bile duct with acute and chronic cholecystitis without obstruction |
|  | 574.81 | Calculus of gallbladder and bile duct with acute and chronic cholecystitis with obstruction | K80.67 | Calculus of gallbladder and bile duct with acute and chronic cholecystitis with obstruction |
|  | 575.41 | Calculus of bile duct with other cholecystitis with obstruction | K80.41 | Calculus of bile duct with cholecystitis, unspecified, with obstruction |
|  |  |  | K80.45 | Calculus of bile duct with chronic cholecystitis with obstruction |
| **Choledocho-lithiasis** | 574.51 | Calculus of bile duct without mention of cholecystitis, with obstruction | K80.51 | Calculus of bile duct without cholangitis or cholecystitis with obstruction |
|  | 574.91 | Calculus of gallbladder and bile duct without cholecystitis, with obstruction | K80.71 | Calculus of gallbladder and bile duct without cholecystitis with obstruction |
| **Cholangitis** | 576.1 | Cholangitis | K83.0 | Cholangitis |
|  |  |  | K80.30 | Calculus of bile duct with cholangitis, unspecified, without obstruction |
|  |  |  | K80.31 | Calculus of bile duct with cholangitis, unspecified, with obstruction |
|  |  |  | K80.32 | Calculus of bile duct with acute cholangitis without obstruction |
|  |  |  | K80.33 | Calculus of bile duct with acute cholangitis with obstruction |
|  |  |  | K80.34 | Calculus of bile duct with chronic cholangitis without obstruction |
|  |  |  | K80.35 | Calculus of bile duct with chronic cholangitis with obstruction |
|  |  |  | K80.36 | Calculus of bile duct with acute and chronic cholangitis without obstruction |
|  |  |  | K80.37 | Calculus of bile duct with acute and chronic cholangitis with obstruction |
| **Pancreatitis** | | | | |
| **Acute Pancreatitis** | 577.0 | Acute pancreatitis – includes abscess and necrosis of pancreas, infected pancreas, as well as the following subcategories of pancreatitis: acute, edematous, hemorrhagic, recurrent, annular, apoplectic, calcareous, gangrenous, hemorrhagic, acute interstitial, malignant, subacute, and suppurative. | K85.9 | Acute pancreatitis, unspecified |
| **Gallstone Pancreatitis** | 577.0 with 575.0 | Acute pancreatitis and acute cholecystitis | K85.1 | Biliary acute pancreatitis |
| **Upper Gastrointestinal Emergencies** | | | | |
| **Peptic Ulcer Disease with Bleeding** | 531.0x | Acute gastric ulcer w/ hemorrhage | K25.0 | Acute gastric ulcer with hemorrhage |
|  | 531.01 | Acute gastric ulcer with hemorrhage, with obstruction |  |  |
|  | 531.2x | Acute gastric ulcer w/ hemorrhage & perforation | K25.2 | Acute gastric ulcer with both hemorrhage and perforation |
|  | 531.4x | Chronic gastric ulcer w/ hemorrhage | K25.4 | Chronic or unspecified gastric ulcer with hemorrhage |
|  | 531.6x | Chronic gastric ulcer w/ hemorrhage & perforation | K25.6 | Chronic or unspecified gastric ulcer with both hemorrhage and perforation |
|  | 532.0x | Acute duodenal ulcer w/ hemorrhage | K26.0 | Acute duodenal ulcer with hemorrhage |
|  | 532.2x | Acute duodenal ulcer w/ hemorrhage & perforation | K26.2 | Acute duodenal ulcer with both hemorrhage and perforation |
|  | 532.4x | Chronic duodenal ulcer w/ hemorrhage | K26.4 | Chronic or unspecified duodenal ulcer with hemorrhage |
|  | 532.6x | Chronic duodenal ulcer w/ hemorrhage & perforation | K26.6 | Chronic or unspecified duodenal ulcer with both hemorrhage and perforation |
|  | 533.0x | Acute peptic ulcer site unclassified w/ hemorrhage | K27.0 | Acute peptic ulcer, site unspecified, with hemorrhage |
|  | 533.2x | Acute peptic ulcer site w/ hemorrhage & perforation | K27.2 | Acute peptic ulcer, site unspecified, with both hemorrhage and perforation |
|  | 533.4x | Chronic peptic ulcer site w/ hemorrhage | K27.4 | Chronic or unspecified peptic ulcer, site unspecified, with hemorrhage |
|  | 533.6x | Chronic peptic ulcer site w/ hemorrhage & perforation | K27.6 | Chronic or unspecified peptic ulcer, site unspecified, with both hemorrhage and perforation |
|  | 534.0x | Acute gastrojejunal ulcer unclassified w/ hemorrhage | K28.0 | Acute gastrojejunal ulcer with hemorrhage |
|  | 534.2x | Acute gastrojejunal ulcer w/ hemorrhage & perforation | K28.2 | Acute gastrojejunal ulcer with both hemorrhage and perforation |
|  | 534.4x | Chronic gastrojejunal ulcer w/ hemorrhage | K28.4 | Chronic or unspecified gastrojejunal ulcer with hemorrhage |
|  | 534.6x | Chronic gastrojejunal ulcer w/ hemorrhage & perforation | K28.6 | Chronic or unspecified gastrojejunal ulcer with both hemorrhage and perforation |
|  | 535.01 | Acute gastritis with hemorrhage | K29.01 | Acute gastritis with bleeding |
|  | 535.11 | Atrophic gastritis with hemorrhage | K29.41 | Chronic atrophic gastritis with bleeding |
|  |  |  | K29.51 | Unspecified chronic gastritis with bleeding |
|  |  |  | K29.31 | Chronic superficial gastritis with bleeding |
|  | 535.21 | Gastric mucosal hypertrophy with hemorrhage | K29.61 | Other gastritis with bleeding |
|  | 535.41 | Other specified gastritis with hemorrhage |  |  |
|  | 535.31 | Alcoholic gastritis with hemorrhage | K29.21 | Alcoholic gastritis with bleeding |
|  | 535.51 | Unspecified gastritis and gastroduodenitis with hemorrhage | K29.71 | Gastritis, unspecified, with bleeding |
|  |  |  | K29.91 | Gastroduodenitis, unspecified, with bleeding |
|  | 535.61 | Duodenitis with hemorrhage | K29.81 | Duodenitis with bleeding |
|  | 535.71 | Eosinophilic gastritis with hemorrhage | K52.81 | Eosinophilic gastritis or gastroenteritis |
| **Gastric Bleeding** | 537.83 | Angiodysplasia of stomach and duodenum with hemorrhage | K31.811 | Angiodysplasia of stomach and duodenum with bleeding |
|  | 537.84 | Dieulafoy lesion (hemorrhagic) of stomach and duodenum | K31.82 | Dieulafoy lesion (hemorrhagic) of stomach and duodenum |
|  | 151.0 with^ | Malignant neoplasm of cardia with GI hemorrhage | C16.0 with^^ | Malignant neoplasm of cardia |
|  | 151.1 with^ | Malignant neoplasm of pylorus with GI hemorrhage | C16.4 with^^ | Malignant neoplasm of pylorus |
|  | 151.2 with^ | Malignant neoplasm of pyloric antrum with GI hemorrhage | C16.3 with^^ | Malignant neoplasm of pyloric antrum |
|  | 151.3 with^ | Malignant neoplasm of fundus of stomach with GI hemorrhage | C16.1 with^^ | Malignant neoplasm of fundus of stomach |
|  | 151.4 with^ | Malignant neoplasm of body of stomach with GI hemorrhage | C16.2 with^^ | Malignant neoplasm of body of stomach |
|  | 151.5 with^ | Malignant neoplasm of lesser curvature, unspecified with GI hemorrhage | C16.5 with^^ | Malignant neoplasm of lesser curvature of stomach, unspecified |
|  | 151.6 with^ | Malignant neoplasm of greater curvature, unspecified with GI hemorrhage | C16.6 with^^ | Malignant neoplasm of greater curvature of stomach, unspecified |
|  | 151.8 with^ | Malignant neoplasm of other specified sites of stomach with GI hemorrhage | C16.8 with^^ | Malignant neoplasm of overlapping sites of stomach |
|  | 151.9 with^ | Malignant neoplasm of stomach, unspecified with GI hemorrhage | C16.9 with^^ | Malignant neoplasm of stomach, unspecified |
|  | 211.1 with^ | Benign neoplasm of stomach with GI hemorrhage | D13.1 with^^ | Benign neoplasm of stomach |
|  | 211.1 with^ | Benign neoplasm of stomach with GI hemorrhage | K31.7 with^^ | Polyp of stomach and duodenum |
|  | 456.8 with^ | Varices of other site with GI hemorrhage | I86.4 with^^ | Gastric varices |
| **Small Bowel Bleeding** | 562.02 | Diverticulosis of small intestine with hemorrhage | K57.11 | Diverticulosis of small intestine without perforation or abscess with bleeding |
|  | 562.02 | Diverticulosis of small intestine with hemorrhage | K57.51 | Diverticulosis of both small and large intestine without perforation or abscess with bleeding |
|  | 562.03 | Diverticulitis of small intestine with hemorrhage | K57.01 | Diverticulitis of small intestine with perforation and abscess with bleeding |
|  | 562.03 | Diverticulitis of small intestine with hemorrhage | K57.13 | Diverticulitis of small intestine without perforation or abscess with bleeding |
|  | 562.03 | Diverticulitis of small intestine with hemorrhage | K57.41 | Diverticulitis of both small and large intestine with perforation and abscess with bleeding |
|  | 562.03 | Diverticulitis of small intestine with hemorrhage | K57.53 | Diverticulitis of both small and large intestine without perforation or abscess with bleeding |
|  | 152.0 with^ | Malignant neoplasm of duodenum with GI hemorrhage | C17.0 with^^ | Malignant neoplasm of duodenum |
|  | 152.1 with^ | Malignant neoplasm of jejunum with GI hemorrhage | C17.1 with^^ | Malignant neoplasm of jejunum |
|  | 152.2 with^ | Malignant neoplasm of ileum with GI hemorrhage | C17.2 with^^ | Malignant neoplasm of ileum |
|  | 152.3 with^ | Malignant neoplasm of Meckel's diverticulum with GI hemorrhage | C17.3 with^^ | Meckel's diverticulum, malignant |
|  | 152.8 with^ | Malignant neoplasm of other specified site of small intestine with GI hemorrhage | C17.8 with^^ | Malignant neoplasm of overlapping sites of small intestine |
|  | 152.9 with^ | Malignant neoplasm of small intestine, unspecified with GI hemorrhage | C17.9 with^^ | Malignant neoplasm of small intestine, unspecified |
|  | 211.2 with^ | Benign neoplasm of small intestine with GI hemorrhage | D13.2 with^^ | Benign neoplasm of duodenum |
|  | 211.2 with^ | Benign neoplasm of small intestine with GI hemorrhage | D13.30 with^^ | Benign neoplasm of unspecified part of small intestine |
|  | 211.2 with^ | Benign neoplasm of small intestine with GI hemorrhage | D13.39 with^^ | Benign neoplasm of other parts of small intestine |
| **Other Upper GI Bleeding** | 751.0 with^ | Meckel's diverticulum with GI hemorrhage | Q43.0 | Meckel's diverticulum (displaced) (hypertrophic) |
|  | 003.0 | Salmonella gastroenteritis | A02.0 | Salmonella enteritis |
|  | 558.1 with^ | Radiation enteritis with GI hemorrhage | K52.0 with^^ | Gastroenteritis and colitis due to radiation |
| **Upper GI Perforation or Strangulation** | | | | |
| **Gastric Ulcer Perforation** | 531.11 | Gastric ulcer, acute, with perforation, with obstruction | K25.1 | Acute gastric ulcer with perforation |
|  | 531.20 | Gastric ulcer, acute, with hemorrhage and perforation, without obstruction | K25.2 | Acute gastric ulcer with both hemorrhage and perforation |
|  | 531.21 | Gastric ulcer, acute, with hemorrhage and perforation, with obstruction |  |  |
|  | 531.50 | Gastric ulcer, chronic or unspecified, with perforation, without obstruction | K25.5 | Chronic or unspecified gastric ulcer with perforation |
|  | 531.51 | Gastric ulcer, chronic or unspecified, with perforation, with obstruction |  |  |
|  | 531.60 | Gastric ulcer, chronic or unspecified, with hemorrhage and perforation, without obstruction | K25.6 | Chronic or unspecified gastric ulcer with both hemorrhage and perforation |
|  | 531.61 | Gastric ulcer, chronic or unspecified, with hemorrhage and perforation, with obstruction |  |  |
| **Duodenal Ulcer Perforation** | 532.10 | Duodenal ulcer, acute, with perforation, without obstruction | K26.1 | Acute duodenal ulcer with perforation |
|  | 532.11 | Duodenal ulcer, acute, with perforation, with obstruction |  |  |
|  | 532.20 | Duodenal ulcer, acute, with hemorrhage and perforation, without obstruction | K26.2 | Acute duodenal ulcer with both hemorrhage and perforation |
|  | 532.21 | Duodenal ulcer, acute, with hemorrhage and perforation, with obstruction |  |  |
|  | 532.50 | Duodenal ulcer, chronic or unspecified, with perforation, without obstruction | K26.5 | Chronic or unspecified duodenal ulcer with perforation |
|  | 532.51 | Duodenal ulcer, chronic or unspecified, with perforation, with obstruction |  |  |
|  | 532.60 | Duodenal ulcer, chronic or unspecified, with hemorrhage and perforation, without obstruction | K26.6 | Chronic or unspecified duodenal ulcer with both hemorrhage and perforation |
|  | 532.61 | Duodenal ulcer, chronic or unspecified, with hemorrhage and perforation, with obstruction |  |  |
| **Peptic Ulcer Perforation** | 533.10 | Peptic ulcer, acute, with perforation, without obstruction | K27.1 | Acute peptic ulcer, site unspecified, with perforation |
|  | 533.11 | Peptic ulcer, acute, with perforation, with obstruction |  |  |
|  | 533.20 | Peptic ulcer, acute, with hemorrhage and perforation, without obstruction | K27.2 | Acute peptic ulcer, site unspecified, with both hemorrhage and perforation |
|  | 533.21 | Peptic ulcer, acute, with hemorrhage and perforation, with obstruction |  |  |
|  | 533.50 | Peptic ulcer, chronic or unspecified, with perforation, without obstruction | K27.5 | Chronic or unspecified peptic ulcer, site unspecified, with perforation |
|  | 533.51 | Peptic ulcer, chronic or unspecified, with perforation, with obstruction |  |  |
|  | 533.60 | Peptic ulcer, chronic or unspecified, with hemorrhage and perforation, without obstruction | K27.6 | Chronic or unspecified peptic ulcer, site unspecified, with both hemorrhage and perforation |
|  | 533.61 | Peptic ulcer, chronic or unspecified, with hemorrhage and perforation, with obstruction |  |  |
| **Gastrojejunal Ulcer Perforation** | 534.10 | Gastrojejunal ulcer, acute with perforation, without obstruction | K28.1 | Acute gastrojejunal ulcer with perforation |
|  | 534.11 | Gastrojejunal ulcer, acute with perforation, with obstruction |  |  |
|  | 534.20 | Gastrojejunal ulcer, acute with hemorrhage and perforation, without obstruction | K28.2 | Acute gastrojejunal ulcer with both hemorrhage and perforation |
|  | 534.21 | Gastrojejunal ulcer, acute with hemorrhage and perforation, with obstruction |  |  |
|  | 534.50 | Gastrojejunal ulcer, chronic or unspecified with perforation, without obstruction | K28.5 | Chronic or unspecified gastrojejunal ulcer with perforation |
|  | 534.51 | Gastrojejunal ulcer, chronic or unspecified with perforation, with obstruction |  |  |
|  | 534.60 | Gastrojejunal ulcer, chronic or unspecified with hemorrhage and perforation, without obstruction | K28.6 | Chronic or unspecified gastrojejunal ulcer with both hemorrhage and perforation |
|  | 534.61 | Gastrojejunal ulcer, chronic or unspecified with hemorrhage and perforation, with obstruction |  |  |
| **Bowel Perforation or Ischemia** | 569.83 | Perforation of intestine | K63.1 | Perforation of intestine (nontraumatic) |
|  | 550.00 | Inguinal hernia, with gangrene, unilateral or unspecified | K40.40 | Unilateral inguinal hernia, with gangrene, not specified as recurrent |
|  | 550.01 | Inguinal hernia, with gangrene, unilateral or unspecified, recurrent | K40.41 | Unilateral inguinal hernia, with gangrene, recurrent |
|  | 550.02 | Inguinal hernia, with gangrene, bilateral | K40.10 | Bilateral inguinal hernia, with gangrene, not specified as recurrent |
|  | 550.03 | Inguinal hernia, with gangrene, bilateral, recurrent | K40.11 | Bilateral inguinal hernia, with gangrene, recurrent |
|  | 551.00 | Femoral, with gangrene, unilateral | K41.40 | Unilateral femoral hernia, with gangrene, not specified as recurrent |
|  | 551.01 | Femoral, with gangrene, unilateral, recurrent | K41.41 | Unilateral femoral hernia, with gangrene, recurrent |
|  | 551.02 | Femoral, with gangrene, bilateral | K41.10 | Bilateral femoral hernia, with gangrene, not specified as recurrent |
|  | 551.03 | Femoral, with gangrene, bilateral, recurrent | K41.11 | Bilateral femoral hernia, with gangrene, recurrent |
|  | 551.1 | Umbilical hernia with gangrene | K42.1 | Umbilical hernia with gangrene |
|  | 551.20 | Ventral hernia, unspecified, with gangrene | K43.7 | Other and unspecified ventral hernia with gangrene |
|  | 551.21 | Incisional hernia, with gangrene | K43.1 | Incisional hernia with gangrene |
|  | 551.29 | Other ventral hernia, with gangrene | K43.7 | Other and unspecified ventral hernia with gangrene |
|  | 551.21 | Incisional hernia, with gangrene | K43.4 | Parastomal hernia with gangrene |
|  | 551.3  551.8 | Diaphragmatic hernia with gangrene  Hernia of other specified sties with gangrene | K44.1 | Diaphragmatic hernia with gangrene |
|  | 551.3  551.8  551.9 | Diaphragmatic hernia with gangrene  Hernia of other specified sties with gangrene  Hernia of unspecified site with gangrene | K45.1 | Other specified abdominal hernia with gangrene |
|  |  |  | K46.1 | Unspecified abdominal hernia with gangrene |
|  | 557.0 | Acute vascular insufficiency of intestine | K55.0 | Acute vascular disorders of intestine |
|  | 557.9 | Unspecified vascular insufficiency of intestine | K55.9 | Vascular disorder of intestine, unspecified |
| **Colorectal Emergencies** | | | | |
| **Infectious Colitis** | 558.2 | Toxic gastroenteritis and colitis | K52.1 | Toxic gastroenteritis and colitis |
|  | 006.2 with** | Amebic nondysenteric colitis with hemorrhage | A06.2 | Amebic nondysenteric colitis |
| **Diverticular Disease with Acute Complication** | 562.11 | Diverticulitis of colon (without mention of hemorrhage) | K57.20 | Diverticulitis of large intestine with perforation and abscess without bleeding |
|  | 569.83 | Perforation of instestine (non-traumatic) | K57.40 | Diverticulitis of both small and large intestine with performation and abscess without bleeding |
|  | 569.83 | Perforation of instestine (non-traumatic) | K57.41 | Diverticulitis of both small and large intestine with performation and abscess with bleeding |
|  | 562.11 | Diverticulitis of colon (without mention of hemorrhage) | K57.32 | Diverticulitis of large intestine without perforation or abscess without bleeding |
|  |  |  | K57.40 | Diverticulitis of both small and large intestine with perforation and abscess without bleeding |
|  |  |  | K57.52 | Diverticulitis of both small and large intestine without perforation or abscess without bleeding |
|  |  |  | K57.80 | Diverticulitis of intestine, part unspecified, with perforation and abscess without bleeding |
|  |  |  | K57.92 | Diverticulitis of intestine, part unspecified, without perforation or abscess without bleeding |
|  | 562.12 | Diverticulosis of colon with hemorrhage | K57.31 | Diverticulosis of large intestine without perforation or abscess with bleeding |
|  |  |  | K57.51 | Diverticulosis of both small and large intestine without perforation or abscess with bleeding |
|  |  |  | K57.91 | Diverticulosis of intestine, part unspecified, without perforation or abscess with bleeding |
|  | 562.13 | Diverticulitis of colon with hemorrhage | K57.21 | Diverticulitis of large intestine with perforation and abscess with bleeding |
|  |  |  | K57.33 | Diverticulitis of large intestine without perforation or abscess with bleeding |
|  |  |  | K57.41 | Diverticulitis of both small and large intestine with perforation and abscess with bleeding |
|  |  |  | K57.53 | Diverticulitis of both small and large intestine without perforation or abscess with bleeding |
|  |  |  | K57.81 | Diverticulitis of intestine, part unspecified, with perforation and abscess with bleeding |
| **Colorectal Neoplasm with GIB** | 153.1 with^ | Malignant neoplasm of transverse colon with hemorrhage | C18.4 with^^ | Malignant neoplasm of transverse colon |
|  | 153.2 with^ | Malignant neoplasm of descending colon with hemorrhage | C18.6 with^^ | Malignant neoplasm of descending colon |
|  | 153.3 with^ | Malignant neoplasm of sigmoid colon with hemorrhage | C18.7 with^^ | Malignant neoplasm of sigmoid colon |
|  | 153.4 with^ | Malignant neoplasm of cecum with hemorrhage | C18.0 with^^ | Malignant neoplasm of cecum |
|  | 153.6 with^ | Malignant neoplasm of ascending colon with hemorrhage | C18.2 with^^ | Malignant neoplasm of ascending colon |
|  | 154.0 with^ | Malignant neoplasm of rectosigmoid junction with hemorrhage | C19 with^^ | Malignant neoplasm of rectosigmoid junction |
|  | 154.1 with^ | Malignant neoplasm of rectum with hemorrhage | C20 with^^ | Malignant neoplasm of rectum |
|  | 211.3 with^ | Benign neoplasm of colon with hemorrhage | D12.0 with^^ | Benign neoplasm of cecum |
|  |  |  | D12.1 with^^ | Benign neoplasm of appendix |
|  |  |  | D12.2 with^^ | Benign neoplasm of ascending colon |
|  |  |  | D12.3 with^^ | Benign neoplasm of transverse colon |
|  |  |  | D12.4 with^^ | Benign neoplasm of descending colon |
|  |  |  | D12.5 with^^ | Benign neoplasm of sigmoid colon |
|  |  |  | D12.6 with^^ | Benign neoplasm of colon, unspecified |
|  |  |  | K63.5 with^^ | Polyp of colon |
|  | 211.4 with^ | Benign neoplasm of rectum and anal canal with hemorrhage | D12.7 with^^ | Benign neoplasm of rectosigmoid junction |
|  |  |  | D12.8 with^^ | Benign neoplasm of rectum |
|  |  |  | D12.9 with^^ | Benign neoplasm of anus and anal canal |
| **Colorectal Neoplasm with Obstruction** | 153.3 with* | Malignant neoplasm of sigmoid colon with obstruction | C18.7 with** | Malignant neoplasm of sigmoid colon with obstruction |
|  | 153.4 with* | Malignant neoplasm of cecum with obstruction | C18.0 with** | Malignant neoplasm of cecum with obstruction |
|  | 153.6 with* | Malignant neoplasm of ascending colon with obstruction | C18.2 with** | Malignant neoplasm of ascending colon with obstruction |
|  | 153.1 with* | Malignant neoplasm of the transverse colon with obstruction | C18.4 with** | Malignant neoplasm of transverse colon with obstruction |
|  | 153.2 with* | Malignant neoplasm of the descending colon with obstruction | C18.6 with** | Malignant neoplasm of descending colon with obstruction |
|  | 154.1 with* | Malignant neoplasm of the rectum with obstruction | C20 with** | Malignant neoplasm of rectum with obstruction |
| **Other Lower GIB** | 569.3 | Hemorrhage of rectum and anus | K62.5 | Hemorrhage of anus and rectum |
|  | 556.2 with^ | Ulcerative proctitis with hemorrhage | K51.211 | Ulcerative (chronic) proctitis with rectal bleeding |
|  | 556.3 with^ | Ulcerative proctosigmoiditis with hemorrhage | K51.311 | Ulcerative (chronic) rectosigmoiditis with rectal bleeding |
|  | 556.5 with^ | Left-sided ulcerative colitis with hemorrhage | K51.511 | Left sided colitis with rectal bleeding |
|  | 556.6 with^ | Universal ulcerative colitis with hemorrhage | K51.011 | Ulcerative (chronic) pancolitis with rectal bleeding |
|  | 556.8 with^ | Other ulcerative colitis with hemorrhage | K51.811 | Other ulcerative colitis with rectal bleeding |
|  | 556.9 with^ | Ulcerative colitis, unspecified, with hemorrhage | K51.911 | Ulcerative colitis, unspecified with rectal bleeding |
| **Other Colorectal Emergencies** | 560.89 | Acute Pseudo-obstruction of the intestine | K56.690 | Other partial intestinal obstruction |
|  |  |  | K56.691 | Other complete intestinal obstruction |
|  |  |  | K56.699 | Other intestinal obstruction unspecified as to partial versus complete obstruction |
|  | 560.2 | Volvulus | K56.2 | Volvulus |
|  | 569.85 | Angiodysplasia of intestine with hemorrhage | K55.21 | Angiodysplasia of colon with hemorrhage |
|  | 558.2 with^ | Toxic gastroenteritis and GI hemorrhage | K52.1 with^^ | Toxic gastroenteritis and colitis with hemorrhage |
|  | 558.9 with^ | Other and unspecified noninfectious gastroenteritis and colitis with hemorrhage | K52.89 with^^ | Other specified noninfective gastroenteritis and colitis with hemorrhage |
|  |  |  | K52.9 with^^ | Noninfective gastroenteritis and colitis, unspecified with hemorrhage |
|  | 578.9 | Hemorrhage of gastrointestinal tract, unspecified | K92.2 | Gastrointestinal hemorrhage, unspecified |
| **Diffuse Abdominal Diagnosis Not Otherwise Specified** | | | | |
| **Peritonitis** | 567.21 | Peritonitis (acute) generalized | K65.0 | Generalized (acute) peritonitis |
|  | 567.29 | Other suppurative peritonitis |  |  |
|  | 567.89 | Other specified peritonitis | K65.8 | Other peritonitis |
|  | 567.9 | Unspecified peritonitis | K65.9 | Peritonitis, unspecified |
| **Hemo-peritoneum** | 568.81 | Hemoperitoneum (nontraumatic) | K66.1 | Hemoperitoneum |
| **Solid Organ Rupture** | 289.59 | Splenic rupture, nontraumatic | D73.5 | Splenic rupture, nontraumatic |
| ^with concurrent code 578.9; ^^with concurrent code K92.2  *with concurrent code 560.9; ** with concurrent code K56.00, K56.01, K56.09, K56.90, K56.91, K56.99 | | | | |
