# Supplementary material for: Identifying the fundamental structures and processes of care contributing to emergency general surgery quality using a mixed-methods Donabedian approach
Source: BMC Med Res Methodol. 2020 Oct 2;20:247. doi: 10.1186/s12874-020-01096-7 (PMC7532630; doi:10.1186/s12874-020-01096-7)
Supplement: Supplementary file 7 — Additional file 7. ICD-9 and ICD-10 Diagnoses and Procedure Codes Used to Identify Complications. [file 12874_2020_1096_MOESM7_ESM.docx]

| **Appendix 7. ICD-9 and ICD-10 Procedure Codes For Emergency General Surgery Cases*** | | | |
| --- | --- | --- | --- |
| **ICD-9 code** | | **ICD-10 code** | |
| **Appendectomy** | | | |
| 47.01 | Laparoscopic appendectomy | 0DTJ4ZZ | Resection of Appendix, Percutaneous Endoscopic Approach |
| 47.09 | Other appendectomy | 0DTJ0ZZ | Resection of Appendix, Open Approach |
| 47.01 + V64.41 | Laparoscopic appendectomy converted to open appendectomy | 0WJG4ZZ + 0DTJ0ZZ | Inspection of Peritoneal Cavity, Percutaneous Endoscopic Approach + Resection of Appendix, Open Approach |
| **Colectomy and Colostomy** | | | |
| 45.71 | Open and other multiple segmental resection of large intestine | 0DBE0ZZ | Excision of large Intestine, open approach |
| 45.72 | Open and other cecectomy | 0DTH0ZZ | Resection of cecum, open approach |
| 45.73 | Open and other right hemicolectomy | 0DTF0ZZ | Resection of right large Intestine, open approach |
|  |  | 0DTK0ZZ | Resection of ascending colon, open approach |
| 45.74 | Other and other resection of transverse colon | 0DTL0ZZ | Resection of transverse colon, open approach |
| 45.75 | Open and other left hemicolectomy | 0DTG0ZZ | Resection of left large intestine, open approach |
| 45.76 | Open and other sigmoidectomy | 0DTN0ZZ | Resection of sigmoid colon, open approach |
| 45.79 | Other and unspecified partial excision of large intestine | 0DBE0ZZ | Excision of large Intestine, open approach |
|  |  | 0DBF0ZZ | Excision of right large Intestine, open approach |
|  |  | 0DBG0ZZ | Excision of left large Intestine, open approach |
|  |  | 0DBH0ZZ | Excision of cecum, open approach |
|  |  | 0DBK0ZZ | Excision of ascending colon, open approach |
|  |  | 0DBL0ZZ | Excision of transverse colon, open approach |
|  |  | 0DBM0ZZ | Excision of descending colon, open approach |
|  |  | 0DBN0ZZ | Excision of sigmoid colon, open approach |
|  |  | 0DTK0ZZ | Resection of ascending colon, open approach |
|  |  | 0DTM0ZZ | Resection of descending colon, open approach |
| 17.31 | Laparoscopic multiple segmental resection of large intestine | 0DBE4ZZ | Excision of large intestine, percutaneous endoscopic approach |
| 17.32 | Laparoscopic cecectomy | 0DTH4ZZ | Resection of cecum, percutaneous endoscopic approach |
| 17.33 | Laparoscopic right hemicolectomy | 0DTF4ZZ | Resection of right large intestine, percutaneous endoscopic approach |
| 17.34 | Laparoscopic resection of transverse colon | 0DTL4ZZ | Resection of transverse colon, percutaneous endoscopic approach |
| 17.35 | Laparoscopic left hemicolectomy | 0DTG4ZZ | Resection of left large intestine, percutaneous endoscopic approach |
| 17.36 | Laparoscopic sigmoidectomy | 0DTN4ZZ | Resection of sigmoid colon, percutaneous endoscopic approach |
| 17.39 | Other laparoscopic partial excision of large intestine | 0DBE4ZZ | Excision of large intestine, percutaneous endoscopic approach |
|  |  | 0DBF4ZZ | Excision of right intestine, percutaneous endoscopic approach |
|  |  | 0DBG4ZZ | Excision of left intestine, percutaneous endoscopic approach |
|  |  | 0DBH4ZZ | Excision of cecum, percutaneous endoscopic approach |
|  |  | 0DBK4ZZ | Excision of ascending colon, percutaneous endoscopic approach |
|  |  | 0DBL4ZZ | Excision of transverse colon, percutaneous endoscopic approach |
|  |  | 0DBM4ZZ | Excision of descending colon, percutaneous endoscopic approach |
|  |  | 0DBN4ZZ | Excision of sigmoid colon, percutaneous endoscopic approach |
|  |  | 0DTK4ZZ | Resection of ascending colon, percutaneous endoscopic approach |
|  |  | 0DTM4ZZ | Resection of descending colon, percutaneous endoscopic approach |
| 45.81 | Laparoscopic total intra-abdominal colectomy | 0DTE4ZZ | Resection of large intestine, percutaneous endoscopic approach |
| 45.82 | Open total intra-abdominal colectomy | 0DTE0ZZ | Resection of large intestine, open approach |
| 45.83 | Other and unspecified total intra-abdominal colectomy | 0DTE7ZZ | Resection of large intestine, via natural or artificial opening |
|  |  | 0DTE8ZZ | Resection of large Intestine, via natural or artificial opening |
| **Surgical Debridement** | | | |
| 83.09 | Other incision of soft tissue | 0J900ZZ | Drainage of scalp subcutaneous tissue and fascia, open approach |
|  |  | 0J940ZZ | Drainage of anterior neck subcutaneous tissue and fascia, open approach |
|  |  | 0J950ZZ | Drainage of posterior neck subcutaneous tissue and fascia, open approach |
|  |  | 0J960ZZ | Drainage of chest subcutaneous tissue and fascia, open approach |
|  |  | 0J970ZZ | Drainage of back subcutaneous tissue and fascia, open approach |
|  |  | 0J980ZZ | Drainage of abdomen subcutaneous tissue and fascia, open approach |
|  |  | 0J990ZZ | Drainage of buttock subcutaneous tissue and fascia, open approach |
|  |  | 0J9B0ZZ | Drainage of perineum subcutaneous tissue and fascia, open approach |
|  |  | 0J9C0ZZ | Drainage of pelvic region subcutaneous tissue and fascia, open approach |
|  |  | 0J9D0ZZ | Drainage of right upper arm subcutaneous tissue and fascia, open approach |
|  |  | 0J9F0ZZ | Drainage of left upper arm subcutaneous tissue and fascia, open approach |
|  |  | 0J9G0ZZ | Drainage of right lower arm subcutaneous tissue and fascia, open approach |
|  |  | 0J9H0ZZ | Drainage of left lower arm subcutaneous tissue and fascia, open approach |
|  |  | 0J9L0ZZ | Drainage of right upper leg subcutaneous tissue and fascia, open approach |
|  |  | 0J9M0ZZ | Drainage of left upper leg subcutaneous tissue and fascia, open approach |
|  |  | 0J9N0ZZ | Drainage of right lower leg subcutaneous tissue and fascia, open approach |
|  |  | 0J9P0ZZ | Drainage of left lower leg subcutaneous tissue and fascia, open approach |
|  |  | 0J9Q0ZZ | Drainage of right foot subcutaneous tissue and fascia, open approach |
|  |  | 0J9R0ZZ | Drainage of left foot subcutaneous tissue and fascia, open approach |
|  |  | 0KPY00Z | Removal of drainage device from lower muscle, open approach |
|  |  | 0KPY0JZ | Removal of synthetic substitute from lower muscle, open approach |
|  |  | 0KWY00Z | Revision of drainage device in lower muscle, open approach |
|  |  | 0KWY0JZ | Revision of synthetic substitute in lower muscle, open approach |
|  |  | 0KWY0MZ | Revision of Stimulator Lead in lower muscle, open approach |
| 83.44 | Other fasciectomy | 0JB00ZZ | Excision of scalp subcutaneous tissue and fascia, open approach |
|  |  | 0JB10ZZ | Excision of face subcutaneous tissue and fascia, open approach |
|  |  | 0JB40ZZ | Excision of anterior neck subcutaneous tissue and fascia, open approach |
|  |  | 0JB50ZZ | Excision of posterior neck subcutaneous tissue and fascia, open approach |
|  |  | 0JB60ZZ | Excision of chest subcutaneous tissue and fascia, open approach |
|  |  | 0JB70ZZ | Excision of back subcutaneous tissue and fascia, open approach |
|  |  | 0JB80ZZ | Excision of abdomen subcutaneous tissue and fascia, open approach |
|  |  | 0JB90ZZ | Excision of buttock subcutaneous tissue and fascia, open approach |
|  |  | 0JBB0ZZ | Excision of perineum subcutaneous tissue and fascia, open approach |
|  |  | 0JBC0ZZ | Excision of pelvic region subcutaneous tissue and fascia, open approach |
|  |  | 0JBD0ZZ | Excision of right upper arm subcutaneous tissue and fascia, open approach |
|  |  | 0JBF0ZZ | Excision of left upper arm subcutaneous tissue and fascia, open approach |
|  |  | 0JBG0ZZ | Excision of right lower arm subcutaneous tissue and fascia, open approach |
|  |  | 0JBH0ZZ | Excision of left lower arm subcutaneous tissue and fascia, open approach |
|  |  | 0JBL0ZZ | Excision of right upper leg subcutaneous tissue and fascia, open approach |
|  |  | 0JBM0ZZ | Excision of left upper leg subcutaneous tissue and fascia, open approach |
|  |  | 0JBN0ZZ | Excision of right lower leg subcutaneous tissue and fascia, open approach |
|  |  | 0JBP0ZZ | Excision of left lower leg subcutaneous tissue and fascia, open approach |
|  |  | 0JBQ0ZZ | Excision of right foot subcutaneous tissue and fascia, open approach |
|  |  | 0JBR0ZZ | Excision of left foot subcutaneous tissue and fascia, open approach |
| 83.45 | Other myectomy | 0KB00ZZ | Excision of head muscle, open approach |
|  |  | 0KB10ZZ | Excision of Facial muscle, open approach |
|  |  | 0KB20ZZ | Excision of right neck muscle, open approach |
|  |  | 0KB30ZZ | Excision of left neck muscle, open approach |
|  |  | 0KB40ZZ | Excision of Tongue, Palate, Pharynx muscle, open approach |
|  |  | 0KB50ZZ | Excision of right Shoulder muscle, open approach |
|  |  | 0KB60ZZ | Excision of left Shoulder muscle, open approach |
|  |  | 0KB70ZZ | Excision of right upper arm muscle, open approach |
|  |  | 0KB80ZZ | Excision of left upper arm muscle, open approach |
|  |  | 0KB90ZZ | Excision of right lower arm and wrist muscle, open approach |
|  |  | 0KBB0ZZ | Excision of left lower arm and wrist muscle, open approach |
|  |  | 0KBF0ZZ | Excision of right trunk muscle, open approach |
|  |  | 0KBG0ZZ | Excision of left trunk muscle, open approach |
|  |  | 0KBH0ZZ | Excision of right thorax muscle, open approach |
|  |  | 0KBJ0ZZ | Excision of left thorax muscle, open approach |
|  |  | 0KBK0ZZ | Excision of right abdomen muscle, open approach |
|  |  | 0KBL0ZZ | Excision of left abdomen muscle, open approach |
|  |  | 0KBM0ZZ | Excision of perineum muscle, open approach |
|  |  | 0KBN0ZZ | Excision of right hip muscle, open approach |
|  |  | 0KBP0ZZ | Excision of left hip muscle, open approach |
|  |  | 0KBQ0ZZ | Excision of right upper leg muscle, open approach |
|  |  | 0KBR0ZZ | Excision of left upper leg muscle, open approach |
|  |  | 0KBS0ZZ | Excision of right lower leg muscle, open approach |
|  |  | 0KBT0ZZ | Excision of left lower leg muscle, open approach |
|  |  | 0KBV0ZZ | Excision of right foot muscle, open approach |
|  |  | 0KBW0ZZ | Excision of left foot muscle, open approach |
|  |  | 0KT00ZZ | Resection of head muscle, open approach |
|  |  | 0KT10ZZ | Resection of Facial muscle, open approach |
|  |  | 0KT20ZZ | Resection of right neck muscle, open approach |
|  |  | 0KT30ZZ | Resection of left neck muscle, open approach |
|  |  | 0KT40ZZ | Resection of Tongue, Palate, Pharynx muscle, open approach |
|  |  | 0KT50ZZ | Resection of right Shoulder muscle, open approach |
|  |  | 0KT60ZZ | Resection of left Shoulder muscle, open approach |
|  |  | 0KT70ZZ | Resection of right upper arm muscle, open approach |
|  |  | 0KT80ZZ | Resection of left upper arm muscle, open approach |
|  |  | 0KT90ZZ | Resection of right lower arm and wrist muscle, open approach |
|  |  | 0KTB0ZZ | Resection of left lower arm and wrist muscle, open approach |
|  |  | 0KTF0ZZ | Resection of right trunk muscle, open approach |
|  |  | 0KTG0ZZ | Resection of left trunk muscle, open approach |
|  |  | 0KTH0ZZ | Resection of right thorax muscle, open approach |
|  |  | 0KTJ0ZZ | Resection of left thorax muscle, open approach |
|  |  | 0KTK0ZZ | Resection of right abdomen muscle, open approach |
|  |  | 0KTL0ZZ | Resection of left abdomen muscle, open approach |
|  |  | 0KTM0ZZ | Resection of perineum muscle, open approach |
|  |  | 0KTN0ZZ | Resection of right hip muscle, open approach |
|  |  | 0KTP0ZZ | Resection of left hip muscle, open approach |
|  |  | 0KTQ0ZZ | Resection of right upper leg muscle, open approach |
|  |  | 0KTR0ZZ | Resection of left upper leg muscle, open approach |
|  |  | 0KTS0ZZ | Resection of right lower leg muscle, open approach |
|  |  | 0KTT0ZZ | Resection of left lower leg muscle, open approach |
|  |  | 0KTV0ZZ | Resection of right foot muscle, open approach |
|  |  | 0KTW0ZZ | Resection of left foot muscle, open approach |
| 83.49 | Other excision of soft tissue | 0JB00ZZ | Excision of scalp subcutaneous tissue and fascia, open approach |
|  |  | 0JB10ZZ | Excision of face subcutaneous tissue and fascia, open approach |
|  |  | 0JB40ZZ | Excision of anterior neck subcutaneous tissue and fascia, open approach |
|  |  | 0JB50ZZ | Excision of posterior neck subcutaneous tissue and fascia, open approach |
|  |  | 0JB60ZZ | Excision of chest subcutaneous tissue and fascia, open approach |
|  |  | 0JB70ZZ | Excision of back subcutaneous tissue and fascia, open approach |
|  |  | 0JB80ZZ | Excision of abdomen subcutaneous tissue and fascia, open approach |
|  |  | 0JB90ZZ | Excision of buttock subcutaneous tissue and fascia, open approach |
|  |  | 0JBB0ZZ | Excision of perineum subcutaneous tissue and fascia, open approach |
|  |  | 0JBC0ZZ | Excision of pelvic region subcutaneous tissue and fascia, open approach |
|  |  | 0JBD0ZZ | Excision of right upper arm subcutaneous tissue and fascia, open approach |
|  |  | 0JBF0ZZ | Excision of left upper arm subcutaneous tissue and fascia, open approach |
|  |  | 0JBG0ZZ | Excision of right lower arm subcutaneous tissue and fascia, open approach |
|  |  | 0JBH0ZZ | Excision of left lower arm subcutaneous tissue and fascia, open approach |
|  |  | 0JBL0ZZ | Excision of right upper leg subcutaneous tissue and fascia, open approach |
|  |  | 0JBM0ZZ | Excision of left upper leg subcutaneous tissue and fascia, open approach |
|  |  | 0JBN0ZZ | Excision of right lower leg subcutaneous tissue and fascia, open approach |
|  |  | 0JBP0ZZ | Excision of left lower leg subcutaneous tissue and fascia, open approach |
|  |  | 0JBQ0ZZ | Excision of right foot subcutaneous tissue and fascia, open approach |
|  |  | 0JBR0ZZ | Excision of left foot subcutaneous tissue and fascia, open approach |
| 86.04 | Other incision with drainage of skin and subcutaneous tissue | 0H90X0Z | Drainage of scalp skin with drainage device, external approach |
|  |  | 0H90XZZ | Drainage of scalp skin, external approach |
|  |  | 0H91X0Z | Drainage of face skin with drainage device, external approach |
|  |  | 0H91XZZ | Drainage of face skin, external approach |
|  |  | 0H94X0Z | Drainage of neck skin with drainage device, external approach |
|  |  | 0H94XZZ | Drainage of neck skin, external approach |
|  |  | 0H95X0Z | Drainage of chest skin with drainage device, external approach |
|  |  | 0H95XZZ | Drainage of chest skin, external approach |
|  |  | 0H96X0Z | Drainage of back skin with drainage device, external approach |
|  |  | 0H96XZZ | Drainage of back skin, external approach |
|  |  | 0H97X0Z | Drainage of abdomen skin with drainage device, external approach |
|  |  | 0H97XZZ | Drainage of abdomen skin, external approach |
|  |  | 0H98X0Z | Drainage of buttock skin with drainage device, external approach |
|  |  | 0H98XZZ | Drainage of buttock skin, external approach |
|  |  | 0H9AX0Z | Drainage of genitalia skin with drainage device, external approach |
|  |  | 0H9AXZZ | Drainage of genitalia skin, external approach |
|  |  | 0H9BX0Z | Drainage of right upper arm skin with drainage device, external approach |
|  |  | 0H9BXZZ | Drainage of right upper arm skin, external approach |
|  |  | 0H9CX0Z | Drainage of left upper arm skin with drainage device, external approach |
|  |  | 0H9CXZZ | Drainage of left upper arm skin, external approach |
|  |  | 0H9DX0Z | Drainage of right lower arm skin with drainage device, external approach |
|  |  | 0H9DXZZ | Drainage of right lower arm skin, external approach |
|  |  | 0H9EX0Z | Drainage of left lower arm skin with drainage device, external approach |
|  |  | 0H9EXZZ | Drainage of left lower arm skin, external approach |
|  |  | 0H9FX0Z | Drainage of right hand skin with drainage device, external approach |
|  |  | 0H9FXZZ | Drainage of right hand skin, external approach |
|  |  | 0H9GX0Z | Drainage of left hand skin with drainage device, external approach |
|  |  | 0H9GXZZ | Drainage of left hand skin, external approach |
|  |  | 0H9HX0Z | Drainage of right upper leg skin with drainage device, external approach |
|  |  | 0H9HXZZ | Drainage of right upper leg skin, external approach |
|  |  | 0H9JX0Z | Drainage of left upper leg skin with drainage device, external approach |
|  |  | 0H9JXZZ | Drainage of left upper leg skin, external approach |
|  |  | 0H9KX0Z | Drainage of right lower leg skin with drainage device, external approach |
|  |  | 0H9KXZZ | Drainage of right lower leg skin, external approach |
|  |  | 0H9LX0Z | Drainage of left lower leg skin with drainage device, external approach |
|  |  | 0H9LXZZ | Drainage of left lower leg skin, external approach |
|  |  | 0H9MX0Z | Drainage of right foot skin with drainage device, external approach |
|  |  | 0H9MXZZ | Drainage of right foot skin, external approach |
|  |  | 0H9NX0Z | Drainage of left foot skin with drainage device, external approach |
|  |  | 0H9NXZZ | Drainage of left foot skin, external approach |
|  |  | 0J9000Z | Drainage of scalp subcutaneous tissue and fascia with drainage device, open approach |
|  |  | 0J9400Z | Drainage of anterior neck subcutaneous tissue and fascia with drainage device, open approach |
|  |  | 0J940ZZ | Drainage of anterior neck subcutaneous tissue and fascia, open approach |
|  |  | 0J9500Z | Drainage of posterior neck subcutaneous tissue and fascia with drainage device, open approach |
|  |  | 0J950ZZ | Drainage of posterior neck subcutaneous tissue and fascia, open approach |
|  |  | 0J9600Z | Drainage of chest subcutaneous tissue and fascia with drainage device, open approach |
|  |  | 0J960ZZ | Drainage of chest subcutaneous tissue and fascia, open approach |
|  |  | 0J9700Z | Drainage of back subcutaneous tissue and fascia with drainage device, open approach |
|  |  | 0J970ZZ | Drainage of back subcutaneous tissue and fascia, open approach |
|  |  | 0J9800Z | Drainage of abdomen subcutaneous tissue and fascia with drainage device, open approach |
|  |  | 0J980ZZ | Drainage of abdomen subcutaneous tissue and fascia, open approach |
|  |  | 0J9900Z | Drainage of buttock subcutaneous tissue and fascia with drainage device, open approach |
|  |  | 0J990ZZ | Drainage of buttock subcutaneous tissue and fascia, open approach |
|  |  | 0J9B00Z | Drainage of perineum subcutaneous tissue and fascia with drainage device, open approach |
|  |  | 0J9B0ZZ | Drainage of perineum subcutaneous tissue and fascia, open approach |
|  |  | 0J9C00Z | Drainage of pelvic region subcutaneous tissue and fascia with drainage device, open approach |
|  |  | 0J9C0ZZ | Drainage of pelvic region subcutaneous tissue and fascia, open approach |
|  |  | 0J9D00Z | Drainage of right upper arm subcutaneous tissue and fascia with drainage device, open approach |
|  |  | 0J9D0ZZ | Drainage of right upper arm subcutaneous tissue and fascia, open approach |
|  |  | 0J9F00Z | Drainage of left upper arm subcutaneous tissue and fascia with drainage device, open approach |
|  |  | 0J9F0ZZ | Drainage of left upper arm subcutaneous tissue and fascia, open approach |
|  |  | 0J9G00Z | Drainage of right lower arm subcutaneous tissue and fascia with drainage device, open approach |
|  |  | 0J9G0ZZ | Drainage of right lower arm subcutaneous tissue and fascia, open approach |
|  |  | 0J9H00Z | Drainage of left lower arm subcutaneous tissue and fascia with drainage device, open approach |
|  |  | 0J9H0ZZ | Drainage of left lower arm subcutaneous tissue and fascia, open approach |
|  |  | 0J9J00Z | Drainage of right hand subcutaneous tissue and fascia with drainage device, open approach |
|  |  | 0J9J0ZZ | Drainage of right hand subcutaneous tissue and fascia, open approach |
|  |  | 0J9K00Z | Drainage of left hand subcutaneous tissue and fascia with drainage device, open approach |
|  |  | 0J9K0ZZ | Drainage of left hand subcutaneous tissue and fascia, open approach |
|  |  | 0J9L00Z | Drainage of right upper leg subcutaneous tissue and fascia with drainage device, open approach |
|  |  | 0J9L0ZZ | Drainage of right upper leg subcutaneous tissue and fascia, open approach |
|  |  | 0J9M00Z | Drainage of left upper leg subcutaneous tissue and fascia with drainage device, open approach |
|  |  | 0J9M0ZZ | Drainage of left upper leg subcutaneous tissue and fascia, open approach |
|  |  | 0J9N00Z | Drainage of right lower leg subcutaneous tissue and fascia with drainage device, open approach |
|  |  | 0J9N0ZZ | Drainage of right lower leg subcutaneous tissue and fascia, open approach |
|  |  | 0J9P00Z | Drainage of left lower leg subcutaneous tissue and fascia with drainage device, open approach |
|  |  | 0J9P0ZZ | Drainage of left lower leg subcutaneous tissue and fascia, open approach |
|  |  | 0J9Q00Z | Drainage of right foot subcutaneous tissue and fascia with drainage device, open approach |
|  |  | 0J9Q0ZZ | Drainage of right foot subcutaneous tissue and fascia, open approach |
|  |  | 0J9R00Z | Drainage of left foot subcutaneous tissue and fascia with drainage device, open approach |
|  |  | 0J9R0ZZ | Drainage of left foot subcutaneous tissue and fascia, open approach |
|  |  | 0W9000Z | Drainage of head with drainage device, open approach |
|  |  | 0W900ZZ | Drainage of head, open approach |
|  |  | 0W9K0ZZ | Drainage of upper back, open approach |
|  |  | 0W9L00Z | Drainage of lower back with drainage device, open approach |
|  |  | 0W9L0ZZ | Drainage of lower back, open approach |
|  |  | 0W9M00Z | Drainage of male perineum with drainage device, open approach |
|  |  | 0W9M0ZZ | Drainage of male perineum, open approach |
|  |  | 0X9200Z | Drainage of right shoulder region with drainage device, open approach |
|  |  | 0X920ZZ | Drainage of right shoulder region, open approach |
|  |  | 0X9230Z | Drainage of right shoulder region with drainage device, percutaneous approach |
|  |  | 0X9300Z | Drainage of left shoulder region with drainage device, open approach |
|  |  | 0X930ZZ | Drainage of left shoulder region, open approach |
|  |  | 0X9400Z | Drainage of right axilla with drainage device, open approach |
|  |  | 0X940ZZ | Drainage of right axilla, open approach |
|  |  | 0X9500Z | Drainage of left axilla with drainage device, open approach |
|  |  | 0X950ZZ | Drainage of left axilla, open approach |
|  |  | 0X9530Z | Drainage of left axilla with drainage device, percutaneous approach |
|  |  | 0X960ZZ | Drainage of right upper extremity, open approach |
|  |  | 0X9700Z | Drainage of left upper extremity with drainage device, open approach |
|  |  | 0X970ZZ | Drainage of left upper extremity, open approach |
|  |  | 0X9800Z | Drainage of right upper arm with drainage device, open approach |
|  |  | 0X980ZZ | Drainage of right upper arm, open approach |
|  |  | 0X9900Z | Drainage of left upper arm with drainage device, open approach |
|  |  | 0X990ZZ | Drainage of left upper arm, open approach |
|  |  | 0X9B00Z | Drainage of right elbow region with drainage device, open approach |
|  |  | 0X9B0ZZ | Drainage of right elbow region, open approach |
|  |  | 0X9B30Z | Drainage of right elbow region with drainage device, percutaneous approach |
|  |  | 0X9C0ZZ | Drainage of left elbow region, open approach |
|  |  | 0X9D00Z | Drainage of right lower arm with drainage device, open approach |
|  |  | 0X9D0ZZ | Drainage of right lower arm, open approach |
|  |  | 0X9F00Z | Drainage of left lower arm with drainage device, open approach |
|  |  | 0X9F0ZZ | Drainage of left lower arm, open approach |
|  |  | 0X9G00Z | Drainage of right wrist region with drainage device, open approach |
|  |  | 0X9G0ZZ | Drainage of right wrist region, open approach |
|  |  | 0X9H00Z | Drainage of left wrist region with drainage device, open approach |
|  |  | 0X9H0ZZ | Drainage of left wrist region, open approach |
|  |  | 0Y9000Z | Drainage of right buttock with drainage device, open approach |
|  |  | 0Y900ZZ | Drainage of right buttock, open approach |
|  |  | 0Y9100Z | Drainage of left buttock with drainage device, open approach |
|  |  | 0Y910ZZ | Drainage of left buttock, open approach |
|  |  | 0Y9700Z | Drainage of right femoral region with drainage device, open approach |
|  |  | 0Y970ZZ | Drainage of right femoral region, open approach |
|  |  | 0Y9800Z | Drainage of left femoral region with drainage device, open approach |
|  |  | 0Y980ZZ | Drainage of left femoral region, open approach |
|  |  | 0Y9900Z | Drainage of right lower extremity with drainage device, open approach |
|  |  | 0Y990ZZ | Drainage of right lower extremity, open approach |
|  |  | 0Y9B00Z | Drainage of left lower extremity with drainage device, open approach |
|  |  | 0Y9B0ZZ | Drainage of left lower extremity, open approach |
|  |  | 0Y9C00Z | Drainage of right upper leg with drainage device, open approach |
|  |  | 0Y9C0ZZ | Drainage of right upper leg, open approach |
|  |  | 0Y9D00Z | Drainage of left upper leg with drainage device, open approach |
|  |  | 0Y9D0ZZ | Drainage of left upper leg, open approach |
|  |  | 0Y9F00Z | Drainage of right knee region with drainage device, open approach |
|  |  | 0Y9F0ZZ | Drainage of right knee region, open approach |
|  |  | 0Y9H00Z | Drainage of right lower leg with drainage device, open approach |
|  |  | 0Y9H0ZZ | Drainage of right lower leg, open approach |
|  |  | 0Y9J00Z | Drainage of left lower leg with drainage device, open approach |
|  |  | 0Y9J0ZZ | Drainage of left lower leg, open approach |
|  |  | 0Y9K00Z | Drainage of right ankle region with drainage device, open approach |
|  |  | 0Y9K0ZZ | Drainage of right ankle region, open approach |
|  |  | 0Y9L00Z | Drainage of left ankle region with drainage device, open approach |
|  |  | 0Y9L0ZZ | Drainage of left ankle region, open approach |
|  |  | 0Y9M00Z | Drainage of right foot with drainage device, open approach |
|  |  | 0Y9M0ZZ | Drainage of right foot, open approach |
|  |  | 0Y9N00Z | Drainage of left foot with drainage device, open approach |
|  |  | 0Y9N0ZZ | Drainage of left foot, open approach |
| 86.22 | Soft tissue excision and debridement | 0HB0XZZ | Excision of scalp skin, external approach |
|  |  | 0HB1XZZ | Excision of face skin, external approach |
|  |  | 0HB4XZZ | Excision of neck skin, external approach |
|  |  | 0HB5XZZ | Excision of chest skin, external approach |
|  |  | 0HB6XZZ | Excision of back skin, external approach |
|  |  | 0HB7XZZ | Excision of abdomen skin, external approach |
|  |  | 0HB8XZZ | Excision of buttock skin, external approach |
|  |  | 0HBAXZZ | Excision of genitalia skin, external approach |
|  |  | 0HBBXZZ | Excision of right upper arm skin, external approach |
|  |  | 0HBCXZZ | Excision of left upper arm skin, external approach |
|  |  | 0HBDXZZ | Excision of right lower arm skin, external approach |
|  |  | 0HBEXZZ | Excision of left lower arm skin, external approach |
|  |  | 0HBFXZZ | Excision of right hand skin, external approach |
|  |  | 0HBGXZZ | Excision of left hand skin, external approach |
|  |  | 0HBHXZZ | Excision of right upper leg skin, external approach |
|  |  | 0HBJXZZ | Excision of left upper leg skin, external approach |
|  |  | 0HBKXZZ | Excision of right lower leg skin, external approach |
|  |  | 0HBLXZZ | Excision of left lower leg skin, external approach |
|  |  | 0HBMXZZ | Excision of right foot skin, external approach |
|  |  | 0HBNXZZ | Excision of left foot skin, external approach |
|  |  | 0JB00ZZ | Excision of scalp subcutaneous tissue and fascia, open approach |
|  |  | 0JB10ZZ | Excision of face subcutaneous tissue and fascia, open approach |
|  |  | 0JB40ZZ | Excision of anterior neck subcutaneous tissue and fascia, open approach |
|  |  | 0JB50ZZ | Excision of posterior neck subcutaneous tissue and fascia, open approach |
|  |  | 0JB60ZZ | Excision of chest subcutaneous tissue and fascia, open approach |
|  |  | 0JB70ZZ | Excision of back subcutaneous tissue and fascia, open approach |
|  |  | 0JB80ZZ | Excision of abdomen subcutaneous tissue and fascia, open approach |
|  |  | 0JB90ZZ | Excision of buttock subcutaneous tissue and fascia, open approach |
|  |  | 0JBB0ZZ | Excision of perineum subcutaneous tissue and fascia, open approach |
|  |  | 0JBC0ZZ | Excision of pelvic region subcutaneous tissue and fascia, open approach |
|  |  | 0JBD0ZZ | Excision of right upper arm subcutaneous tissue and fascia, open approach |
|  |  | 0JBF0ZZ | Excision of left upper arm subcutaneous tissue and fascia, open approach |
|  |  | 0JBG0ZZ | Excision of right lower arm subcutaneous tissue and fascia, open approach |
|  |  | 0JBH0ZZ | Excision of left lower arm subcutaneous tissue and fascia, open approach |
|  |  | 0JBL0ZZ | Excision of right upper leg subcutaneous tissue and fascia, open approach |
|  |  | 0JBM0ZZ | Excision of left upper leg subcutaneous tissue and fascia, open approach |
|  |  | 0JBN0ZZ | Excision of right lower leg subcutaneous tissue and fascia, open approach |
|  |  | 0JBP0ZZ | Excision of left lower leg subcutaneous tissue and fascia, open approach |
|  |  | 0JBQ0ZZ | Excision of right foot subcutaneous tissue and fascia, open approach |
|  |  | 0JBR0ZZ | Excision of left foot subcutaneous tissue and fascia, open approach |
| 86.28 | Non-excisional debridement of soft tissue | 0HD0XZZ | Extraction of scalp skin, external approach |
|  |  | 0HD1XZZ | Extraction of face skin, external approach |
|  |  | 0HD4XZZ | Extraction of neck skin, external approach |
|  |  | 0HD5XZZ | Extraction of chest skin, external approach |
|  |  | 0HD6XZZ | Extraction of back skin, external approach |
|  |  | 0HD7XZZ | Extraction of abdomen skin, external approach |
|  |  | 0HD8XZZ | Extraction of buttock skin, external approach |
|  |  | 0HD9XZZ | Extraction of perineum skin, external approach |
|  |  | 0HDAXZZ | Extraction of genitalia skin, external approach |
|  |  | 0HDBXZZ | Extraction of right upper arm skin, external approach |
|  |  | 0HDCXZZ | Extraction of left upper arm skin, external approach |
|  |  | 0HDDXZZ | Extraction of right lower arm skin, external approach |
|  |  | 0HDEXZZ | Extraction of left lower arm skin, external approach |
|  |  | 0HDFXZZ | Extraction of right hand skin, external approach |
|  |  | 0HDGXZZ | Extraction of left hand skin, external approach |
|  |  | 0HDHXZZ | Extraction of right upper leg skin, external approach |
|  |  | 0HDJXZZ | Extraction of left upper leg skin, external approach |
|  |  | 0HDKXZZ | Extraction of right lower leg skin, external approach |
|  |  | 0HDLXZZ | Extraction of left lower leg skin, external approach |
|  |  | 0HDMXZZ | Extraction of right foot skin, external approach |
|  |  | 0HDNXZZ | Extraction of left foot skin, external approach |
|  |  | 0JD00ZZ | Extraction of scalp subcutaneous tissue and fascia, open approach |
|  |  | 0JD10ZZ | Extraction of face subcutaneous tissue and fascia, open approach |
|  |  | 0JD40ZZ | Extraction of anterior neck subcutaneous tissue and fascia, open approach |
|  |  | 0JD50ZZ | Extraction of posterior neck subcutaneous tissue and fascia, open approach |
|  |  | 0JD60ZZ | Extraction of chest subcutaneous tissue and fascia, open approach |
|  |  | 0JD70ZZ | Extraction of back subcutaneous tissue and fascia, open approach |
|  |  | 0JD80ZZ | Extraction of abdomen subcutaneous tissue and fascia, open approach |
|  |  | 0JD90ZZ | Extraction of buttock subcutaneous tissue and fascia, open approach |
|  |  | 0JDB0ZZ | Extraction of perineum subcutaneous tissue and fascia, open approach |
|  |  | 0JDC0ZZ | Extraction of pelvic region subcutaneous tissue and fascia, open approach |
|  |  | 0JDD0ZZ | Extraction of right upper arm subcutaneous tissue and fascia, open approach |
|  |  | 0JDF0ZZ | Extraction of left upper arm subcutaneous tissue and fascia, open approach |
|  |  | 0JDG0ZZ | Extraction of right lower arm subcutaneous tissue and fascia, open approach |
|  |  | 0JDH0ZZ | Extraction of left lower arm subcutaneous tissue and fascia, open approach |
|  |  | 0JDJ0ZZ | Extraction of right hand subcutaneous tissue and fascia, open approach |
|  |  | 0JDK0ZZ | Extraction of left hand subcutaneous tissue and fascia, open approach |
|  |  | 0JDL0ZZ | Extraction of right upper leg subcutaneous tissue and fascia, open approach |
|  |  | 0JDM0ZZ | Extraction of left upper leg subcutaneous tissue and fascia, open approach |
|  |  | 0JDN0ZZ | Extraction of right lower leg subcutaneous tissue and fascia, open approach |
|  |  | 0JDP0ZZ | Extraction of left lower leg subcutaneous tissue and fascia, open approach |
|  |  | 0JDQ0ZZ | Extraction of right foot subcutaneous tissue and fascia, open approach |
|  |  | 0JDR0ZZ | Extraction of left foot subcutaneous tissue and fascia, open approach |
| **Laparotomy** | | | |
| 54.11 | Exploratory laparotomy | 07JP0ZZ | Inspection of spleen, open approach |
|  |  | 0DJ00ZZ | Inspection of upper intestinal tract, open approach |
|  |  | 0DJ60ZZ | Inspection of stomach, open approach |
|  |  | 0DJD0ZZ | Inspection of lower intestinal tract, open approach |
|  |  | 0DJU0ZZ | Inspection of omentum, open approach |
|  |  | 0DJV0ZZ | Inspection of mesentery, open approach |
|  |  | 0DJW0ZZ | Inspection of peritoneum, open approach |
|  |  | 0WJG0ZZ | Inspection of peritoneal cavity, open approach |
|  |  | 0WJJ0ZZ | Inspection of pelvic cavity, open approach |
|  |  | 0WJP0ZZ | Inspection of gastrointestinal tract, open approach |
|  |  | 0WJR0ZZ | Inspection of genitourinary tract, open approach |
| 54.19 | Other laparotomy | 0D9S00Z | Drainage of greater omentum with drainage device, open approach |
|  |  | 0D9S0ZZ | Drainage of greater omentum, open approach |
|  |  | 0D9T00Z | Drainage of lesser omentum with drainage device, open approach |
|  |  | 0D9T0ZZ | Drainage of lesser omentum, open approach |
|  |  | 0D9V00Z | Drainage of mesentery with drainage device, open approach |
|  |  | 0D9V0ZZ | Drainage of mesentery, open approach |
|  |  | 0D9W00Z | Drainage of peritoneum with drainage device, open approach |
|  |  | 0D9W0ZZ | Drainage of peritoneum, open approach |
|  |  | 0W9G00Z | Drainage of peritoneal cavity with drainage device, open approach |
|  |  | 0W9G0ZZ | Drainage of peritoneal cavity, open approach |
| **Laparoscopy** | | | |
| 54.21 | Laparoscopy | 0DJ04ZZ | Inspection of upper intestinal tract, percutaneous endoscopic approach |
|  |  | 0DJU4ZZ | Inspection of omentum, percutaneous endoscopic approach |
|  |  | 0DJV4ZZ | Inspection of mesentery, percutaneous endoscopic approach |
|  |  | 0DJW4ZZ | Inspection of peritoneum, percutaneous endoscopic approach |
|  |  | 0FJ04ZZ | Inspection of liver, percutaneous endoscopic approach |
|  |  | 0FJ44ZZ | Inspection of gallbladder, percutaneous endoscopic approach |
|  |  | 0FJG4ZZ | Inspection of pancreas, percutaneous endoscopic approach |
|  |  | 0WJF4ZZ | Inspection of abdominal wall, percutaneous endoscopic approach |
|  |  | 0WJG4ZZ | Inspection of peritoneal cavity, percutaneous endoscopic approach |
|  |  | 0WJJ4ZZ | Inspection of pelvic cavity, percutaneous endoscopic approach |
|  |  | 0WJP4ZZ | Inspection of gastrointestinal tract, percutaneous endoscopic approach |
|  |  | 0WJR4ZZ | Inspection of genitourinary tract, percutaneous endoscopic approach |
| V64.41 | Laparoscopic converted to open | Z53.31 | Laparoscopic surgical procedure converted to open procedure |
| **Lysis of Adhesions** | | | |
| 54.51 | Laparoscopic lysis of peritoneal adhesions | 0DN84ZZ | Release small intestine, percutaneous endoscopic approach |
|  |  | 0DN94ZZ | Release ileum, percutaneous endoscopic approach |
|  |  | 0DNA4ZZ | Release jejunum, percutaneous endoscopic approach |
|  |  | 0DNB4ZZ | Release ileum, percutaneous endoscopic approach |
|  |  | 0DNC4ZZ | Release ileocecal valve, percutaneous endoscopic approach |
|  |  | 0DNE4ZZ | Release large intestine, percutaneous endoscopic approach |
|  |  | 0DNF4ZZ | Release right large intestine, percutaneous endoscopic approach |
|  |  | 0DNG4ZZ | Release left large intestine, percutaneous endoscopic approach |
|  |  | 0DNH4ZZ | Release cecum, percutaneous endoscopic approach |
|  |  | 0DNJ4ZZ | Release appendix, percutaneous endoscopic approach |
|  |  | 0DNK4ZZ | Release ascending colon, percutaneous endoscopic approach |
|  |  | 0DNL4ZZ | Release transverse colon, percutaneous endoscopic approach |
|  |  | 0DNM4ZZ | Release descending colon, percutaneous endoscopic approach |
|  |  | 0DNN4ZZ | Release sigmoid colon, percutaneous endoscopic approach |
|  |  | 0DNS4ZZ | Release greater omentum, percutaneous endoscopic approach |
|  |  | 0DNT4ZZ | Release lesser omentum, percutaneous endoscopic approach |
|  |  | 0DNV4ZZ | Release mesentery, percutaneous endoscopic approach |
|  |  | 0DNW4ZZ | Release peritoneum, percutaneous endoscopic approach |
|  |  | 0FN04ZZ | Release liver, percutaneous endoscopic approach |
|  |  | 0FN14ZZ | Release right lobe liver, percutaneous endoscopic approach |
|  |  | 0FN24ZZ | Release left lobe liver, percutaneous endoscopic approach |
|  |  | 0FN44ZZ | Release gallbladder, percutaneous endoscopic approach |
|  |  | 0FN84ZZ | Release cystic duct, percutaneous endoscopic approach |
|  |  | 0FN94ZZ | Release common bile duct, percutaneous endoscopic approach |
| 54.59 | Other lysis of peritoneal adhesions | 0DN80ZZ | Release small intestine, open approach |
|  |  | 0DN90ZZ | Release duodenum, open approach |
|  |  | 0DNA0ZZ | Release jejunum, open approach |
|  |  | 0DNB0ZZ | Release ileum, open approach |
|  |  | 0DNC0ZZ | Release ileocecal valve, open approach |
|  |  | 0DNE0ZZ | Release large intestine, open approach |
|  |  | 0DNF0ZZ | Release right large intestine, open approach |
|  |  | 0DNG0ZZ | Release left large intestine, open approach |
|  |  | 0DNH0ZZ | Release cecum, open approach |
|  |  | 0DNJ0ZZ | Release appendix, open approach |
|  |  | 0DNK0ZZ | Release ascending colon, open approach |
|  |  | 0DNL0ZZ | Release transverse colon, open approach |
|  |  | 0DNM0ZZ | Release descending colon, open approach |
|  |  | 0DNN0ZZ | Release sigmoid colon, open approach |
|  |  | 0DNS0ZZ | Release greater omentum, open approach |
|  |  | 0DNT0ZZ | Release lesser omentum, open approach |
|  |  | 0DNV0ZZ | Release mesentery, open approach |
|  |  | 0DNW0ZZ | Release peritoneum, open approach |
|  |  | 0FN00ZZ | Release liver, open approach |
|  |  | 0FN10ZZ | Release right lobe liver, open approach |
|  |  | 0FN20ZZ | Release left lobe liver, open approach |
|  |  | 0FN40ZZ | Release gallbladder, open approach |
|  |  | 0FN50ZZ | Release right hepatic duct, open approach |
|  |  | 0FN60ZZ | Release left hepatic duct, open approach |
|  |  | 0FN80ZZ | Release cystic duct, open approach |
|  |  | 0FN90ZZ | Release common bile duct, open approach |
|  |  | 0FNC0ZZ | Release ampulla of vater, open approach |
|  |  | 0FND0ZZ | Release pancreatic duct, open approach |
|  |  | 0FNF0ZZ | Release accessory pancreatic duct, open approach |
|  |  | 0FNG0ZZ | Release pancreas, open approach |
| **Small Bowel Inspection, Resection, and Ostomy** | | | |
| 45.31 | Other local excision of lesion of duodenum | 0DB90ZZ | Excision of duodenum, open approach |
| 45.61 | Multiple segmental resection of small intestine | 0DB80ZZ | Excision of small intestine, open approach |
|  |  | 0DB84ZZ | Excision of small intestine, percutaneous endoscopic approach |
| 45.62 | Other partial resection of small intestine | 0DB80ZZ | Excision of small intestine, open approach |
|  |  | 0DB84ZZ | Excision of small intestine, percutaneous endoscopic approach |
|  |  | 0DB94ZZ | Excision of duodenum, percutaneous endoscopic approach |
|  |  | 0DBA0ZZ | Excision of jejunum, open approach |
|  |  | 0DBA4ZZ | Excision of jejunum, percutaneous endoscopic approach |
|  |  | 0DBB0ZZ | Excision of ileum, open approach |
|  |  | 0DBB4ZZ | Excision of ileum, percutaneous endoscopic approach |
|  |  | 0DBC0ZZ | Excision of ileocecal valve, open approach |
|  |  | 0DBC4ZZ | Excision of ileocecal valve, percutaneous endoscopic approach |
|  |  | 0DT90ZZ | Resection of duodenum, open approach |
|  |  | 0DT94ZZ | Resection of duodenum, percutaneous endoscopic approach |
|  |  | 0DTA0ZZ | Resection of jejunum, open approach |
|  |  | 0DTA4ZZ | Resection of jejunum, percutaneous endoscopic approach |
|  |  | 0DTB0ZZ | Resection of ileum, open approach |
|  |  | 0DTB4ZZ | Resection of ileum, percutaneous endoscopic approach |
|  |  | 0DTC0ZZ | Resection of ileocecal valve, open approach |
|  |  | 0DTC4ZZ | Resection of ileocecal valve, percutaneous endoscopic approach |
| 45.63 | Total removal of small bowel | 0DT80ZZ | Resection of small intestine, open approach |
|  |  | 0DT84ZZ | Resection of small intestine, percutaneous endoscopic approach |
| 45.91 | Small to small intestinal anastomosis | 0D190Z9 | Bypass duodenum to duodenum, open approach |
|  |  | 0D190ZA | Bypass duodenum to jejunum, open approach |
|  |  | 0D190ZB | Bypass duodenum to ileum, open approach |
|  |  | 0D194Z9 | Bypass duodenum to duodenum, percutaneous endoscopic approach |
|  |  | 0D194ZA | Bypass duodenum to jejunum, percutaneous endoscopic approach |
|  |  | 0D194ZB | Bypass duodenum to ileum, percutaneous endoscopic approach |
|  |  | 0D1A0ZA | Bypass jejunum to jejunum, open approach |
|  |  | 0D1A0ZB | Bypass jejunum to ileum, open approach |
|  |  | 0D1A4ZA | Bypass jejunum to jejunum, percutaneous endoscopic approach |
|  |  | 0D1A4ZB | Bypass jejunum to ileum, percutaneous endoscopic approach |
|  |  | 0D1B0ZB | Bypass ileum to ileum, open approach |
|  |  | 0D1B4ZB | Bypass ileum to ileum, percutaneous endoscopic approach |
| 46.01 | Exteriorization of small intestine | 0D190Z4 | Bypass duodenum to cutaneous, open approach |
|  |  | 0D194Z4 | Bypass duodenum to cutaneous, percutaneous endoscopic approach |
|  |  | 0D1A0Z4 | Bypass jejunum to cutaneous, open approach |
|  |  | 0D1A4Z4 | Bypass jejunum to cutaneous, percutaneous endoscopic approach |
| 46.2 | Ileostomy, NOS | 0D1B0Z4 | Bypass ileum to cutaneous, open approach |
|  |  | 0D1B4Z4 | Bypass ileum to cutaneous, percutaneous endoscopic approach |
| 46.21 | Temporary ileostomy |  |  |
| 46.22 | Continent ileostomy |  |  |
| 46.23 | Other permanent ileostomy |  |  |
| 46.24 | Delayed opening of ileostomy | 0H87XZZ | Division of abdomen skin, external approach |
| 45.19 | Diagnostic procedures on the small intestine | 0DJ00ZZ | Inspection of upper intestinal tract, open approach |
| **Hernia Repair** | | | |
| 53.00 | Unilateral repair of inguinal hernia, NOS | 0YQ50ZZ | Repair right inguinal region, open approach |
|  |  | 0YQ54ZZ | Repair right inguinal region, percutaneous endoscopic approach |
|  |  | 0YQ60ZZ | Repair left inguinal region, open approach |
|  |  | 0YQ64ZZ | Repair left inguinal region, percutaneous endoscopic approach |
| 53.01 | Other and open repair of direct inguinal hernia | 0YQ50ZZ | Repair right inguinal region, open approach |
|  |  | 0YQ54ZZ | Repair right inguinal region, percutaneous endoscopic approach |
|  |  | 0YQ60ZZ | Repair left inguinal region, open approach |
|  |  | 0YQ64ZZ | Repair left inguinal region, percutaneous endoscopic approach |
| 53.02 | Other and open repair of indirect inguinal hernia | 0YQ50ZZ | Repair right inguinal region, open approach |
|  |  | 0YQ54ZZ | Repair right inguinal region, percutaneous endoscopic approach |
|  |  | 0YQ60ZZ | Repair left inguinal region, open approach |
|  |  | 0YQ64ZZ | Repair left inguinal region, open approach |
| 53.03 | Other and open repair of direct inguinal hernia with graft or prosthesis | 0YU50JZ | Supplement right inguinal region with synthetic substitute, open approach |
|  |  | 0YU50KZ | Supplement right inguinal region with non-autologous tissue substitute, open approach |
|  |  | 0YU60JZ | Supplement left inguinal region with synthetic substitute, open approach |
|  |  | 0YU60KZ | Supplement left inguinal region with non-autologous tissue substitute, open approach |
| 53.04 | Other and open repair of indirect inguinal hernia with graft or prosthesis | 0YU50JZ | Supplement right inguinal region with synthetic substitute, open approach |
|  |  | 0YU50KZ | Supplement right inguinal region with non-autologous tissue substitute, open approach |
|  |  | 0YU60JZ | Supplement left inguinal region with synthetic substitute, open approach |
|  |  | 0YU60KZ | Supplement left inguinal region with non-autologous tissue substitute, open approach |
| 53.05 | Repair of inguinal hernia with graft or prosthesis, NOS | 0YU50JZ | Supplement right inguinal region with synthetic substitute, open approach |
|  |  | 0YU50KZ | Supplement right inguinal region with non-autologous tissue substitute, open approach |
|  |  | 0YU60JZ | Supplement left inguinal region with synthetic substitute, open approach |
|  |  | 0YU60KZ | Supplement left inguinal region with non-autologous tissue substitute, open approach |
| 17.11 | Laparoscopic repair of direct inguinal hernia with graft or prosthesis | 0YU54JZ | Supplement right inguinal region with synthetic substitute, percutaneous endoscopic approach |
|  |  | 0YU54KZ | Supplement right inguinal region with non-autologous tissue substitute, percutaneous endoscopic approach |
|  |  | 0YU64JZ | Supplement left inguinal region with synthetic substitute, percutaneous endoscopic approach |
|  |  | 0YU64KZ | Supplement left inguinal region with non-autologous tissue substitute, percutaneous endoscopic approach |
| 17.12 | Laparoscopic repair of indirect inguinal hernia with graft or prosthesis | 0YU54JZ | Supplement right inguinal region with synthetic substitute, percutaneous endoscopic approach |
|  |  | 0YU54KZ | Supplement right inguinal region with non-autologous tissue substitute, percutaneous endoscopic approach |
|  |  | 0YU64JZ | Supplement left inguinal region with synthetic substitute, percutaneous endoscopic approach |
|  |  | 0YU64KZ | Supplement left inguinal region with non-autologous tissue substitute, percutaneous endoscopic approach |
| 17.21 | Laparoscopic bilateral repair of direct inguinal hernia with graft or prosthesis | 0YUA4JZ | Supplement bilateral inguinal region with synthetic substitute, percutaneous endoscopic approach |
|  |  | 0YUA4KZ | Supplement bilateral inguinal region with non-autologous tissue substitute, percutaneous endoscopic approach |
| 17.22 | Laparoscopic bilateral repair of indirect inguinal hernia with graft or prosthesis | 0YUA4JZ | Supplement bilateral inguinal region with synthetic substitute, percutaneous endoscopic approach |
|  |  | 0YUA4KZ | Supplement bilateral inguinal region with non-autologous tissue substitute, percutaneous endoscopic approach |
| 17.23 | Laparoscopic bilateral repair of inguinal hernia, one direct and one indirect with graft or prosthesis | 0YUA4JZ | Supplement bilateral inguinal region with synthetic substitute, percutaneous endoscopic approach |
|  |  | 0YUA4KZ | Supplement bilateral inguinal region with non-autologous tissue substitute, percutaneous endoscopic approach |
| 17.24 | Laparoscopic bilateral repair of inguinal hernia with graft or prosthesis, NOS | 0YUA4JZ | Supplement bilateral inguinal region with synthetic substitute, percutaneous endoscopic approach |
|  |  | 0YUA4KZ | Supplement bilateral inguinal region with non-autologous tissue substitute, percutaneous endoscopic approach |
| 53.10 | Bilateral repair of inguinal hernia, NOS | 0YQA0ZZ | Repair bilateral inguinal region, open approach |
|  |  | 0YQA4ZZ | Repair bilateral inguinal region, percutaneous endoscopic approach |
| 53.11 | Other and open bilateral repair of direct inguinal hernia | 0YQA0ZZ | Repair bilateral inguinal region, open approach |
|  |  | 0YQA4ZZ | Repair bilateral inguinal region, percutaneous endoscopic approach |
| 53.12 | Other and open bilateral repair of indirect inguinal hernia | 0YQA0ZZ | Repair bilateral inguinal region, open approach |
|  |  | 0YQA4ZZ | Repair bilateral inguinal region, percutaneous endoscopic approach |
| 53.13 | Other and open bilateral repair of inguinal hernia, one direct and one indirect | 0YQA0ZZ | Repair bilateral inguinal region, open approach |
|  |  | 0YQA4ZZ | Repair bilateral inguinal region, percutaneous endoscopic approach |
| 53.14 | Other and open bilateral repair of direct inguinal hernia with graft or prosthesis | 0YUA0JZ | Supplement bilateral inguinal region with synthetic substitute, open approach |
|  |  | 0YUA0KZ | Supplement bilateral inguinal region with non-autologous tissue substitute, open approach |
| 53.15 | Other and open bilateral repair of indirect inguinal hernia with graft or prosthesis | 0YUA0JZ | Supplement bilateral inguinal region with synthetic substitute, open approach |
|  |  | 0YUA0KZ | Supplement bilateral inguinal region with non-autologous tissue substitute, open approach |
| 53.16 | Other and open bilateral repair of inguinal hernia, one direct and one indirect, with graft or prosthesis | 0YUA0JZ | Supplement bilateral inguinal region with synthetic substitute, open approach |
|  |  | 0YUA0KZ | Supplement bilateral inguinal region with non-autologous tissue substitute, open approach |
| 53.17 | Bilateral repair of inguinal hernia with graft or prosthesis, NOS | 0YUA0JZ | Supplement bilateral inguinal region with synthetic substitute, open approach |
|  |  | 0YUA0KZ | Supplement bilateral inguinal region with non-autologous tissue substitute, open approach |
| 53.21 | Unilateral repair of femoral hernia with graft or prosthesis | 0YU70JZ | Supplement right femoral region with synthetic substitute, open approach |
|  |  | 0YU70KZ | Supplement right femoral region with non-autologous tissue substitute, open approach |
|  |  | 0YU74JZ | Supplement right femoral region with synthetic substitute, percutaneous endoscopic approach |
|  |  | 0YU74KZ | Supplement right femoral region with non-autologous tissue substitute, percutaneous endoscopic approach |
|  |  | 0YU80JZ | Supplement left femoral region with synthetic substitute, open approach |
|  |  | 0YU80KZ | Supplement left femoral region with non-autologous tissue substitute, open approach |
|  |  | 0YU84JZ | Supplement left femoral region with synthetic substitute, percutaneous endoscopic approach |
|  |  | 0YU84KZ | Supplement left femoral region with non-autologous tissue substitute, percutaneous endoscopic approach |
| 53.29 | Other unilateral femoral herniorrhaphy | 0YQ70ZZ | Repair right femoral region, open approach |
|  |  | 0YQ80ZZ | Repair left femoral region, open approach |
|  |  | 0YQ84ZZ | Repair left femoral region, percutaneous endoscopic approach |
| 53.31 | Bilateral repair of femoral hernia with graft or prosthesis | 0YUE0JZ | Supplement bilateral femoral region with synthetic substitute, open approach |
|  |  | 0YUE0KZ | Supplement bilateral femoral region with non-autologous tissue substitute, open approach |
|  |  | 0YUE4JZ | Supplement bilateral femoral region with synthetic substitute, percutaneous endoscopic approach |
|  |  | 0YUE4KZ | Supplement bilateral femoral region with non-autologous tissue substitute, percutaneous endoscopic approach |
| 53.39 | Other bilateral femoral herniorrhaphy | 0YQE0ZZ | Repair bilateral femoral region, open approach |
|  |  | 0YQE4ZZ | Repair bilateral femoral region, percutaneous endoscopic approach |
| 53.41 | Other and open repair of umbilical hernia with graft or prosthesis | 0WUF0JZ | Supplement abdominal wall with synthetic substitute, open approach |
|  |  | 0WUF0KZ | Supplement abdominal wall with non-autologous tissue substitute, open approach |
| 53.42 | Laparoscopic repair of umbilical hernia with graft or prosthesis | 0WUF4JZ | Supplement abdominal wall with synthetic substitute, percutaneous endoscopic approach |
|  |  | 0WUF4KZ | Supplement abdominal wall with non-autologous tissue substitute, percutaneous endoscopic approach |
| 53.43 | Other laparoscopic umbilical herniorrhaphy | 0WQF4ZZ | Repair abdominal wall, percutaneous endoscopic approach |
| 53.49 | Other open umbilical herniorrhaphy | 0WQF0ZZ | Repair abdominal wall, open approach |
| 53.51 | Incisional hernia repair | 0WQF0ZZ | Repair abdominal wall, open approach |
|  |  | 0WQF4ZZ | Repair abdominal wall, percutaneous endoscopic approach |
| 53.59 | Repair of other hernia of anterior abdominal wall | 0WQF0ZZ | Repair abdominal wall, open approach |
|  |  | 0WQF4ZZ | Repair abdominal wall, percutaneous endoscopic approach |
| 53.61 | Other open incisional hernia repair with graft or prosthesis | 0WUF0JZ | Supplement abdominal wall with synthetic substitute, open approach |
|  |  | 0WUF0KZ | Supplement abdominal wall with non-autologous tissue substitute, open approach |
| 53.62 | Laparoscopic incisional hernia repair with graft or prosthesis | 0WUF4JZ | Supplement abdominal wall with synthetic substitute, open approach |
|  |  | 0WUF4KZ | Supplement abdominal wall with non-autologous tissue substitute, open approach |
| 53.63 | Other laparoscopic repair of other hernia of anterior abdominal wall with graft or prosthesis | 0WUF4JZ | Supplement abdominal wall with synthetic substitute, open approach |
|  |  | 0WUF4KZ | Supplement abdominal wall with non-autologous tissue substitute, open approach |
| 53.69 | Other and open repair of other hernia of anterior abdominal wall with graft or prosthesis | 0WUF0JZ | Supplement abdominal wall with synthetic substitute, open approach |
|  |  | 0WUF0KZ | Supplement abdominal wall with non-autologous tissue substitute, open approach |
| 53.71 | Laparoscopic repair of diaphragmatic hernia, abdominal approach | 0BQR4ZZ | Repair right diaphragm, percutaneous endoscopic approach |
|  |  | 0BQS4ZZ | Repair left diaphragm, percutaneous endoscopic approach |
|  |  | 0BUR4JZ | Supplement right diaphragm with synthetic substitute, percutaneous endoscopic approach |
|  |  | 0BUR4KZ | substitute, percutaneous endoscopic approach |
|  |  | 0BUS4JZ | Supplement left diaphragm with synthetic substitute, percutaneous endoscopic approach |
|  |  | 0BUS4KZ | Supplement left diaphragm with non-autologous tissue substitute, percutaneous endoscopic approach |
| 53.72 | Other and open repair of diaphragmatic hernia, abdominal approach | 0BQR0ZZ | Repair right diaphragm, open approach |
|  |  | 0BQS0ZZ | Repair left diaphragm, open approach |
|  |  | 0BQS3ZZ | Repair left diaphragm, percutaneous approach |
|  |  | 0BUR0JZ | Supplement right diaphragm with synthetic substitute, open approach |
|  |  | 0BUR0KZ | Supplement right diaphragm with non-autologous tissue substitute, open approach |
|  |  | 0BUS0JZ | Supplement left diaphragm with synthetic substitute, open approach |
|  |  | 0BUS0KZ | Supplement left diaphragm with non-autologous tissue substitute, open approach |
| 53.75 | Repair of diaphragmatic hernia, abdominal approach, NOS | 0BQR4ZZ | Repair right diaphragm, percutaneous endoscopic approach |
|  |  | 0BQS0ZZ | Repair left diaphragm, open approach |
|  |  | 0BQS4ZZ | Repair left diaphragm, percutaneous endoscopic approach |
|  |  | 0BUR0JZ | Supplement right diaphragm with synthetic substitute, open approach |
|  |  | 0BUR0KZ | Supplement right diaphragm with non-autologous tissue substitute, open approach |
|  |  | 0BUR4JZ | Supplement right diaphragm with synthetic substitute, percutaneous endoscopic approach |
|  |  | 0BUR4KZ | Supplement right diaphragm with non-autologous tissue substitute, percutaneous endoscopic approach |
|  |  | 0BUS0JZ | Supplement left diaphragm with synthetic substitute, open approach |
|  |  | 0BUS0KZ | Supplement left diaphragm with non-autologous tissue substitute, open approach |
|  |  | 0BUS4JZ | Supplement left diaphragm with synthetic substitute, percutaneous endoscopic approach |
|  |  | 0BUS4KZ | Supplement left diaphragm with non-autologous tissue substitute, percutaneous endoscopic approach |
| 53.9 | Other hernia repair | 0DQS0ZZ | Repair greater omentum, open approach |
|  |  | 0DQS4ZZ | Repair greater omentum, percutaneous endoscopic approach |
|  |  | 0DQT0ZZ | Repair lesser omentum, open approach |
|  |  | 0DQT4ZZ | Repair lesser omentum, percutaneous endoscopic approach |
|  |  | 0JQ70ZZ | Repair back subcutaneous tissue and fascia, open approach |
|  |  | 0JQC0ZZ | Repair pelvic region subcutaneous tissue and fascia, open approach |
| **Ulcer Repair** | | | |
| 44.40 | Suture of peptic ulcer | 0DQ60ZZ | Repair stomach, open approach |
|  |  | 0DQ64ZZ | Repair stomach, percutaneous endoscopic approach |
| 44.41 | Suture of gastric ulcer | 0DQ60ZZ | Repair stomach, open approach |
|  |  | 0DQ64ZZ | Repair stomach, percutaneous endoscopic approach |
| 44.42 | Suture of duodenal ulcer | 0DQ90ZZ | Repair duodenum, open approach |
|  |  | 0DQ94ZZ | Repair duodenum, percutaneous endoscopic approach |
| 43.6 | Partial gastrectomy with anastomosis to duodenum (BI) | 0DB60ZZ | Excision of stomach, open approach |
|  |  | 0DB64ZZ | Excision of stomach, percutaneous endoscopic approach |
|  |  | 0DT70ZZ | Resection of stomach, pylorus, open approach |
|  |  | 0DT74ZZ | Resection of stomach, pylorus, percutaneous endoscopic approach |
|  |  | 0D160Z9 | Bypass stomach to duodenum, open approach |
|  |  | 0D164Z9 | Bypass stomach to duodenum, percutaneous endoscopic approach |
| 43.7 | Partial gastrectomy with anastomosis to jejunum (BII) | 0DB60ZZ | Excision of stomach, open approach |
|  |  | 0DB64ZZ | Excision of stomach, percutaneous endoscopic approach |
|  |  | 0D160ZA | Bypass stomach to jejunum, open approach |
|  |  | 0D164ZA | Bypass stomach to jejunum, percutaneous endoscopic approach |
| **Cholecystectomy** | | | |
| 51.21 | Other partial cholecystectomy | 0F540ZZ | Destruction of gallbladder, open approach |
|  |  | 0FB40ZZ | Excision of gallbladder, open approach |
| 51.22 | Cholecystectomy | 0FT40ZZ | Resection of gallbladder, open approach |
| 51.23 | Laparoscopic cholecystectomy | 0FT44ZZ | Resection of gallbladder, percutaneous endoscopic approach |
| 51.24 | Laparoscopic partial cholecystectomy | 0F544ZZ | Destruction of gallbladder, percutaneous endoscopic approach |
|  |  | 0FB44ZZ | Excision of gallbladder, percutaneous endoscopic approach |
| 51.23 + V64.41 dx code | Laparoscopic cholecystectomy converted to open cholecystectomy | 0FJ44ZZ + 0FT40ZZ | Inspection of gallbladder, percutaneous endoscopic approach + resection of gallbladder, open approach |
| 51.24 + V64.41 dx code | Laparoscopic cholecystectomy converted to open partial cholecystectomy | 0FJ44ZZ + 0F540ZZ | Inspection of gallbladder, percutaneous endoscopic approach + Destruction of gallbladder, open approach |
|  |  | 0FJ44ZZ + 0FB40ZZ | Inspection of gallbladder, percutaneous endoscopic approach + Excision of gallbladder, open approach |
