# Supplementary material for: Identifying the fundamental structures and processes of care contributing to emergency general surgery quality using a mixed-methods Donabedian approach
Source: BMC Med Res Methodol. 2020 Oct 2;20:247. doi: 10.1186/s12874-020-01096-7 (PMC7532630; doi:10.1186/s12874-020-01096-7)
Supplement: Supplementary file 8 — Additional file 8. ICD-9 and ICD-10 Procedure Codes for Emergency General Surgery Cases. [file 12874_2020_1096_MOESM8_ESM.docx]

| **Appendix 8: ICD-9 and ICD-10 Diagnoses and Procedure Codes Used to Identify Complications** | | | |
| --- | --- | --- | --- |
| **ICD-9 Code** | | **ICD-10 Code** | |
| **Operative Complications -** Applicable to patient who underwent an initial emergency general surgery operation | | | |
| **Unexpected Reoperation** | | | |
| 54.61^‡^ | Reclosure of postoperative disruption of abdominal wall | 0WQFXZZ | Repair Abdominal Wall, External Approach |
| 54.51^‡^ | Laparoscopic lysis of peritoneal adhesions | 0DNH4ZZ | Release Cecum, Percutaneous Endoscopic Approach |
|  |  | 0DNJ4ZZ | Release Appendix, Percutaneous Endoscopic Approach |
|  |  | 0DNK4ZZ | Release Ascending Colon, Percutaneous Endoscopic Approach |
|  |  | 0DNL4ZZ | Release Transverse Colon, Percutaneous Endoscopic Approach |
|  |  | 0DNM4ZZ | Release Descending Colon, Percutaneous Endoscopic Approach |
|  |  | 0DNN4ZZ | Release Sigmoid Colon, Percutaneous Endoscopic Approach |
|  |  | 0DNS4ZZ | Release Greater Omentum, Percutaneous Endoscopic Approach |
|  |  | 0DNT4ZZ | Release Lesser Omentum, Percutaneous Endoscopic Approach |
|  |  | 0DNV4ZZ | Release Mesentery, Percutaneous Endoscopic Approach |
|  |  | 0DNW4ZZ | Release Peritoneum, Percutaneous Endoscopic Approach |
|  |  | 0FN04ZZ | Release Liver, Percutaneous Endoscopic Approach |
|  |  | 0FN14ZZ | Release Right Lobe Liver, Percutaneous Endoscopic Approach |
|  |  | 0FN24ZZ | Release Left Lobe Liver, Percutaneous Endoscopic Approach |
|  |  | 0FN44ZZ | Release Gallbladder, Percutaneous Endoscopic Approach |
|  |  | 0FN84ZZ | Release Cystic Duct, Percutaneous Endoscopic Approach |
|  |  | 0FN94ZZ | Release Common Bile Duct, Percutaneous Endoscopic Approach |
| 54.59‡ | Other lysis peritoneal adhesions | 0DN80ZZ | Release Small Intestine, Open Approach |
|  |  | 0DN90ZZ | Release Duodenum, Open Approach |
|  |  | 0DNA0ZZ | Release Jejunum, Open Approach |
|  |  | 0DNB0ZZ | Release Ileum, Open Approach |
|  |  | 0DNC0ZZ | Release Ileocecal Valve, Open Approach |
|  |  | 0DNE0ZZ | Release Large Intestine, Open Approach |
|  |  | 0DNF0ZZ | Release Right Large Intestine, Open Approach |
|  |  | 0DNG0ZZ | Release Left Large Intestine, Open Approach |
|  |  | 0DNH0ZZ | Release Cecum, Open Approach |
|  |  | 0DNJ0ZZ | Release Appendix, Open Approach |
|  |  | 0DNK0ZZ | Release Ascending Colon, Open Approach |
|  |  | 0DNL0ZZ | Release Transverse Colon, Open Approach |
|  |  | 0DNM0ZZ | Release Descending Colon, Open Approach |
|  |  | 0DNN0ZZ | Release Sigmoid Colon, Open Approach |
|  |  | 0DNS0ZZ | Release Greater Omentum, Open Approach |
|  |  | 0DNT0ZZ | Release Lesser Omentum, Open Approach |
|  |  | 0DNV0ZZ | Release Mesentery, Open Approach |
|  |  | 0DNW0ZZ | Release Peritoneum, Open Approach |
|  |  | 0FN00ZZ | Release Liver, Open Approach |
|  |  | 0FN10ZZ | Release Right Lobe Liver, Open Approach |
|  |  | 0FN20ZZ | Release Left Lobe Liver, Open Approach |
|  |  | 0FN40ZZ | Release Gallbladder, Open Approach |
|  |  | 0FN50ZZ | Release Left Hepatic Duct, Open Approach |
|  |  | 0FN60ZZ | Release Left Hepatic Duct, Open Approach |
|  |  | 0FN80ZZ | Release Cystic Duct, Open Approach |
|  |  | 0FN90ZZ | Release Common Bile Duct, Open Approach |
|  |  | 0FNC0ZZ | Release Ampulla of Vater, Open Approach |
|  |  | 0FND0ZZ | Release Pancreatic Duct, Open Approach |
|  |  | 0FNF0ZZ | Release Accessory Pancreatic Duct, Open Approach |
|  |  | 0FNG0ZZ | Release Pancreas, Open Approach |
| 54.92^‡^ | Removal of foreign body from peritoneal cavity | 0DCS0ZZ | Extirpation of Matter from Greater Omentum, Open Approach |
|  |  | 0DCS4ZZ | Extirpation of Matter from Greater Omentum, Percutaneous Endoscopic Approach |
|  |  | 0DCT0ZZ | Extirpation of Matter from Lesser Omentum, Open Approach |
|  |  | 0DCT4ZZ | Extirpation of Matter from Lesser Omentum, Percutaneous Endoscopic Approach |
|  |  | 0DCV0ZZ | Extirpation of Matter from Mesentery, Open Approach |
|  |  | 0DCV4ZZ | Extirpation of Matter from Mesentery, Percutaneous Endoscopic Approach |
|  |  | 0DCW0ZZ | Extirpation of Matter from Peritoneum, Open Approach |
|  |  | 0DCW4ZZ | Extirpation of Matter from Peritoneum, Percutaneous Endoscopic Approach |
|  |  | 0WCG0ZZ | Extirpation of Matter from Peritoneal Cavity, Open Approach |
|  |  | 0WCG4ZZ | Extirpation of Matter from Peritoneal Cavity, Percutaneous Endoscopic Approach |
| 54.12^‡^ | Reopening of recent laparotomy site | 0W3G0ZZ | Control Bleeding in Peritoneal Cavity, Open Approach |
|  |  | 0W3H0ZZ | Control Bleeding in Retroperitoneum, Open Approach |
|  |  | 0W3P0ZZ | Control Bleeding in Gastrointestinal Tract, Open Approach |
|  |  | 0WJG0ZZ | Inspection of Peritoneal Cavity, Open Approach |
|  |  | 0WJH0ZZ | Inspection of Retroperitoneum, Open Approach |
|  |  | 0WJJ0ZZ | Inspection of Pelvic Cavity, Open Approach |
| **Deep Organ Space Infection Requiring Percutaneous Drainage** | | | |
| 54.91^‡^ | Percutaneous abdominal drainage | 0D9S30Z | Drainage of Greater Omentum with Drainage Device, Percutaneous Approach |
|  |  | 0D9S3ZZ | Drainage of Greater Omentum, Percutaneous Approach |
|  |  | 0D9S40Z | Drainage of Greater Omentum with Drainage Device, Percutaneous Endoscopic Approach |
|  |  | 0D9S4ZZ | Drainage of Greater Omentum, Percutaneous Approach |
|  |  | 0D9T30Z | Drainage of Lesser Omentum with Drainage Device, Percutaneous Approach |
|  |  | 0D9T3ZZ | Drainage of Lesser Omentum, Percutaneous Approach |
|  |  | 0D9T40Z | Drainage of Less Omentum with Drain Dev, Perc Endo Approach |
|  |  | 0D9T4ZZ | Drainage of Lesser Omentum, Percutaneous Endoscopic Approach |
|  |  | 0D9V30Z | Drainage of Mesentery with Drainage Device, Percutaneous Approach |
|  |  | 0D9V3ZZ | Drainage of Mesentery, Percutaneous Approach |
|  |  | 0D9V40Z | Drainage of Mesentery with Drainage Device, Percutaneous Endoscopic Approach |
|  |  | 0D9V4ZZ | Drainage of Mesentery, Percutaneous Endoscopic Approach |
|  |  | 0D9W30Z | Drainage of Peritoneum with Drainage Device, Percutaneous Approach |
|  |  | 0D9W3ZZ | Drainage of Peritoneum, Percutaneous Approach |
|  |  | 0D9W40Z | Drainage of Peritoneum with Drainage Device, Percutaneous Endoscopic Approach |
|  |  | 0D9W4ZZ | Drainage of Peritoneum, Percutaneous Endoscopic Approach |
|  |  | 0W9F30Z | Drainage of Abdominal Wall with Drainage Device, Percutaneous Approach |
|  |  | 0W9F3ZZ | Drainage of Abdominal Wall, Percutaneous Approach |
|  |  | 0W9F40Z | Drainage of Abdominal Wall with Drainage Device, Percutaneous Endoscopic Approach |
|  |  | 0W9F4ZZ | Drainage of Abdominal Wall, Percutaneous Endoscopic Approach |
|  |  | 0W9G30Z | Drainage of Peritoneal Cavity with Drainage Device, Percutaneous Approach |
|  |  | 0W9G3ZZ | Drainage of Peritoneal Cavity, Percutaneous Approach |
|  |  | 0W9G40Z | Drainage of Peritoneal Cavity with Drainage Device, Percutaneous Endoscopic Approach |
|  |  | 0W9G4ZZ | Drainage of Peritoneal Cavity, Percutaneous Endoscopic Approach |
|  |  | 0W9J30Z | Drainage of Pelvic Cavity with Drainage Device, Percutaneous Approach |
|  |  | 0W9J3ZZ | Drainage of Pelvic Cavity, Percutaneous Approach |
|  |  | 0W9J40Z | Drainage of Pelvic Cavity with Drainage Device, Percutaneous Endoscopic Approach |
|  |  | 0W9J4ZZ | Drainage of Pelvic Cavity, Percutaneous Endoscopic Approach |
| **Anastomotic Leak** | | | |
| 998.6 | Persistent postoperative fistula | K9189 + any other additional condition to describe clinical scenario | Other postprocedural complications and disorders of digestive system |
|  |  | T81.83XA | Persistent postprocedural fistula, initial encounter |
| **Wound Complication** | | | |
| 998.51 | Infected postoperative seroma | T81.4XXA | Infection following a procedure, initial encounter |
| 998.59 | Other postoperative infection | K68.11 | Postprocedural retroperitoneal abscess |
| 998.13 | Seroma complicating a procedure | T88.8XXA | Other specified complications of surgical and medical care, not elsewhere classified, initial encounter |
|  |  | K91.872 | Postprocedural seroma of a digestive system organ or structure following a digestive system procedure |
|  |  | K91.873 | Postprocedural seroma of a digestive system organ or structure following other procedure |
|  |  | D78.33 | Postprocedural seroma of the spleen following a procedure on the spleen |
|  |  | D78.34 | Postprocedural seroma of the spleen following other procedure |
|  |  | N99.842 | Postprocedural seroma of a genitourinary system organ or structure following a genitourinary system procedure |
|  |  | N99.843 | Postprocedural seroma of a genitourinary system organ or structure following other procedure |
|  |  | M96.842 | Postprocedural seroma of a musculoskeletal structure following a musculoskeletal system procedure |
|  |  | M96.843 | Postprocedural seroma of a musculoskeletal structure following other procedure |
|  |  | L76.34 | Postprocedural seroma of skin and subcutaneous tissue following other procedure |
|  |  | G97.64 | Postprocedural seroma of a nervous system organ or structure following other procedure |
|  |  | J95.863 | Postprocedural seroma of a respiratory system organ or structure following other procedure |
| 998.3 | Disruption of operation wound | T81.30XA | Disruption of wound, unspecified, initial encounter |
|  |  | T81.31XA | Disruption of external operation (surgical) wound, not elsewhere classified, initial encounter |
|  |  | T81.32XA | Disruption of internal operation (surgical) wound, not elsewhere classified, initial encounter |
|  |  | T81.33XA | Disruption of traumatic injury wound repair, initial encounter |
| **Hemorrhage or Hematoma** | | | |
| 998.11 | Hemorrhage complicating a procedure | D78.21 | Postprocedural hemorrhage of the spleen following a procedure on the spleen |
|  |  | D78.22 | Postprocedural hemorrhage of the spleen following other procedure |
|  |  | G97.51 | Postprocedural hemorrhage of a nervous system organ or structure following a nervous system procedure |
|  |  | G97.52 | Postprocedural hemorrhage of a nervous system organ or structure following other procedure |
|  |  | K91.840 | Postprocedural hemorrhage of a digestive system organ or structure following a digestive system procedure |
|  |  | K91.841 | Postprocedural hemorrhage of a digestive system organ or structure following other procedure |
|  |  | L76.22 | Postprocedural hemorrhage of skin and subcutaneous tissue following other procedure |
|  |  | M96.830 | Postprocedural hemorrhage of a musculoskeletal structure following a musculoskeletal system procedure |
|  |  | M96.831 | Postprocedural hemorrhage of a musculoskeletal structure following other procedure |
|  |  | N99.820 | Postprocedural hemorrhage of a genitourinary system organ or structure following a genitourinary system procedure |
|  |  | N99.821 | Postprocedural hemorrhage of a genitourinary system organ or structure following other procedure |
| 998.12 | Hematoma complicating a procedure | D78.31 | Postprocedural hematoma of the spleen following a procedure on the spleen |
|  |  | D78.32 | Postprocedural hematoma of the spleen following other procedure |
|  |  | G97.62 | Postprocedural hematoma of a nervous system organ or structure following other procedure |
|  |  | K91.870 | Postprocedural hematoma of a digestive system organ or structure following a digestive system procedure |
|  |  | K91.871 | Postprocedural hematoma of a digestive system organ or structure following other procedure |
|  |  | L76.32 | Postprocedural hematoma of skin and subcutaneous tissue following other procedure |
|  |  | M96.840 | Postprocedural hematoma of a musculoskeletal structure following a musculoskeletal system procedure |
|  |  | M96.841 | Postprocedural hematoma of a musculoskeletal structure following other procedure |
|  |  | N99.840 | Postprocedural hematoma of a genitourinary system organ or structure following a genitourinary system procedure |
|  |  | N99.841 | Postprocedural hematoma of a genitourinary system organ or structure following other procedure |
| **Bowel Obstruction** | | | |
| 560.0 | Intussusception | K56.1 | Intussusception |
| 560.1 | Paralytic ileus | K56.0 | Paralytic ileus |
|  |  | K56.7 | Ileus, unspecified |
| 560.2 | Volvulus | K56.2 | Volvulus |
| 560.32 | Fecal impaction | K56.41 | Fecal impaction |
| 560.81 | Intestinal or peritoneal adhesions with obstruction (postoperative) (postinfection) | K56.50 | Intestinal adhesions [bands], unspecified as to partial versus complete obstruction |
| 560.89 | Other partial intestinal obstruction | K56.690 | Other partial intestinal obstruction |
| 560.9 | Unspecified intestinal obstruction | K56.600 | Partial intestinal obstruction, unspecified as to cause |
| **Systemic Complications** - Applicable to all patients with or without emergency general surgery operation | | | |
| **Shock** | | | |
| 998.00 | Postoperative shock, unspecified | T81.10XA | Postprocedural shock unspecified, initial encounter |
| 998.01 | Postoperative shock, cardiogenic | T81.11XA | Postprocedural cardiogenic shock, initial encounter |
| 998.02 | Postoperative shock, septic | T81.12XA | Postprocedural septic shock, initial encounter |
| 998.09 | Postoperative shock, other | T81.19XA | Other postprocedural shock, initial encounter |
| **Cardiac** | | | |
| 410.11 | Acute myocardial infarction of other anterior wall, initial episode of care | I21.01 | ST elevation (STEMI) myocardial infarction involving left main coronary artery |
|  |  | I21.02 | ST elevation (STEMI) myocardial infarction involving left anterior descending coronary artery |
|  |  | I21.09 | ST elevation (STEMI) myocardial infarction involving other coronary artery of anterior wall |
| 410.31 | Acute myocardial infarction of inferoposterior wall, initial episode of care | I21.11 | ST elevation (STEMI) myocardial infarction involving right coronary artery |
| 410.41 | Acute myocardial infarction of other inferior wall, initial episode of care | I21.19 | ST elevation (STEMI) myocardial infarction involving other coronary artery of inferior wall |
| 410.81 | Acute myocardial infarction of other specified sites, initial episode of care | I21.21 | ST elevation (STEMI) myocardial infarction involving left circumflex coronary artery |
|  |  | I21.29 | ST elevation (STEMI) myocardial infarction involving other sites |
| 410.91 | Acute myocardial infarction of unspecified site, initial episode of care | I21.3 | ST elevation (STEMI) myocardial infarction of unspecified site |
| 410.71 | Subendocardial infarction, initial episode of care | I21.4 | Non-ST elevation (NSTEMI) myocardial infarction |
| 997.1 | Cardiac complications, not elsewhere classified | I97.111 | Postprocedural cardiac insufficiency following other surgery |
|  |  | I97.121 | Postprocedural cardiac arrest following other surgery |
|  |  | I97.131 | Postprocedural heart failure following other surgery |
|  |  | I97.191 | Other postprocedural cardiac functional disturbances following other surgery |
|  |  | I97.89 | Other postprocedural complications and disorders of the circulatory system, not elsewhere classified |
| **Pulmonary** | | | |
| 507.0 | Pneumonitis due to inhalation of food and vomit | J69.0 | Pneumonitis due to inhalation of food and vomit |
| 997.32 | Postprocedural aspiration pneumonia | J95.89 | Other postprocedural complications and disorders of respiratory system, not elsewhere classified |
| 482.9 | Bacterial pneumonia, unspecified | J15.9 | Unspecified bacterial pneumonia |
| 481 | Pneumococcal pneumonia [*Streptococcus pneumoniae* pneumonia] | J13 | Pneumonia due to *Streptococcus pneumoniae* |
|  |  | J18.1 | Lobar pneumonia, unspecified organism |
| 482.0 | Pneumonia due to *Klebsiella pneumoniae* | J15.0 | Pneumonia due to *Klebsiella pneumoniae* |
| 482.1 | Pneumonia due to *Pseudomonas* | J15.1 | Pneumonia due to *Pseudomonas* |
| 482.2 | Pneumonia due to *Hemophilus influenzae* | J14 | Pneumonia due to *Hemophilus influenzae* |
| 482.32 | Pneumonia due to streptococcus, group B | J15.3 | Pneumonia due to streptococcus, group B |
| 482.31 | Pneumonia due to Streptococcus, group A | J15.4 | Pneumonia due to other streptococci |
| 482.39 | Pneumonia due to other Streptococcus |  |  |
| 482.40 | Pneumonia due to staphylococcus, unspecified | J15.20 | Pneumonia due to staphylococcus, unspecified |
| 482.41 | Methicillin susceptible pneumonia due to *Staphylococcus aureus* | J15.211 | Pneumonia due to Methicillin susceptible *Staphylococcus aureus* |
| 482.42 | Methicillin resistant pneumonia due to *Staphylococcus aureus* | J15.212 | Pneumonia due to Methicillin resistant *Staphylococcus aureus* |
| 482.49 | Other Staphylococcus pneumonia | J15.29 | Pneumonia due to other staphylococcus |
| 482.81 | Pneumonia due to anaerobes | J15.8 | Pneumonia due to other specified bacteria |
| 482.82 | Pneumonia due to *Escherichia coli* | J15.5 | Pneumonia due to *Escherichia coli* |
| 482.83 | Pneumonia due to other Gram-negative bacteria | J15.6 | Pneumonia due to other Gram-negative bacteria |
| 482.84 | Pneumonia due to Legionnaires' disease | A48.1 | Legionnaires' disease |
| 482.89 | Pneumonia due to other specified bacteria | J15.8 | Pneumonia due to other specified bacteria |
| 482.9 | Bacterial pneumonia, unspecified | J15.9 | Unspecified bacterial pneumonia |
| 485 | Bronchopneumonia, unspecified organism | J18.0 | Bronchopneumonia, unspecified organism |
| 997.31 | Ventilator associated pneumonia | J95.851 | Ventilator associated pneumonia |
| 997.32 | Postprocedural aspiration pneumonia | J95.89 | Other postprocedural complications and disorders of respiratory system, not elsewhere classified |
| 997.39 | Other respiratory complications |  |  |
|  |  | J95.4 | Chemical pneumonitis due to anesthesia |
|  |  | J95.5 | Postprocedural subglottic stenosis |
|  |  | J95.859 | Other complication of respirator [ventilator] |
| 514 | Pulmonary congestion and hypostasis | J18.2 | Hypostatic pneumonia, unspecified organism |
|  |  | J81.1 | Chronic pulmonary edema |
| 518.4 | Acute edema of lung, unspecified | J81.0 | Acute pulmonary edema |
| 518.51 | Acute respiratory failure following trauma and surgery | J95.821 | Acute postprocedural respiratory failure |
|  |  | J96.00 | Acute respiratory failure, unspecified whether with hypoxia or hypercapnia |
|  |  | J96.01 | Acute respiratory failure with hypoxia |
|  |  | J96.02 | Acute respiratory failure with hypercapnia |
| 518.81 | Acute respiratory failure | J96.00 | Acute respiratory failure, unspecified whether with hypoxia or hypercapnia |
|  |  | J96.01 | Acute respiratory failure with hypoxia |
|  |  | J96.02 | Acute respiratory failure with hypercapnia |
|  |  | J96.90 | Respiratory failure, unspecified, unspecified whether with hypoxia or hypercapnia |
|  |  | J96.91 | Respiratory failure, unspecified with hypoxia |
|  |  | J96.92 | Respiratory failure, unspecified with hypercapnia |
| 518.82 | Other pulmonary insufficiency, not elsewhere classified | J80 | Acute respiratory distress syndrome |
| 31.1‡ | Temporary tracheostomy | 0B110F4 | Bypass Trachea to Cutaneous with Tracheostomy Device, Open Approach |
|  |  | 0B110Z4 | Bypass Trachea to Cutaneous, Open Approach |
|  |  | 0B113F4 | Bypass Trachea to Cutaneous with Tracheostomy Device, Percutaneous Approach |
|  |  | 0B113Z4 | Bypass Trachea to Cutaneous, Percutaneous Approach |
|  |  | 0B114F4 | Bypass Trachea to Cutaneous with Tracheostomy Device, Percutaneous Endoscopic Approach |
|  |  | 0B114Z4 | Bypass Trachea to Cutaneous, Percutaneous Endoscopic Approach |
| 31.29‡ | Other permanent tracheostomy | 0B110F4 | Bypass Trachea to Cutaneous with Tracheostomy Device, Open Approach |
|  |  | 0B110Z4 | Bypass Trachea to Cutaneous, Open Approach |
|  |  | 0B113F4 | Bypass Trachea to Cutaneous with Tracheostomy Device, Percutaneous Approach |
|  |  | 0B113Z4 | Bypass Trachea to Cutaneous, Percutaneous Approach |
|  |  | 0B114F4 | Bypass Trachea to Cutaneous with Tracheostomy Device, Percutaneous Endoscopic Approach |
|  |  | 0B114Z4 | Bypass Trachea to Cutaneous, Percutaneous Endoscopic Approach |
| **Neurologic** | | | |
| 431 | Intracerebral hemorrhage | I61.0 | Nontraumatic intracerebral hemorrhage in hemisphere, subcortical |
|  |  | I61.1 | Nontraumatic intracerebral hemorrhage in hemisphere, cortical |
|  |  | I61.2 | Nontraumatic intracerebral hemorrhage in hemisphere, unspecified |
|  |  | I61.3 | Nontraumatic intracerebral hemorrhage in brain stem |
|  |  | I61.4 | Nontraumatic intracerebral hemorrhage in cerebellum |
|  |  | I61.5 | Nontraumatic intracerebral hemorrhage, intraventricular |
|  |  | I61.6 | Nontraumatic intracerebral hemorrhage, multiple localized |
|  |  | I61.8 | Other nontraumatic intracerebral hemorrhage |
|  |  | I61.9 | Nontraumatic intracerebral hemorrhage, unspecified |
| 433.00 | Occlusion and stenosis of basilar artery without mention of cerebral infarction | I65.1 | Occlusion and stenosis of basilar artery |
| 433.01 | Occlusion and stenosis of basilar artery with cerebral infarction | I63.02 | Cerebral infarction due to thrombosis of basilar artery |
|  |  | I63.12 | Cerebral infarction due to embolism of basilar artery |
|  |  | I63.22 | Cerebral infarction due to unspecified occlusion or stenosis of basilar artery |
| 433.11 | Occlusion and stenosis of carotid artery with cerebral infarction | I63.031 | Cerebral infarction due to thrombosis of right carotid artery |
|  |  | I63.032 | Cerebral infarction due to thrombosis of left carotid artery |
|  |  | I63.039 | Cerebral infarction due to thrombosis of unspecified carotid artery |
|  |  | I63.131 | Cerebral infarction due to embolism of right carotid artery |
|  |  | I63.132 | Cerebral infarction due to embolism of left carotid artery |
|  |  | I63.139 | Cerebral infarction due to embolism of unspecified carotid artery |
|  |  | I63.231 | Cerebral infarction due to unspecified occlusion or stenosis of right carotid arteries |
|  |  | I63.232 | Cerebral infarction due to unspecified occlusion or stenosis of left carotid arteries |
|  |  | I63.239 | Cerebral infarction due to unspecified occlusion or stenosis of unspecified carotid artery |
| 433.21 | Occlusion and stenosis of vertebral artery with cerebral infarction | I63.011 | Cerebral infarction due to thrombosis of right vertebral artery |
|  |  | I63.012 | Cerebral infarction due to thrombosis of left vertebral artery |
|  |  | I63.019 | Cerebral infarction due to thrombosis of unspecified vertebral artery |
|  |  | I63.111 | Cerebral infarction due to embolism of right vertebral artery |
|  |  | I63.112 | Cerebral infarction due to embolism of left vertebral artery |
|  |  | I63.119 | Cerebral infarction due to embolism of unspecified vertebral artery |
|  |  | I63.211 | Cerebral infarction due to unspecified occlusion or stenosis of right vertebral artery |
|  |  | I63.212 | Cerebral infarction due to unspecified occlusion or stenosis of left vertebral artery |
|  |  | I63.219 | Cerebral infarction due to unspecified occlusion or stenosis of unspecified vertebral artery |
| 433.31 | Occlusion and stenosis of multiple and bilateral precerebral arteries with cerebral infarction | I63.59 | Cerebral infarction due to unspecified occlusion or stenosis of other cerebral artery |
| 433.81 | Occlusion and stenosis of other specified precerebral artery with cerebral infarction | I63.09 | Cerebral infarction due to thrombosis of other precerebral artery |
|  |  | I63.19 | Cerebral infarction due to embolism of other precerebral artery |
|  |  | I63.59 | Cerebral infarction due to unspecified occlusion or stenosis of other cerebral artery |
| 433.91 | Occlusion and stenosis of unspecified precerebral artery with cerebral infarction | I63.00 | Cerebral infarction due to thrombosis of unspecified precerebral artery |
|  |  | I63.10 | Cerebral infarction due to embolism of unspecified precerebral artery |
|  |  | I63.20 | Cerebral infarction due to unspecified occlusion or stenosis of unspecified precerebral arteries |
|  |  | I63.29 | Cerebral infarction due to unspecified occlusion or stenosis of other precerebral arteries |
| 434.01 | Cerebral thrombosis with cerebral infarction | I63.30 | Cerebral infarction due to thrombosis of unspecified cerebral artery |
|  |  | I63.311 | Cerebral infarction due to thrombosis of right middle cerebral artery |
|  |  | I63.312 | Cerebral infarction due to thrombosis of left middle cerebral artery |
|  |  | I63.319 | Cerebral infarction due to thrombosis of unspecified middle cerebral artery |
|  |  | I63.321 | Cerebral infarction due to thrombosis of right anterior cerebral artery |
|  |  | I63.322 | Cerebral infarction due to thrombosis of left anterior cerebral artery |
|  |  | I63.329 | Cerebral infarction due to thrombosis of unspecified anterior cerebral artery |
|  |  | I63.331 | Cerebral infarction due to thrombosis of right posterior cerebral artery |
|  |  | I63.332 | Cerebral infarction due to thrombosis of left posterior cerebral artery |
|  |  | I63.339 | Cerebral infarction due to thrombosis of unspecified posterior cerebral artery |
|  |  | I63.341 | Cerebral infarction due to thrombosis of right cerebellar artery |
|  |  | I63.342 | Cerebral infarction due to thrombosis of left cerebellar artery |
|  |  | I63.349 | Cerebral infarction due to thrombosis of unspecified cerebellar artery |
|  |  | I63.39 | Cerebral infarction due to thrombosis of other cerebral artery |
|  |  | I63.6 | Cerebral infarction due to cerebral venous thrombosis, nonpyogenic |
|  |  | I63.40 | Cerebral infarction due to embolism of unspecified cerebral artery |
| 434.11 | Cerebral embolism with cerebral infarction | I63.411 | Cerebral infarction due to embolism of right middle cerebral artery |
|  |  | I63.412 | Cerebral infarction due to embolism of left middle cerebral artery |
|  |  | I63.419 | Cerebral infarction due to embolism of unspecified middle cerebral artery |
|  |  | I63.421 | Cerebral infarction due to embolism of right anterior cerebral artery |
|  |  | I63.422 | Cerebral infarction due to embolism of left anterior cerebral artery |
|  |  | I63.429 | Cerebral infarction due to embolism of unspecified anterior cerebral artery |
|  |  | I63.431 | Cerebral infarction due to embolism of right posterior cerebral artery |
|  |  | I63.432 | Cerebral infarction due to embolism of left posterior cerebral artery |
|  |  | I63.439 | Cerebral infarction due to embolism of unspecified posterior cerebral artery |
|  |  | I63.441 | Cerebral infarction due to embolism of right cerebellar artery |
|  |  | I63.442 | Cerebral infarction due to embolism of left cerebellar artery |
|  |  | I63.449 | Cerebral infarction due to embolism of unspecified cerebellar artery |
|  |  | I63.49 | Cerebral infarction due to embolism of other cerebral artery |
| 434.91 | Cerebral artery occlusion, unspecified with cerebral infarction | I63.50 | Cerebral infarction due to unspecified occlusion or stenosis of unspecified cerebral artery |
|  |  | I63.511 | Cerebral infarction due to unspecified occlusion or stenosis of right middle cerebral artery |
|  |  | I63.512 | Cerebral infarction due to unspecified occlusion or stenosis of left middle cerebral artery |
|  |  | I63.519 | Cerebral infarction due to unspecified occlusion or stenosis of unspecified middle cerebral artery |
|  |  | I63.521 | Cerebral infarction due to unspecified occlusion or stenosis of right anterior cerebral artery |
|  |  | I63.522 | Cerebral infarction due to unspecified occlusion or stenosis of left anterior cerebral artery |
|  |  | I63.529 | Cerebral infarction due to unspecified occlusion or stenosis of unspecified anterior cerebral artery |
|  |  | I63.531 | Cerebral infarction due to unspecified occlusion or stenosis of right posterior cerebral artery |
|  |  | I63.532 | Cerebral infarction due to unspecified occlusion or stenosis of left posterior cerebral artery |
|  |  | I63.539 | Cerebral infarction due to unspecified occlusion or stenosis of unspecified posterior cerebral artery |
|  |  | I63.541 | Cerebral infarction due to unspecified occlusion or stenosis of right cerebellar artery |
|  |  | I63.542 | Cerebral infarction due to unspecified occlusion or stenosis of left cerebellar artery |
|  |  | I63.549 | Cerebral infarction due to unspecified occlusion or stenosis of unspecified cerebellar artery |
|  |  | I63.59 | Cerebral infarction due to unspecified occlusion or stenosis of other cerebral artery |
|  |  | I63.8 | Other cerebral infarction |
|  |  | I63.9 | Cerebral infarction, unspecified |
| 436 | Acute, but ill-defined, cerebrovascular disease | I67.89 | Other cerebrovascular disease |
| 437.1 | Other generalized ischemic cerebrovascular disease | I67.81 | Acute cerebrovascular insufficiency |
|  |  | I67.82 | Cerebral ischemia |
|  |  | I67.89 | Other cerebrovascular disease |
| 997.01 | Central nervous system complication | G97.82 | Other postprocedural complications and disorders of nervous system |
| 997.02 | Iatrogenic cerebrovascular infarction or hemorrhage | I97.820 | Postprocedural cerebrovascular infarction following cardiac surgery |
|  |  | I97.821 | Postprocedural cerebrovascular infarction following other surgery |
| 997.09 | Other nervous system complications | G03.8 | Meningitis due to other specified causes |
|  |  | G97.82 | Other postprocedural complications and disorders of nervous system |
| **Thromboembolic** | | | |
| 453.40 | Acute venous embolism and thrombosis of unspecified deep vessels of lower extremity | I82.401 | Acute embolism and thrombosis of unspecified deep veins of right lower extremity |
|  |  | I82.402 | Acute embolism and thrombosis of unspecified deep veins of left lower extremity |
|  |  | I82.403 | Acute embolism and thrombosis of unspecified deep veins of lower extremity, bilateral |
|  |  | I82.409 | Acute embolism and thrombosis of unspecified deep veins of unspecified lower extremity |
| 453.41 | Acute venous embolism and thrombosis of deep vessels of proximal lower extremity | I82.411 | Acute embolism and thrombosis of right femoral vein |
|  |  | I82.412 | Acute embolism and thrombosis of left femoral vein |
|  |  | I82.413 | Acute embolism and thrombosis of femoral vein, bilateral |
|  |  | I82.419 | Acute embolism and thrombosis of unspecified femoral vein |
|  |  | I82.421 | Acute embolism and thrombosis of right iliac vein |
|  |  | I82.422 | Acute embolism and thrombosis of left iliac vein |
|  |  | I82.423 | Acute embolism and thrombosis of iliac vein, bilateral |
|  |  | I82.429 | Acute embolism and thrombosis of unspecified iliac vein |
|  |  | I82.431 | Acute embolism and thrombosis of right popliteal vein |
|  |  | I82.432 | Acute embolism and thrombosis of left popliteal vein |
|  |  | I82.433 | Acute embolism and thrombosis of popliteal vein, bilateral |
|  |  | I82.439 | Acute embolism and thrombosis of unspecified popliteal vein |
|  |  | I82.4Y1 | Acute embolism and thrombosis of unspecified deep veins of right proximal lower extremity |
|  |  | I82.4Y2 | Acute embolism and thrombosis of unspecified deep veins of left proximal lower extremity |
|  |  | I82.4Y3 | Acute embolism and thrombosis of unspecified deep veins of proximal lower extremity, bilateral |
|  |  | I82.4Y9 | Acute embolism and thrombosis of unspecified deep veins of unspecified proximal lower extremity |
| 453.42 | Acute venous embolism and thrombosis of deep vessels of distal lower extremity | I82.441 | Acute embolism and thrombosis of right tibial vein |
|  |  | I82.442 | Acute embolism and thrombosis of left tibial vein |
|  |  | I82.443 | Acute embolism and thrombosis of tibial vein, bilateral |
|  |  | I82.449 | Acute embolism and thrombosis of unspecified tibial vein |
|  |  | I82.491 | Acute embolism and thrombosis of other specified deep vein of right lower extremity |
|  |  | I82.492 | Acute embolism and thrombosis of other specified deep vein of left lower extremity |
|  |  | I82.493 | Acute embolism and thrombosis of other specified deep vein of lower extremity, bilateral |
|  |  | I82.499 | Acute embolism and thrombosis of other specified deep vein of unspecified lower extremity |
|  |  | I82.4Z1 | Acute embolism and thrombosis of unspecified deep veins of right distal lower extremity |
|  |  | I82.4Z2 | Acute embolism and thrombosis of unspecified deep veins of left distal lower extremity |
|  |  | I82.4Z3 | Acute embolism and thrombosis of unspecified deep veins of distal lower extremity, bilateral |
|  |  | I82.4Z9 | Acute embolism and thrombosis of unspecified deep veins of unspecified distal lower extremity |
| 453.81 | Acute venous embolism and thrombosis of superficial veins of upper extremity | I82.611 | Acute embolism and thrombosis of superficial veins of right upper extremity |
|  |  | I82.612 | Acute embolism and thrombosis of superficial veins of left upper extremity |
|  |  | I82.613 | Acute embolism and thrombosis of superficial veins of upper extremity, bilateral |
|  |  | I82.619 | Acute embolism and thrombosis of superficial veins of unspecified upper extremity |
|  |  | I82.621 | Acute embolism and thrombosis of deep veins of right upper extremity |
|  |  | I82.622 | Acute embolism and thrombosis of deep veins of left upper extremity |
|  |  | I82.623 | Acute embolism and thrombosis of deep veins of upper extremity, bilateral |
|  |  | I82.629 | Acute embolism and thrombosis of deep veins of unspecified upper extremity |
| 453.83 | Acute venous embolism and thrombosis of upper extremity, unspecified | I82.601 | Acute embolism and thrombosis of unspecified veins of right upper extremity |
|  |  | I82.602 | Acute embolism and thrombosis of unspecified veins of left upper extremity |
|  |  | I82.603 | Acute embolism and thrombosis of unspecified veins of upper extremity, bilateral |
|  |  | I82.609 | Acute embolism and thrombosis of unspecified veins of unspecified upper extremity |
| 453.84 | Acute venous embolism and thrombosis of axillary veins | I82.A11 | Acute embolism and thrombosis of right axillary vein |
|  |  | I82.A12 | Acute embolism and thrombosis of left axillary vein |
|  |  | I82.A13 | Acute embolism and thrombosis of axillary vein, bilateral |
|  |  | I82.A19 | Acute embolism and thrombosis of unspecified axillary vein |
| 453.85 | Acute venous embolism and thrombosis of subclavian veins | I82.B11 | Acute embolism and thrombosis of right subclavian vein |
|  |  | I82.B12 | Acute embolism and thrombosis of left subclavian vein |
|  |  | I82.B13 | Acute embolism and thrombosis of subclavian vein, bilateral |
|  |  | I82.B19 | Acute embolism and thrombosis of unspecified subclavian vein |
| 453.86 | Acute venous embolism and thrombosis of internal jugular veins | I82.C11 | Acute embolism and thrombosis of right internal jugular vein |
|  |  | I82.C12 | Acute embolism and thrombosis of left internal jugular vein |
|  |  | I82.C13 | Acute embolism and thrombosis of internal jugular vein, bilateral |
|  |  | I82.C19 | Acute embolism and thrombosis of unspecified internal jugular vein |
| 453.87 | Acute venous embolism and thrombosis of other thoracic veins | I82.210 | Acute embolism and thrombosis of superior vena cava |
|  |  | I82.290 | Acute embolism and thrombosis of other thoracic veins |
| 453.89 | Acute venous embolism and thrombosis of other specified veins | I82.890 | Acute embolism and thrombosis of other specified veins |
|  |  | I82.90 | Acute embolism and thrombosis of unspecified vein |
| 453.79 | Chronic venous embolism and thrombosis of other specified veins | I82.91 | Chronic embolism and thrombosis of unspecified vein |
| 451.11 | Phlebitis and thrombophlebitis of femoral vein (deep) (superficial) | I80.10 | Phlebitis and thrombophlebitis of unspecified femoral vein |
|  |  | I80.11 | Phlebitis and thrombophlebitis of right femoral vein |
|  |  | I80.12 | Phlebitis and thrombophlebitis of left femoral vein |
|  |  | I80.13 | Phlebitis and thrombophlebitis of femoral vein, bilateral |
| 451.19 | Phlebitis and thrombophlebitis of deep veins of lower extremities, other | I80.201 | Phlebitis and thrombophlebitis of unspecified deep vessels of right lower extremity |
|  |  | I80.202 | Phlebitis and thrombophlebitis of unspecified deep vessels of left lower extremity |
|  |  | I80.203 | Phlebitis and thrombophlebitis of unspecified deep vessels of lower extremities, bilateral |
|  |  | I80.209 | Phlebitis and thrombophlebitis of unspecified deep vessels of unspecified lower extremity |
|  |  | I80.221 | Phlebitis and thrombophlebitis of right popliteal vein |
|  |  | I80.222 | Phlebitis and thrombophlebitis of left popliteal vein |
|  |  | I80.223 | Phlebitis and thrombophlebitis of popliteal vein, bilateral |
|  |  | I80.229 | Phlebitis and thrombophlebitis of unspecified popliteal vein |
|  |  | I80.231 | Phlebitis and thrombophlebitis of right tibial vein |
|  |  | I80.232 | Phlebitis and thrombophlebitis of left tibial vein |
|  |  | I80.233 | Phlebitis and thrombophlebitis of tibial vein, bilateral |
|  |  | I80.239 | Phlebitis and thrombophlebitis of unspecified tibial vein |
|  |  | I80.291 | Phlebitis and thrombophlebitis of other deep vessels of right lower extremity |
|  |  | I80.292 | Phlebitis and thrombophlebitis of other deep vessels of left lower extremity |
|  |  | I80.293 | Phlebitis and thrombophlebitis of other deep vessels of lower extremity, bilateral |
|  |  | I80.299 | Phlebitis and thrombophlebitis of other deep vessels of unspecified lower extremity |
| 451.2 | Phlebitis and thrombophlebitis of lower extremities, unspecified | I80.3 | Phlebitis and thrombophlebitis of lower extremities, unspecified |
| 451.81 | Phlebitis and thrombophlebitis of iliac vein | I80.211 | Phlebitis and thrombophlebitis of right iliac vein |
|  |  | I80.212 | Phlebitis and thrombophlebitis of left iliac vein |
|  |  | I80.213 | Phlebitis and thrombophlebitis of iliac vein, bilateral |
|  |  | I80.219 | Phlebitis and thrombophlebitis of unspecified iliac vein |
| 415.12 | Septic pulmonary embolism | I26.90 | Septic pulmonary embolism without acute cor pulmonale |
| 999.1 | Air embolism as a complication of medical care, not elsewhere classified | T80.0XXA | Air embolism following infusion, transfusion and therapeutic injection, initial encounter |
| 997.2 | Peripheral vascular complications, not elsewhere classified | T81.718A | Complication of other artery following a procedure, not elsewhere classified, initial encounter |
|  |  | T81.72XA | Complication of vein following a procedure, not elsewhere classified, initial encounter |
| 415.19 | Other pulmonary embolism and infarction | I26.99 | Other pulmonary embolism without acute cor pulmonale |
| **Renal** | | | |
| 584.5 | Acute kidney failure with tubular necrosis | N17.0 | Acute kidney failure with tubular necrosis |
| 584.6 | Acute kidney failure with lesion of renal cortical necrosis | N171 | Acute kidney failure with acute cortical necrosis |
| 584.7 | Acute kidney failure with lesion of renal medullary [papillary] necrosis | N172 | Acute kidney failure with medullary necrosis |
| 584.8 | Acute kidney failure with other specified pathological lesion in kidney | N178 | Other acute kidney failure |
| 584.9 | Acute kidney failure, unspecified | N179 | Acute kidney failure, unspecified |
| 38.95^‡^ | Venous catheterization for renal dialysis | 5A1D00Z | Performance of Urinary Filtration, Single |
|  |  | 5A1D60Z | Performance of Urinary Filtration, Multiple |
| 39.95^‡^ | Hemodialysis | 05HY33Z | Insertion of Infusion Device into Upper Vein, Percutaneous Approach |
|  |  | 06HY33Z | Insertion of Infusion Device into Lower Vein, Percutaneous Approach |
| 599.0 | Urinary tract infection, site not specified | N390 | Urinary tract infection, site not specified |
| 996.64 | Infection and inflammatory reaction due to indwelling urinary catheter | T8351XA | Infection and inflammatory reaction due to indwelling urinary catheter, initial encounter |
| 997.5 | Urinary complications, not elsewhere classified | N990 | Postprocedural (acute) (chronic) kidney failure |
|  |  | N9989 | Other postprocedural complications and disorders of genitourinary system |
| **Catheter-Associated Bloodstream Infection** | | | |
| 999.31 | Other and unspecified infection due to central venous catheter | T80218A | Other infection due to central venous catheter, initial encounter |
|  |  | T80219A | Unspecified infection due to central venous catheter, initial encounter |
| ^‡^procedure rather than diagnosis code | | | |
