# Supplementary material for: Identifying the fundamental structures and processes of care contributing to emergency general surgery quality using a mixed-methods Donabedian approach
Source: BMC Med Res Methodol. 2020 Oct 2;20:247. doi: 10.1186/s12874-020-01096-7 (PMC7532630; doi:10.1186/s12874-020-01096-7)
Supplement: Supplementary file 4 — Additional file 4. Cover Letter Included with National Survey. [file 12874_2020_1096_MOESM4_ESM.pdf]

<<EGS Letterhead>>

Date

«first» «middle» «last» «other», «degree»
«title»
«dept_division»
«location»
«hosp_institution»
«address1»
«address2»
«City», «state» «zip»

Dear Dr. «last»:

In recent years, a number of factors—ranging from workforce shortage to an aging American population—have led many to believe that there is a looming crisis in access to care for general surgery emergencies such as perforated viscus or necrotizing soft tissue infection. Since you are a leader responsible for oversight of emergency general surgery at **«Hospital_Name»**, we are contacting you to participate in a survey funded by a grant from the Agency for Healthcare Research Quality. Our goal is to further characterize this crisis, if any, and describe variations in how emergency general surgery care is delivered across the US today.

The enclosed survey asks about **«Hospital_Name»'s** current approach to emergency general surgery. If you prefer to complete the survey online using a computer, smart phone or tablet, simply log on to **http://survey.umassmed.edu/egs-survey** and enter the following user name and password:

User Name: **«USERNAME»**
Password: **«PASSWORD»**

It will take no more than 15-20 minutes to complete the survey. Your responses will be anonymously linked with routinely submitted, de-identified patient care data to determine how variations in care for general surgery emergencies impact outcomes. Results will be disseminated in aggregate form only, without identifying you or your institution. Response to this survey, either mailed or electronic, will be considered consent for participation in the study.

We realize how valuable your time is and are grateful for your willingness to complete our survey. We have included a small token of our appreciation to thank you in advance for your participation. We look forward to hearing from you. Please feel free to call 508-856-8821 or email egs-survey@umassmed.edu if you have any questions.

Sincerely,

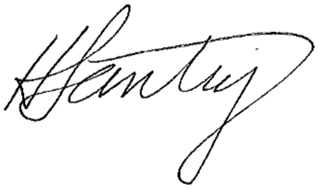

Heena P. Santry, MD MS
Assistant Professor of Surgery and Quantitative Health Sciences
University of Massachusetts Medical School

Enclosures: questionnaire, return envelope, gift

«ID_unique»
